# Hydrogen Bond Benchmark: Focal‐Point Analysis and Assessment of DFT Functionals

**DOI:** 10.1002/jcc.70265

**Published:** 2025-11-07

**Authors:** Erica C. Mitchell, Lucas Azevedo Santos, Pascal Vermeeren, Mitchell E. Lahm, Célia Fonseca Guerra, Henry F. Schaefer, F. Matthias Bickelhaupt

**Affiliations:** ^1^ Center for Computational Quantum Chemistry University of Georgia Athens Georgia USA; ^2^ Department of Chemistry and Pharmaceutical Sciences, AIMMS Vrije Universiteit Amsterdam Amsterdam the Netherlands; ^3^ Institute of Molecules and Materials Radboud University Nijmegen the Netherlands; ^4^ Department of Chemical Sciences University of Johannesburg Johannesburg South Africa

**Keywords:** coupled cluster theory, density functional theory, focal‐point analysis, hydrogen bond benchmarks

## Abstract

We performed a hierarchical, convergent ab initio benchmark study and systematically analyzed the performance of density functional approximations for describing hydrogen bonds in small neutral, cationic, and anionic complexes, as well as in larger systems involving amide, urea, deltamide, and squaramide moieties. Focal point analyses (FPA), extrapolating to the ab initio limit, were carried out using correlated wave function methods up to CCSDT(Q) for the small complexes and CCSD(T) for the larger systems, together with correlation‐consistent Gaussian basis sets up to the complete basis set limit. Optimized geometries and vibrational frequencies were obtained at the CCSD(T) level. The resulting FPA hydrogen‐bond energies converge within a few tenths of a kcal mol^−1^. These reference data were used to evaluate 60 density functionals (including 12 dispersion‐corrected), spanning the local‐density approximation (LDA), generalized gradient approximations (GGAs), meta‐GGAs, hybrids, meta‐hybrids, double‐hybrids, and range‐separated hybrids. Overall, the meta‐hybrid M06‐2X provides the best performance for both hydrogen bond energies and geometries, while the dispersion‐corrected GGAs BLYP‐D3(BJ) and BLYP‐D4 also yield accurate hydrogen‐bond data and can serve as cost‐effective options for studying large and complex systems.

## Graphical abstract

Our comprehensive hierarchical ab initio benchmark up to CCSDT(Q)/CBS in combination with a DFT performance study reveals that the meta‐hybrid M06‐2X provides the best performance for both hydrogen bond energies and geometries. The dispersion‐corrected GGAs BLYP‐D3(BJ) and BLYP‐D4 also yield accurate hydrogen‐bond data and can serve as cost‐effective alternatives for studying large and complex systems.

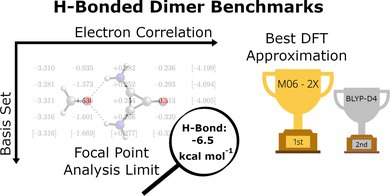

## 1 Introduction

Hydrogen bonds are among the most important interactions in biological, supramolecular, and organic chemistry, [1, 2, 3, 4, 5, 6, 7, 8] governing molecular recognition, structure, and function. Hydrogen bonding is essential for the secondary and tertiary structures of proteins [9, 10, 11], stabilizes nucleic acid base pairing [12, 13, 14, 15], plays a key role in organocatalysis [16, 17, 18, 19, 20, 21], and is widely exploited in supramolecular assemblies [22, 23, 24, 25, 26]. This bond arises when a hydrogen atom covalently bound to a more electronegative atom interacts with an electron‐rich hydrogen bond acceptor, yielding an interaction that contains both stabilizing electrostatic and covalent components [27, 28, 29, 30, 31, 32]. The strength, directionality, and geometry of hydrogen bonds are highly sensitive to the chemical characteristics of the hydrogen bond donor and acceptor, the presence of charges, and solvent effects, posing significant challenges for accurate theoretical description.

In recent decades, various studies have been dedicated to finding the best density functional approximations (DFT) for the description of hydrogen‐bonded complexes [33, 34, 35, 36, 37, 38, 39]. Most of these studies, however, analyze a large dataset that besides hydrogen bond interactions also contains several other intermolecular covalent interactions, that is, noncovalent interactions. This makes it difficult to establish if the overall best‐performing DFT also yields the most accurate hydrogen‐bonding energies. The majority of these studies also benchmark DFT relative to “single‐shot” energy reference data computed on geometries obtained at DFT or MP2 levels of theory with a small basis set (BS), without assessing convergence of electronic correlation, BS size, or geometry quality. This demonstrates the need for high‐quality reference data, specifically for hydrogen‐bond energies and geometries.

**SCHEME 1:**
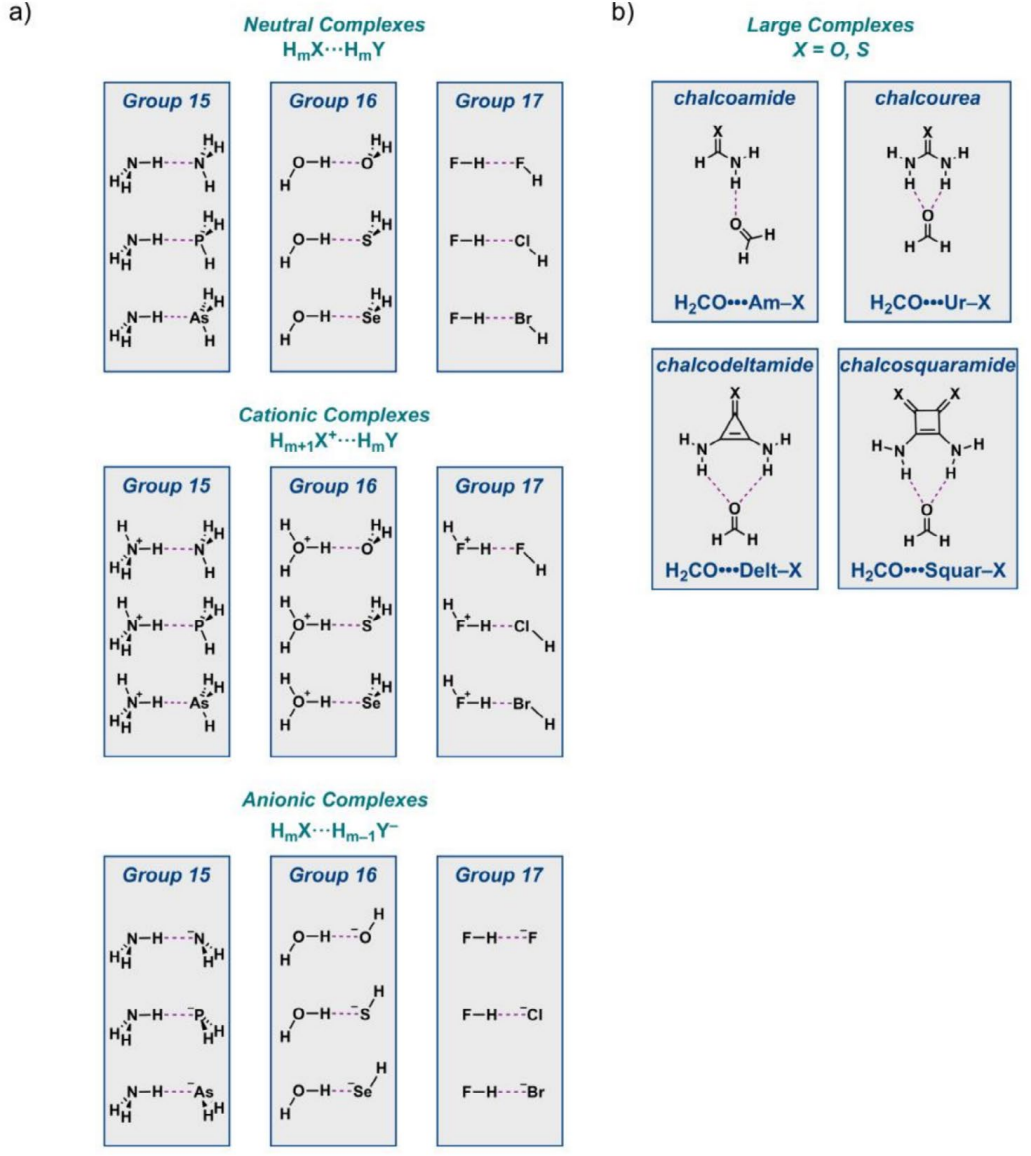
The hydrogen‐bonded complexes studied in this work: (a) neutral H_m_X•••H_m_Y, cationic H_m + 1_X^+^•••H_m_Y, and anionic H_m_X•••H_m–1_Y^−^ complexes (X = *N*, O, F; Y = *N*, P, As, O, S, Se, F, Cl, Br; m = 3, 2, 1); and (b) larger chalcoamide (**Am–X**), chalcourea (**Ur–X**), chalcodeltamide (**Delt–X**), and chalocosquaramide (**Squar–X**) with formaldehyde (H_2_CO), where X = O and S.

In this work, we analyze the hydrogen‐bond energies and geometries of two series: (i) the neutral H_m_X•••H_m_Y, cationic H_m + 1_X^+^•••H_m_Y, and anionic H_m_X•••H_m–1_Y^−^ complexes (X = N, O, F; Y = N, P, As, O, S, Se, F, Cl, Br; m = 3, 2, 1), and (ii) the large complexes involving the hydrogen bond between chalcoamide (**Am–X**), chalcourea (**Ur–X**), chalcodeltamide (**Delt–X**), and chalocosquaramide (**Squar–X**) with formaldehyde (H_2_CO), where X = O and S (Scheme 1). These series are selected because of their systematic and generic nature, covering neutral and charged hydrogen bonds (first series), and because of their actual application in organocatalysis and supramolecular chemistry (second series) [16, 17, 18, 19, 20, 21, 22, 23, 24, 25, 26, 40, 41, 42]. The purpose of this study is twofold. The first goal is to employ accurate ab initio methods to compute definitive structures and energetics for the hydrogen‐bonded complexes shown in Scheme 1. We employ the high‐level correlated wave‐function‐based method CCSD(T) to optimize the geometric structures and obtain vibrational frequencies of all complexes. To pinpoint the hydrogen‐bond energies, hierarchical focal point analyses (FPA) are performed to converge toward both the one‐ and *n*‐particle limits of ab initio quantum chemistry. This is achieved using correlation‐consistent Gaussian BS with systematically increasing flexibility and polarization up to the complete basis set (CBS) limit, together with high‐order coupled‐cluster electron correlation series up to CCSDT(Q) for the neutral, cationic, and anionic complexes, and up to CCSD(T) for the large complexes.

The second goal of this study is to utilize the reference FPA hydrogen‐bond energies to assess the performance of 60 DFT (including 12 dispersion‐corrected ones) using a TZ2P Slater‐type (STO) BS and the zeroth‐order regular approximation (ZORA) to account for relativistic effects. The functionals range in quality from the local‐density approximation (LDA) to generalized gradient approximations (GGAs), meta‐GGAs, hybrids, meta‐hybrids, double‐hybrids, and range‐separated hybrids. In addition, the geometrical accuracy of selected DFT is evaluated with respect to our accurate CCSD(T) geometries. In general, the meta‐hybrid functional M06‐2X stands out as the best overall performer for both hydrogen bond energies and geometries. The popular dispersion‐corrected GGAs BLYP‐D3(BJ) and BLYP‐D4 also provide accurate hydrogen‐bond data and hence can be used as a cost‐effective choice for studying large and complex hydrogen‐bonded systems.

## 2 Computational Methods

### 2.1 Activation‐Strain Model

The hydrogen bond energy of a complex AB composed of molecules A and B is defined as (Equation (1)):

(1) $$\text{∆}E\left(\mathrm{AB}\right)=E\left(\mathrm{AB}\right)–E\left(A\right)–E\left(B\right)$$

The hydrogen‐bond energy was decomposed using the activation strain model (ASM) into the strain energy (Δ*E*_strain_) that results from deforming the fragments A and B and the interaction energy (Δ*E*_int_) between the deformed fragments A and B [43, 44, 45]. The interaction energy of a complex AB composed of molecules A and B is defined as:

(2) $$\text{∆}E_{\mathrm{int}}\left(\mathrm{AB}\right)=E\left(\mathrm{AB}\right)-E\left(A\;\text{in}\;\mathrm{AB}\right)-E\left(B\;\text{in}\;\mathrm{AB}\right)$$

where a supermolecular approach is taken to obtain the three energies on the right‐hand side of Equation (2). However, if only atom‐centered basis functions are used, this approach can lead to artificial lowering of the energy of the complex relative to the fragments since the interacting constituent fragments in the complex can use basis functions from the other to improve their wave function whereas the separate fragments cannot [46, 47]. This artificial energy lowering is known as the basis set superposition error (BSSE). The counterpoise correction (CPC) of Boys and Bernardi mitigates this error by computing the energy of each constituent [*E*(A) and *E*(B)] within the basis of the complex [48]. Although there has been some debate on the use of the CP correction, current studies have observed that this correction is valid and recommended when using larger BS or performing CBS extrapolations [46, 47, 49].

The strain energy of a complex AB composed of molecules A and B is defined as [Equation (3)]:

(3) $$\text{∆}E_{\text{strain}}\left(\mathrm{AB}\right)=E\left(A\;\text{in}\;\mathrm{AB}\right)+E\left(B\;\text{in}\;\mathrm{AB}\right)-E\left(A\right)-E\left(B\right)$$

where the energy describes the deformation of the monomers as they form the complex.

Resultingly, in this work, the hydrogen bond energy of a complex AB composed of molecules A and B is computed as [Equation (4)]:

(4) $$\text{∆}E\left(\mathrm{AB}\right)=\text{∆}E_{\text{strain}}\left(\mathrm{AB}\right)+\text{∆}{E_{\mathrm{int}}}^{\mathrm{CP}}\left(\mathrm{AB}\right)$$

The bond energy of Equation (4) is a much better value to compare to experiment since it contains the strain energy. Especially in the complexes studied in this work, the strain energy cannot be disregarded as the hydrogen bonding will likely increase the deformation effects and hence weaken the bond energy.

### 2.2 Benchmark Geometries, Vibrational Frequencies, and Bonding Energies

All optimized geometries and harmonic vibrational frequencies were computed with Molpro 2022.1 [50, 51, 52, 53, 54, 55, 56] using coupled cluster theory with singles, doubles, and perturbative triples [CCSD(T)] [57, 58, 59, 60]. For the neutral H_m_X•••H_m_Y, cationic H_m + 1_X^+^•••H_m_Y, and anionic H_m_X•••H_m–1_Y^−^ complexes, the augmented correlation‐consistent polarized valence triple‐*ζ* BS [aug‐cc‐pVTZ] [61, 62] were utilized with the inclusion of tight‐*d* functions on third‐period atoms [aug‐cc‐pV(T + d)Z] [63, 64]. For heavier fourth‐period elements, relativistic effects were taken into consideration through the inclusion of pseudopotentials [aug‐cc‐pVTZ‐PP] [65, 66, 67], which describe the innermost electrons of those heavier elements. From this point forward, all these BS (aug‐cc‐pVTZ, aug‐cc‐pV(T + d)Z, and aug‐cc‐pVTZ‐PP) will be abbreviated as aVTZ with included effects implied by the atoms of the system.

For the computations of the larger hydrogen‐bonded complexes, a triple‐*ζ* (TZ) BS cardinality was utilized for the chalcoamide (**Am–X**) and chalcourea (**Ur–X**) complexes, but this cardinality was determined to be prohibitively expensive for the chalcodeltamide (**Delt–X**) and chalcosquaramide (**Squar–X**) complexes. Instead, the smaller double‐*ζ* (DZ) BS cardinality was used for the larger **Delt–X** and **Squar–X** complexes. These larger complexes also required the calendar BS, jul‐cc‐pVXZ (X = D, T), which removes diffuse functions on the hydrogen atoms of the system [68]. Although it is less augmented than the traditional aug‐cc‐pVXZ, this basis has repeatedly been proven to describe noncovalent interactions well with minimal error introduced [36, 69]. In addition to the jul‐cc‐pVXZ basis, important BS effects such as, tight‐*d* functions on third‐period atoms [jul‐cc‐pV(X + d)Z] were included. These BS (jul‐cc‐pVXZ and jul‐cc‐pV(X + d)Z) will be abbreviated as jul‐VXZ (X = D, T) from this point forward with added BS effects implied by the atoms of the system.

All BS and pseudopotentials were taken from the BS Exchange [70, 71, 72]. The frozen‐core (FC) approximation was used in all optimization and frequency computations. The harmonic frequencies were then used to confirm that each optimized geometry was indeed a minimum on the potential energy surface and are given in wavenumbers (cm^−1^) in the Supporting Information. Symmetry was utilized when appropriate and is listed alongside the Cartesian coordinates of the optimized geometry in the Supporting Information (Tables S1–S8). For some of the larger hydrogen‐bonded complexes (**Delt–X**, **Squar–X**), *C*_2v_ symmetry was enforced despite not being minimum geometries (Tables S15–S16) in order to mimic their supramolecular motifs more closely. Nearly all geometries for the neutral H_m_X•••H_m_Y, cationic H_m + 1_X^+^•••H_m_Y, and anionic H_m_X•••H_m–1_Y^−^ complexes were done analytically, but the NH_3_···AsH_2_^−^ complex was computed using a 3‐point finite difference scheme. All the large hydrogen‐bonded complexes also used a 3‐point finite difference scheme for the optimizations. Regardless, robust convergence criteria were enforced with the Hartree‐Fock (HF) energy and density matrix converged to 10^−10^, the coupled cluster amplitudes and lambda equations converged to 10^−10^, and the RMS gradient converged to 10^−6^ Hartree Bohr^−1^ or Hartree radian^−1^.

The CBS extrapolations presented in this work are calculated using the CP‐corrected interaction energies (∆*E*_int_^CP^). The extrapolation technique utilized is the focal‐point analysis (FPA) developed by Allen and co‐workers [73, 74, 75, 76]. This method relies on the three‐point formula of Feller to extrapolate the HF energies [Equation (5)]: [77]

(5) $$∆E_{\mathrm{HF}}\left(X\right)=E_{\mathrm{HF}}^{\infty }+ae^{-\mathrm{bX}}$$

and the two‐point formula of Helgaker to extrapolate the correlation energies [Equation (6)]: [78]

(6) $$∆E_{\text{corr}}\left(X\right)=E_{\text{corr}}^{\infty }+aX^{-3}$$

where X is the cardinality of the aVXZ or jul‐VXZ (X = D, T, Q, 5) BS from earlier. For the HartreeFock extrapolation, X = T, Q, 5 while for the correlated energies, the MP2 extrapolation uses X = Q, 5 and the CCSD and CCSD(T) extrapolations use X = T, Q. These energies were computed using Molpro 2022.1 and focal point tables for all structures are in the Supporting Information (Tables S17–S51).

In another step to reach the CBS limit for the neutral H_m_X•••H_m_Y, cationic H_m + 1_X^+^•••H_m_Y, and anionic H_m_X•••H_m–1_Y^−^ complexes, higher‐order corrections [CCSDT and CCSDT(Q)] were obtained using MRCC 2020 of Kalláy [79, 80, 81, 82, 83, 84, 85, 86]. The CCSDT correction [*δ*T] and the CCSDT(Q) correction [*δ*(Q)] used either the aVDZ or aVTZ BS dependent on computational resources. As presented in the Supporting Information (Tables S17–S51), these higher‐order corrections are nearly all under 0.1 kcal mol^−1^ with the exception of H_2_O···OH^−^. This particular complex exhibits a large *δ*T/aVTZ correction (−4.25 kcal mol^−1^) but a small *δ*(Q)/aVTZ correction (0.07 kcal mol^−1^) so to further confirm convergence a *δ*Q/aVDZ correction to the interaction energy (−0.03 kcal mol^−1^) was computed.

Further additive corrections included, for all studied systems, were a core‐correlation correction, a relativistic correction, and a diagonal Born–Oppenheimer correction (DBOC). Core‐correlation determines the validity of using the FC approximation by subtracting the all‐electron (AE) energy from the FC energy [Equation (7)]:

(7) $$\text{∆}E_{\text{core}}=\text{∆}E_{e}\left(\mathrm{AE}-\text{CCSD}\left(T\right)/\mathrm{BS}\right)-\text{∆}E_{e}\left(\mathrm{FC}-\text{CCSD}\left(T\right)/\mathrm{BS}\right),$$

which uses the core‐valence BS aug‐cc‐pCVTZ (aCVTZ) for the neutral H_m_X•••H_m_Y, cationic H_m + 1_X^+^•••H_m_Y, and anionic H_m_X•••H_m–1_Y^−^ complexes as well as for the H_2_CO*•••* **Am–X** and H_2_CO*•••* **Ur–X** complexes [61, 62, 63, 87, 88], and the weighted‐core BS (cc‐pwCVDZ) for the H_2_CO*•••* **Delt–X** and H_2_CO*•••* **Squar–X** complexes [61, 62, 87, 88]. In all complexes containing second‐ and third‐period atoms, the magnitude of the ∆*E*_core_ is below 0.20 kcal mol^−1^, justifying the use of the FC approximation. For complexes with a fourth‐period atom, the ∆*E*_core_ is larger for the strain energy with the magnitude below 0.55 kcal mol^−1^. The small magnitude of the ∆*E*_core_ shows that an expanded valence is unnecessary in computing the interaction and strain energies for systems including fourth‐period atoms [89]. A relativistic correction (∆*E*_rel_) was computed for systems that did not utilize pseudopotentials, which already account for relativistic effects. The ∆*E*_rel_ is determined as a first‐order property that is computed from the one‐electron mass‐velocity and Darwin terms at the CCSD(T) level of theory with a valence TZ BS containing the corresponding degree of augmentation mentioned earlier. Similar to the core‐correlation correction, the magnitude of the ∆*E*_rel_ is below 0.08 kcal mol^−1^ for all relevant systems. Finally, the DBOC was applied to observe the effects of the relaxation of the clamped nuclei assumption [90]. Although, it is noted that the DBOC is invalid for systems using pseudopotentials and, therefore, omitted from the appropriate complexes. For the appropriate complexes, the DBOC is computed at the HF/aVTZ level of theory. All computed DBOCs were under 0.08 kcal mol^−1^, which indicates that there are no nearby conical intersections or surface crossings. For all these additive corrections, ∆*E*_core_ was computed using Molpro 2022.1 while ∆*E*_rel_ and ∆*E*_DBOC_ used CFOUR 1.0 [91, 92].

### 2.3 Density Functional Theory (DFT) Bonding Energies and Geometries

All DFT calculations are performed using the Amsterdam Density Functional program (ADF2023.1) [93, 94, 95], as implemented in the Amsterdam Modeling Suite (AMS2023.1) software package [96]. The hydrogen bond energies were computed by performing single‐point energy calculations on the CCSD(T)/(jul‐)aVTZ geometries using a STO TZ2P BS [97] and a variety of density functionals: the LDA functional VWN [98]; the GGA functionals BP86 [99, 100], BLYP [99, 101], BEE [102, 103], PW91 [104], PBE [103], PBEsol [105], RPBE [103, 106], revPBE [103, 107], mPBE [103, 108], mPW [104, 109], HTBS [103, 110], OLYP [101, 111], OPBE [103, 111, 112], and XLYP [101, 113]; the meta‐GGA functionals M06‐L [114, 115], MVS [116], TPSS [117, 118], and revTPSS [119]; the hybrid functionals B3LYP [101, 120, 121], B3LYPs [101, 120, 122], B1LYP [101, 120, 123], B1PW91 [104, 120, 123], BHandH [124], BHandHLYP [124], KMLYP [125], O3LYP [126], OPBE0 [112], PBE0 [127, 128], mPW1PW [104, 109], mPW1K [129], S12H [130], and X3LYP [113]; the meta‐hybrid functionals M06 [114, 115], M06‐2X [114, 115], M06‐HF [114, 115], and TPSSH [117, 118]; the double‐hybrid functionals B2K‐PLYP [131], B2T‐PLYP [131], B2‐PLYP [132], LS1‐TPSS [133], mPW2K‐PLYP [131], mPW2‐PLYP [134], PBE0‐DH [135]; the range‐separated hybrid functionals CAM‐B3LYP [136], CAMY‐B3LYP [136, 137], ωB97 [138], and ωB97X [138]; and the dispersion‐corrected functionals BLYP‐D3(BJ) [99, 101, 139, 140, 141], BP86‐D4 [99, 100, 142], BLYP‐D4 [99, 101, 142], PBE‐D4 [103, 142], OLYP‐D4 [101, 111, 142], OPBE‐D4 [103, 111, 112, 142], B3LYP‐D4 [101, 120, 121, 142], PBE0‐D4 [127, 128, 142], revDSD‐BLYP‐D4 [143], revDSD‐PBE‐D4 [143], and revDSD‐PBEP86‐D4 [143]. Relativistic effects were considered using the ZORA [144, 145]. The accuracies of the fit schemes and integration grid (Becke grid) were set to VERYGOOD [146, 147]. The BSSE has been accounted for through the CPC of Boys and Bernardi [48]. The geometries of all studied hydrogen‐bonded complexes, as well as the individual fragments, have been optimized in addition using the following density functionals: DFT = OLYP, BLYP‐D3(BJ), BLYP‐D4, B3LYP, B3LYP‐D4, and M06‐2X at the ZORA‐[DFT]/TZ2P level. Each DFT stationary point was verified to be an equilibrium geometry through a harmonic vibrational analysis [148, 149, 150].

## 3 Results and Discussion

### 3.1 Benchmark Hydrogen Bonding Energetics

Table 1 presents the final extrapolated hydrogen bonding energies of the neutral H_m_X•••H_m_Y, cationic H_m + 1_X^+^•••H_m_Y, and anionic H_m_X•••H_m–1_Y^−^ complexes (X = N, O, F; Y = N, P, As, O, S, Se, F, Cl, Br; m = 3, 2, 1) as well as the large complexes involving the hydrogen bond between chalcoamide (**Am–X**), chalcourea (**Ur–X**), chalcodeltamide (**Delt–X**), and chalocosquaramide (**Squar–X**) with formaldehyde (H_2_CO), where X = O and S, emerging from our focal‐point analyses, are presented in Table 1. The complete focal‐point analyses (FPA) of all systems studied in this work can be found in Supporting Information Tables S9–S45. From FPA, smooth monotonic convergence to the CBS and electron correlation limits was observed for all complexes with only H_2_O···OH^−^ requiring beyond a δ(Q) correction. Thus, the final bond energies (*∆E*) target CCSDT(Q)/CBS quality for the neutral H_m_X•••H_m_Y, cationic H_m + 1_X^+^•••H_m_Y, and anionic H_m_X•••H_m–1_Y^−^ complexes and CCSD(T)/CBS quality for the larger hydrogen‐bonded complexes.

**TABLE 1:** Final extrapolated hydrogen bond energies (∆*E*, in kcal mol^−1^) targeting the CCSDT(Q)/CBS limit for the neutral H_m_X•••H_m_Y, cationic H_m + 1_X^+^•••H_m_Y, and anionic H_m_X•••H_m–1_Y^−^ complexes and CCSD(T)/CBS limit for the large complexes, including a core‐correlation correction, a relativistic correction, and the diagonal Born‐Oppenheimer correction where appropriate.^a^ ^,^ ^b^ ^,^ ^c^

Neutral H_m_X•••H_m_Y complexes					
Complex	Δ*E*	Complex	Δ*E*	Complex	Δ*E*
NH_3_ *•••*NH_3_	−3.10	H_2_O•••H_2_O	−5.02	HF•••HF	−4.61
NH_3_ *•••*PH_3_	−1.79	H_2_O•••H_2_S	−3.02	HF•••HCl	−3.06
NH_3_ *•••*AsH_3_	^d^	H_2_O•••H_2_Se	−2.94	HF•••HBr	−3.02

Cationic H_m + 1_X^+^•••H_m_Y complexes					
Complex	Δ*E*	Complex	Δ*E*	Complex	Δ*E*
NH_4_^+^ *•••*NH_3_	−26.25	H_3_O^+^•••H_2_O	−33.75	H_2_F^+^•••HF	−32.74
NH_4_^+^ *•••*PH_3_	−14.11	H_3_O^+^•••H_2_S	−24.07	H_2_F^+^•••HCl	−34.52
NH_4_^+^ *•••*AsH_3_	−12.29	H_3_O^+^•••H_2_Se	−24.06	H_2_F^+^•••HBr	−36.54

Anionic H_m_X•••H_m–1_Y^−1^ complexes					
Complex	Δ*E*	Complex	Δ*E*	Complex	Δ*E*
NH_3_ *•••*NH_2_^−^	−13.75	H_2_O•••OH^−^	−26.89	HF•••F^−^	−44.04
NH_3_ *•••*PH_2_^−^	−7.65	H_2_O•••SH^−^	−14.99	HF•••Cl^−^	−23.76
NH_3_ *•••*AsH_2_^−^	−7.31	H_2_O•••SeH^−^	−13.59	HF•••Br^−^	−20.47

Large complexes			
Complex	Δ*E*	Complex	Δ*E*
H_2_CO*•••* **Am–O**	−4.95	H_2_CO*•••* **Am–S**	−5.59
H_2_CO*•••* **Ur–O**	−5.56	H_2_CO*•••* **Ur–S**	−6.93
H_2_CO*•••* **Ur–O** ^e^	−6.33	H_2_CO*•••* **Ur–S** ^e^	−7.33
H_2_CO*•••* **Delt–O** ^e^	−6.53	H_2_CO*•••* **Delt–S** ^e^	−6.93
H_2_CO*•••* **Squar–O** ^e^	−8.51	H_2_CO*•••* **Squar–S** ^e^	−9.24

^a^ Geometries computes at CCSD(T)/aVTZ for the neutral H_m_X•••H_m_Y, cationic H_m + 1_X^+^•••H_m_Y, and anionic H_m_X•••H_m–1_Y^−^ complexes, CCSD(T)/jul‐VTZ for H_2_CO*•••* **Am–X** and H_2_CO*•••* **Ur–X** (X = O, S), and CCSD(T)/jul‐VDZ for H_2_CO*•••* **Delt–X** and H_2_CO*•••* **Squar–X** (X = O, S).

^b^ See Supporting Information Tables S9–S44 for complete focal‐point analysis.

^c^ Hydrogen‐bond energy computed as ∆*E* = ∆*E*_strain_ + ∆*E*_int_^CP^, see Supporting Information Table S45 for all energies.

^d^ Geometry could not be located.

^e^ Computed using *C*_2v_ symmetry to mimic supramolecular motifs.

For the neutral H_m_X•••H_m_Y, the *∆E* bond energies are strongest among second‐period element‐containing complexes. The *∆E* becomes less favorable descending a group, with the largest decrease in magnitude occurring between the second‐ and third‐period analogues. The length of the hydrogen bond inversely correlates with the bond energy, with the bond length increasing by at least 0.5 Å between the second‐ and third‐period analogues and increasing slightly (about +0.14 Å) between the third‐ and fourth‐period. The cationic H_m + 1_X^+^•••H_m_Y have much larger bond energies than their neutral counterparts (see also Ref. [151, 152, 153]). The introduction of charged species also reveals that the strain energy contributes significantly to the overall bond energy for these cations, especially the group 17 complexes (12.32, 43.74, and 52.73 kcal mol^−1^ for H_2_F^+^•••HY, Y = F, Cl, and Br, respectively, Table S45). The stronger interaction energy and greater strain energy are complementary effects [27, 28, 29, 30, 31, 32]; the deformation of the monomers creates a more favorable interaction, and the increased interaction is responsible for a greater deformation (Table S45). Although the group 15 (X = N, Y = N, P, and As) and 16 (X = O, Y = O, S, and Se) complexes follow the same decreasing trend for the bond energies as the neutral species, the group 17 (X = F, Y = F, Cl, and Br) cationic complexes display a stronger bond energy as substitution occurs. The stronger bond energies in H_2_F^+^•••HY can be elucidated via the hydrogen‐bond distances with H_4_N^+^•••H_3_Y and H_3_O^+^•••H_2_Y significantly increasing in length moving down the group (1.606, 2.288, and 2.387 Å for H_4_N^+^•••H_3_Y, Y = N, P, and As, and 1.197, 1.811, and 1.959 Å for H_3_O^+^•••H_2_Y, Y = O, S, and Se) while H_2_F^+^•••HY has much smaller changes in the bond (1.145, 1.378, and 1.497 Å for H_2_F^+^•••HY, Y = F, (Cl, and Br). The presence of a charged monomer significantly increases bond energies compared to the neutral species, with cationic species generally being stronger, except in the case of HF•••F^−^ (−44.04 kcal mol^−1^) versus H_2_F^+^•••HF (−32.74 kcal mol^−1^). The strain energy is smaller than in the cationic species but not negligible like in the neutral species. Similar to their neutral counterparts, the bond energy weakens with substitution down the group.

For the larger hydrogen‐bonded complexes, the H_2_CO•••Am–X and H_2_CO•••Ur–X complexes (X = O, S) were computed with *C*_1_ symmetry and are minimal structures on the potential energy surface. Unlike the H_2_O•••H_2_Y, cationic H_3_O^+^•••H_2_Y, and anionic H_2_O•••HY^−^ complexes (Y = O, S, Se) from earlier, substitution down the group for the supramolecular species exhibits stronger bond energies. This effect was explained by Fonseca Guerra and coworkers, where they utilized DFT and the ASM to show that the effective steric size of the chalcogen atom is what decides the strength of the hydrogen‐bond [40, 41]. Sulfur elongates the double bond on the amide species allowing a greater flow of electrons toward the sulfur and making the amide group more susceptible to hydrogen bonding. Although the *C*_1_ structures are proper minima on the potential energy surface, the planar, *C*_2v_ counterparts are more comparable to the full structures from which the chosen motifs are taken. For the large H_2_CO•••Delt–X and H_2_CO•••Squar–X complexes, only *C*_2v_ structures were computed. As the size of the planar monomer increases, the bond energies become more favorable, with the sulfur‐substituted species having a greater bond energy than the oxygen‐substituted ones. The cause for a stronger bond energy with increasing motif size can be inferred from de Azevedo Santos and coworkers [42]. In their study, they examined linear polymers of hydrogen‐bonded urea, deltamide, and squaramide species. They show that, when a hydrogen bond is introduced, the covalent component in the σ system and the π polarization increase with increasing motif size, although the π polarization does so to a lesser extent. It is notable that in H_2_CO•••Delt–X the bond energy has little variation with substitution (−6.53 and −6.93 kcal mol^−1^ for X = O and S, respectively). The small change is also observed in the hydrogen‐bond distance with H_2_CO•••Delt–X being the only species of the larger complexes where the bond length increases (+0.04 Å) with substitution. Using the ASM to decompose the bond energies of the bifurcated hydrogen‐bonded motifs (H_2_CO•••Ur–X, H_2_CO•••Delt–X, and H_2_CO•••Squar–X) of this study reveals that these species have very small strain energies (Table S45). Consequently, the interaction energy is a reasonable estimate (about 98% contribution) to the total bond energy (Table S45).

### 3.2 Performance of DFT Methods for Hydrogen Bonding Energetics

**FIGURE 1:**
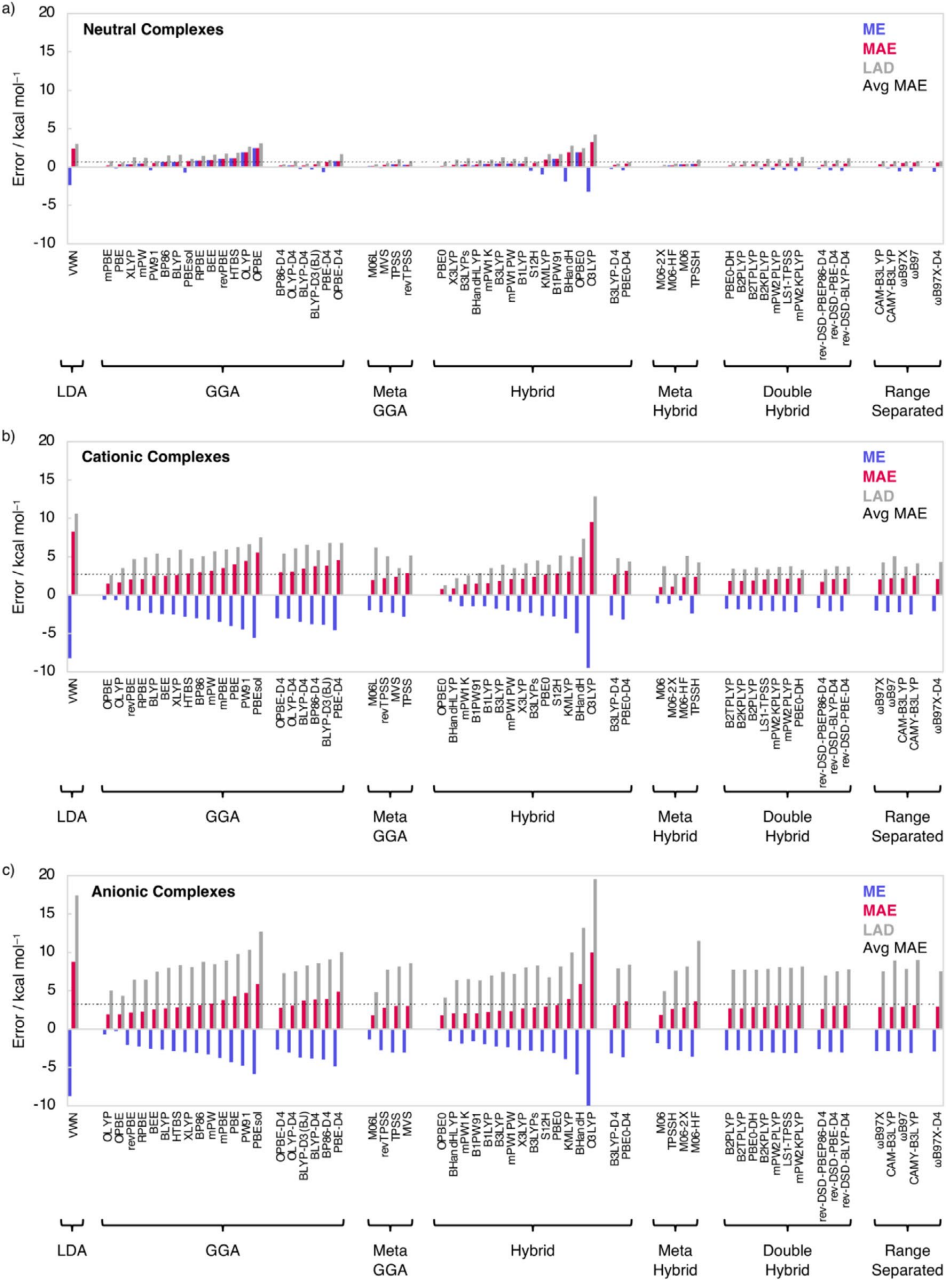
Mean error (ME), mean absolute error (MAE), and largest absolute deviation (LAD) for the hydrogen bond energies of (a) the neutral H_m_X•••H_m_Y; (b) the cationic H_m + 1_X^+^•••H_m_Y; and (c) anionic H_m_X•••H_m–1_Y^−^ complexes, computed using various density functional approximations at ZORA‐DFT/TZ2P//CCSD(T)/aVTZ compared to FPA methods targeting CCSDT(Q)/CBS//CCSD(T)/aVTZ. DFT functionals are ordered based on their performance per class.

Next, we examine the counterpoise uncorrected hydrogen‐bond energies (∆*E*) of the neutral H_m_X•••H_m_Y, cationic H_m_X•••H_m + 1_Y^+^, and anionic H_m–1_X^−^•••H_m_Y complexes (X = N, O, F; Y = N, P, As, O, S, Se, F, Cl, Br; m = 3, 2, 1) using the 60 DFT specified in the Computational Methods section in conjunction with the TZ2P STO BS and the ZORA to treat relativistic effects atop the CCSD(T)/aVTZ geometries, denoted as ZORA‐DFT/TZ2P//CCSD(T)/aVTZ. The performance of the DFT is assessed by comparing the computed hydrogen bonding energetics with our accurate CCSDT(Q)/CBS benchmark values (Table 1). Figure 1 shows, for all 60 functionals, the mean error (ME), mean absolute error (MAE), and largest absolute deviation (LAD) of the hydrogen bond energy relative to the FPA CCSDT(Q)/CBS targeted energy values for all systems. Note that the numerical data of our statistical analyses, as well as the counterpoise corrected hydrogen‐bond energies (∆*E*_CPC_) can be found in Supporting Information Tables S46–S51.

Starting with the neutral H_m_X•••H_m_Y complexes, the best agreement with our ab initio benchmark hydrogen‐bond energies is obtained by functionals of the dispersion‐corrected GGA and meta‐hybrid families, with MAEs of 0.1 (M06‐2X) and 0.2 kcal mol^−1^ (BP86‐D4 and OLYP‐D4). Among these best performing DFT functionals, the LADs are also the smallest for M06‐2X and BP86‐D4, namely, only 0.3 kcal mol^−1^. For OLYP‐D4, on the other hand, the LAD is larger; that is, 0.8 kcal mol^−1^. Including CPC slightly affects absolute errors but does not alter the ranking (average BSSE is 0.3 kcal mol^−1^): accurate DFT functionals remain accurate, poor ones remain poor (Table S46). We want to explicitly highlight that the popular dispersion‐corrected DFT approach BLYP‐D3(BJ) [40, 41, 42], as well as the improved BLYP‐D4, are able to describe hydrogen bonds with satisfactory accuracy, with an MAE of around 0.3 kcal mol^−1^. This is in line with other benchmark studies involving a series of different hydrogen‐bonded complexes [38, 39].

Next, we move to the cationic H_m_X•••H_m + 1_Y^+^ and anionic H_m–1_X^−^•••H_m_Y complexes, for which it becomes evident that the errors in the hydrogen‐bond energies are significantly larger than for the neutral H_m_X•••H_m_Y complexes (Figure 1b,c). The top three best DFT functionals for accurately describing the cationic H_m_X•••H_m + 1_Y^+^ complexes are the hybrid and meta‐hybrid functionals OPBE0, BHandHLYP, M06, and M06‐2X, with MAEs of 0.8, 0.9, 1.1, and 1.1 kcal mol^−1^. The best DFT functionals for evaluating the anionic hydrogen bonds in H_m–1_X^−^•••H_m_Y complexes, on the other hand, are the meta‐GGA functional M06L, hybrid functional OPBE0, and hybrid functional M06, all with a MAE of 1.8 kcal mol^−1^. Interestingly, the GGAs OPBE and OLYP are, although not ranked among the top three DFT functionals, able to relatively accurately describe the strength of charged hydrogen bonds, having an MAE between 1.5 and 1.9 kcal mol^−1^. Furthermore, adding a dispersion correction generally results in a worsening of the description of the hydrogen bond in charged complexes, as it leads to an overestimation of the hydrogen bond strength.

**FIGURE 2:**
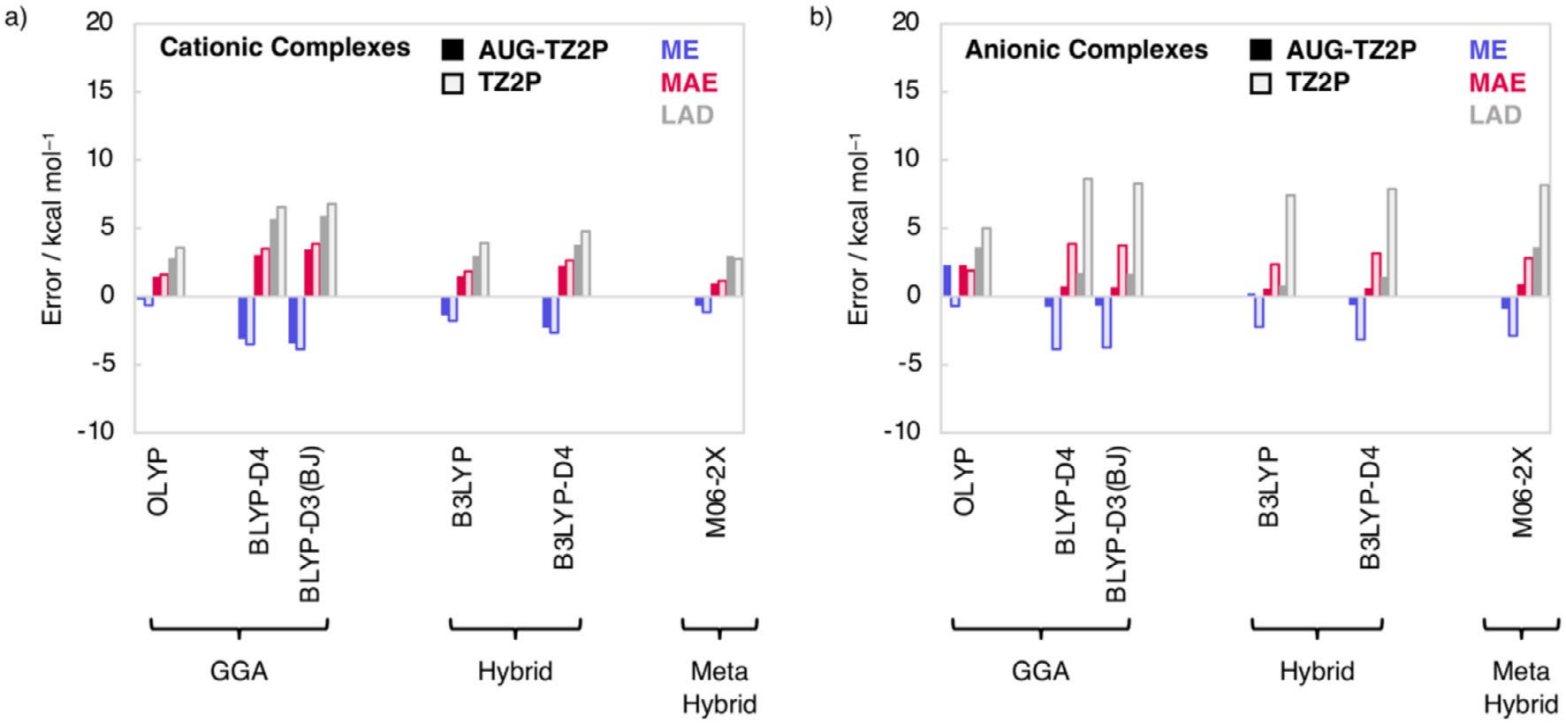
Mean error (ME), mean absolute error (MAE), and largest absolute deviation (LAD) for the hydrogen bond energies computed at ZORA‐DFT/AUG‐TZ2P and ZORA‐DFT/TZ2P of (a) the cationic H_m + 1_X^+^•••H_m_Y complexes; and (b) the anionic H_m_X•••H_m–1_Y^−^ complexes relative to CCSDT(Q)/CBS//CCSD(T)/aVTZ on CCSD(T)/aVTZ equilibrium geometries. See Supporting Information Table S54 for numerical data.

The accuracy of the hydrogen‐bond energies of the charged complexes improves when including a CPC, albeit for the cationic complexes to a smaller extent than for the anionic analogs (Tables S49 and S51). For example, for the cationic complexes the MAE of M06 decreases from 1.1 kcal mol^−1^ without CPC to 0.7 kcal mol^−1^ with CPC (average BSSE for cationic complexes is 0.7 kcal mol^−1^), whereas for the anionic complexes the MAE decreases from 1.8 kcal mol^−1^ without CPC to 0.8 kcal mol^−1^ with CPC (average BSSE for anionic complexes is 1.9 kcal mol^−1^). Nevertheless, counterpoise‐uncorrected computations can still yield acceptably accurate results for cost‐sensitive applications. Additionally, we have assessed the effect of diffuse functions on the performance of computing the hydrogen bond energy in charged systems by using the AUG‐TZ2P STO BS at ZORA‐DFT/AUG‐TZ2P level (Figure 2). Adding diffuse functions to the BS leads to a significant improvement in describing the hydrogen bond strength in the anionic complexes, decreasing the MAE by up to a few kcal mol^−1^. For example, the MAE of BLYP‐D4 decreases from 3.8 kcal mol^−1^ without diffuse functions to only 0.8 kcal mol^−1^ with diffuse functions. This occurs because, only by adding diffuse functions, the charge density can expand and mitigate the strong electron–electron repulsion in the net negatively charged systems. This effect is stronger in the more compact anionic hydrogen‐bond accepting fragments than in the anionic hydrogen‐bonded complexes. Consequently, without the additional diffuse functions, the hydrogen‐bond energies are overestimated and MAEs are larger whereas inclusion of the diffuse functions reduces this error and causes hydrogen‐bond energies to adopt the more correct, weaker values and thus smaller MAEs values [154]. The effect of adding diffuse functions is, as expected, relatively small for the cationic complexes, leading to a reduction in MAE by only a few tenths of a kcal mol^−1^ (see Figure 2).

**FIGURE 3:**
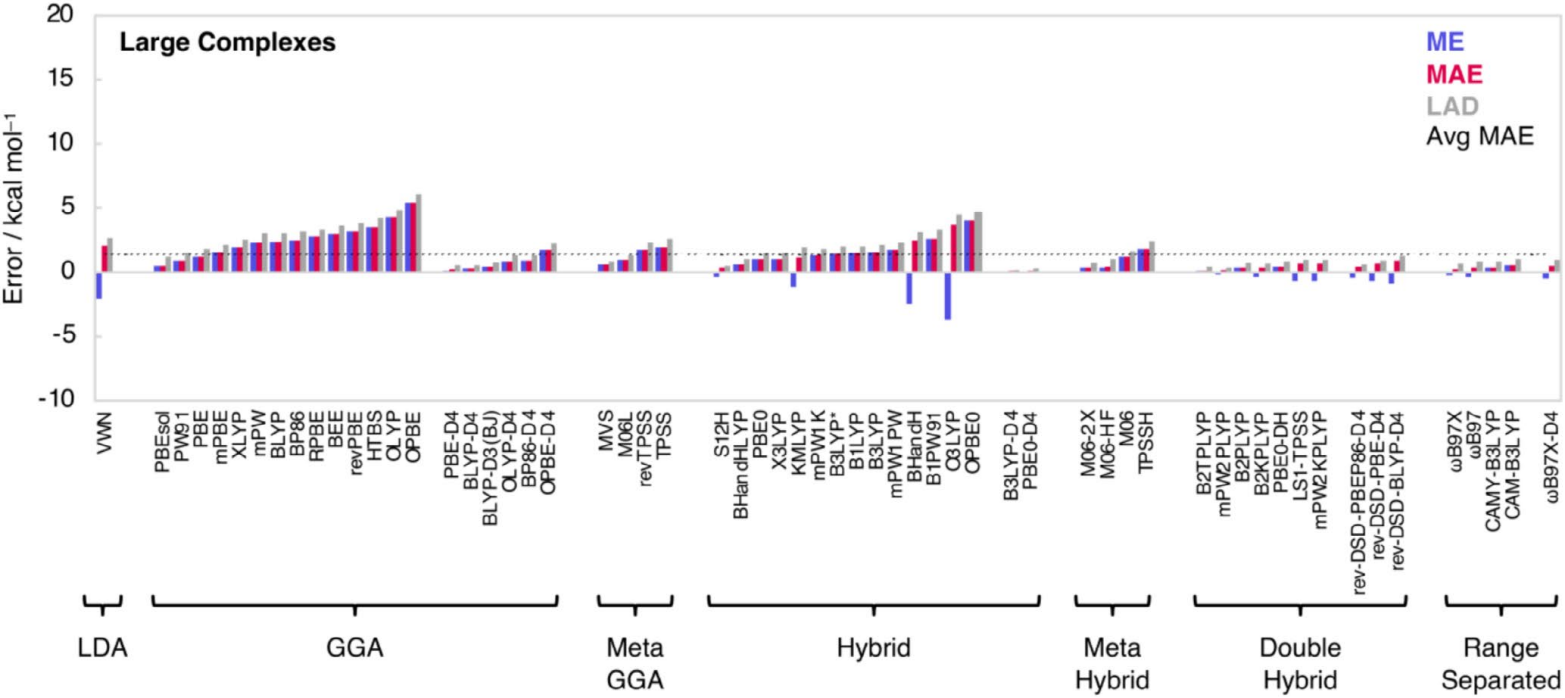
Mean error (ME), mean absolute error (MAE), and largest absolute deviation (LAD) for the hydrogen bond energies of the large complexes, computed using various density functional approximations at ZORA‐DFT/TZ2P//CCSD(T)/jul‐VTZ for H_2_CO*•••* **Am–X** and H_2_CO*•••* **Ur–X** (X = O, S), and ZORA‐DFT/TZ2P//CCSD(T)/jul‐VDZ for H_2_CO*•••* **Delt–X** and H_2_CO*•••* **Squar–X** (X = O, S) compared to FPA methods targeting CCSD(T)/CBS//CCSD(T)/aVTZ and CCSD(T)/CBS//CCSD(T)/aVDZ, respectively. DFT functionals are ordered based on their performance per class. See Supporting Information Tables S52 and S53 for numerical data.

Finally, we compute the hydrogen‐bond energies of the large complexes H_2_CO*•••* **Am–X**, H_2_CO*•••* **Ur–X**, H_2_CO*•••* **Delt–X**, and H_2_CO*•••* **Squar–X** (X = O, S) at ZORA‐DFT/TZ2P level atop CCSD(T)/jul‐VTZ geometries for H_2_CO*•••* **Am–X** and H_2_CO*•••* **Ur–X** and CCSD(T)/jul‐VDZ geometries for H_2_CO*•••* **Delt–X** and H_2_CO*•••* **Squar–X** (Figure 3). The accuracy of the various DFT functionals is again assessed by comparing the computed hydrogen‐bond energies with our accurate CCSD(T)/CBS benchmark values (Table 1). The best performing functionals are the dispersion‐corrected hybrid functionals B3LYP‐D4 and PBE0‐D4 both having a MAE of 0.1 kcal mol^−1^ and the double‐hybrid functionals B2TPLYP and mPW2PLYP, with MAEs of 0.1 and 0.2 kcal mol^−1^, respectively. Although the double‐hybrid functionals can accurately describe the hydrogen bond energies, a significant disadvantage of these functionals is that currently, in the context of a STO BS approach, they cannot be used for geometry optimizations. Including CPC again has only a minor effect as demonstrated by the average BSSE of only 0.4 kcal mol^−1^. BLYP‐D3(BJ) and BLYP‐D4 again show robust performance, yielding MAEs below 0.4 kcal mol^−1^, confirming their suitability as cost‐effective alternatives for large hydrogen‐bonded systems.

### 3.3 Performance of Selected DFT Functionals for Hydrogen Bonding Geometries

**FIGURE 4:**
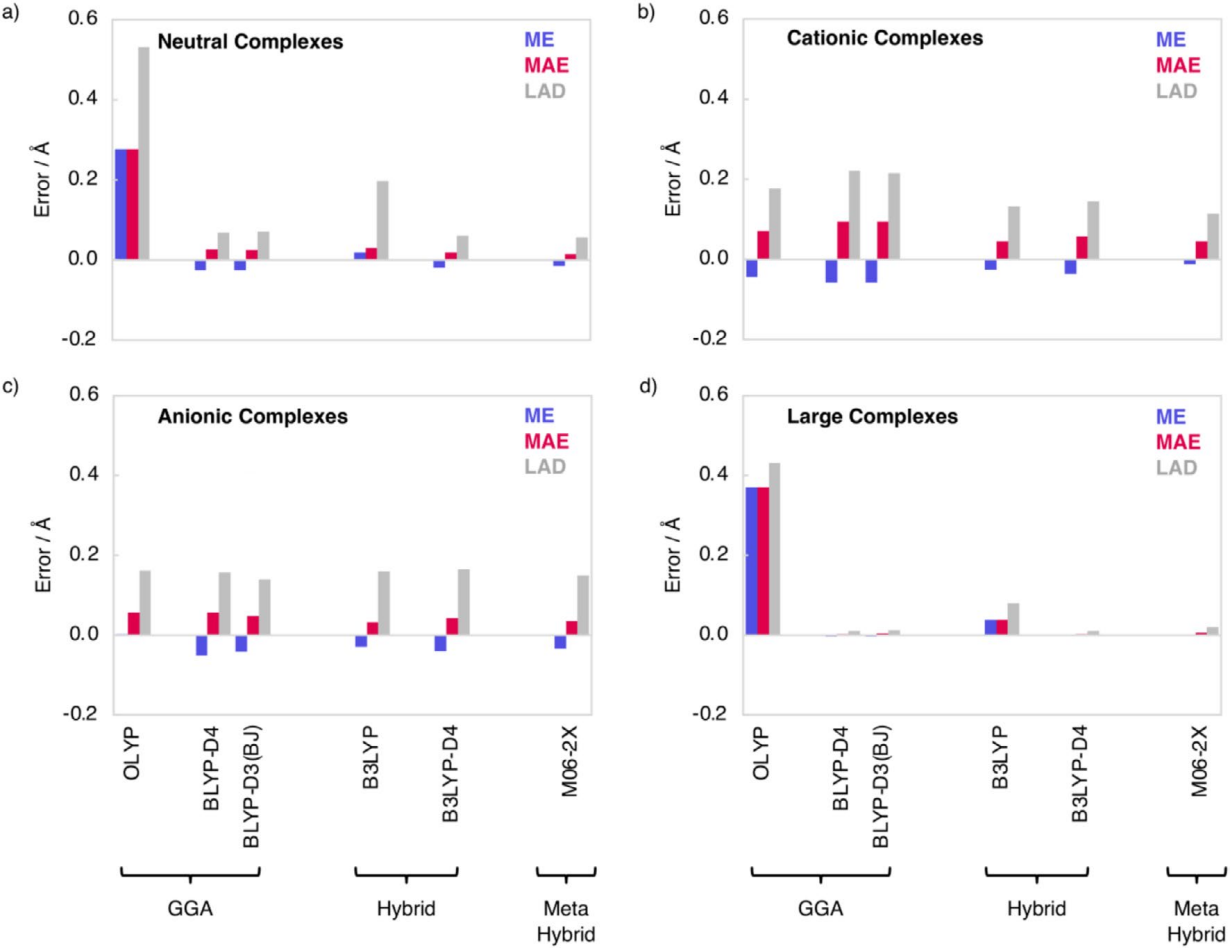
Mean error (ME), mean absolute error (MAE), and largest absolute deviation (LAD) for the hydrogen bond length of equilibrium geometries computed at ZORA‐[DFT]/TZ2P of (a) the neutral H_m_X•••H_m_Y complexes; (b) the cationic H_m + 1_X^+^•••H_m_Y complexes; (c) the anionic H_m_X•••H_m–1_Y^−^complexes relative to CCSD(T)/aVTZ; and (d) the large complexes H_2_CO*•••* **Am–X** and H_2_CO*•••* **Ur–X** (X = O, S) relative to CCSD(T)/aVTZ and for H_2_CO*•••* **Delt–X** and H_2_CO*•••* **Squar–X** (X = O, S) relative to CCSD(T)/aVDZ.

In this section, the geometries of the neutral H_m_X•••H_m_Y, cationic H_m_X•••H_m + 1_Y^+^, anionic H_m–1_X^−^•••H_m_Y (X = N, O, F; Y = N, P, As, O, S, Se, F, Cl, Br; m = 3, 2, 1), and large complexes H_2_CO*•••* **Am–X**, H_2_CO*•••* **Ur–X**, H_2_CO*•••* **Delt–X**, and H_2_CO*•••* **Squar–X** (X = O, S) are fully optimized using the following DFT at ZORA‐[DFT]/TZ2P level, where DFT = OLYP, BLYP‐D3(BJ), BLYP‐D4, B3LYP, B3LYP‐D4, and M06‐2X. Cartesian coordinates of these DFT geometries are provided in the Supporting Information. To assess the quality of the DFT structures, they are compared to the CCSD(T) benchmark geometries using the ME, MAE, and LAD of the hydrogen bond length *r*_X•••H_ (see Figure 4 and Table S55), as well as the Cartesian root‐mean‐square deviation (RMSD) analysis of the complete hydrogen‐bonded complex (see Table S55).

For all complexes, we find that, in general, all DFT functionals, except for OLYP, give reasonably accurate hydrogen bond lengths with an average MAE ranging from 0.085 to 0.114 Å. The most accurate hydrogen‐bond lengths are computed with the hybrid functional M06‐2X, with a hydrogen‐bond length MAE that ranges from only 0.005 Å for the large complexes to 0.045 Å for the cationic complexes. Interestingly, OLYP performs poorly for the neutral and large complexes, but actually yields rather good hydrogen‐bond lengths for the cationic and anionic complexes. The popular and less expensive BLYP‐D3(BJ) and BLYP‐D4 functionals give not only relatively accurate energies (*vide supra*), but also good hydrogen bond lengths, with MAEs ranging from 0.003 to 0.094 Å. The RMSD, which is a measure of the similarity between the overall structure of the hydrogen‐bonded complex computed at DFT and CCSD(T) level, reveals that all DFT functionals give accurate overall geometries with an average error in the RMSD that ranges from 0.031 Å for M06‐2X to 0.044 Å for B3LYP (Table S55). The only exception is OLYP, which demonstrates a large average error in the RMSD of 0.114 Å, similar to its lesser performance for the hydrogen‐bond length.

**FIGURE 5:**
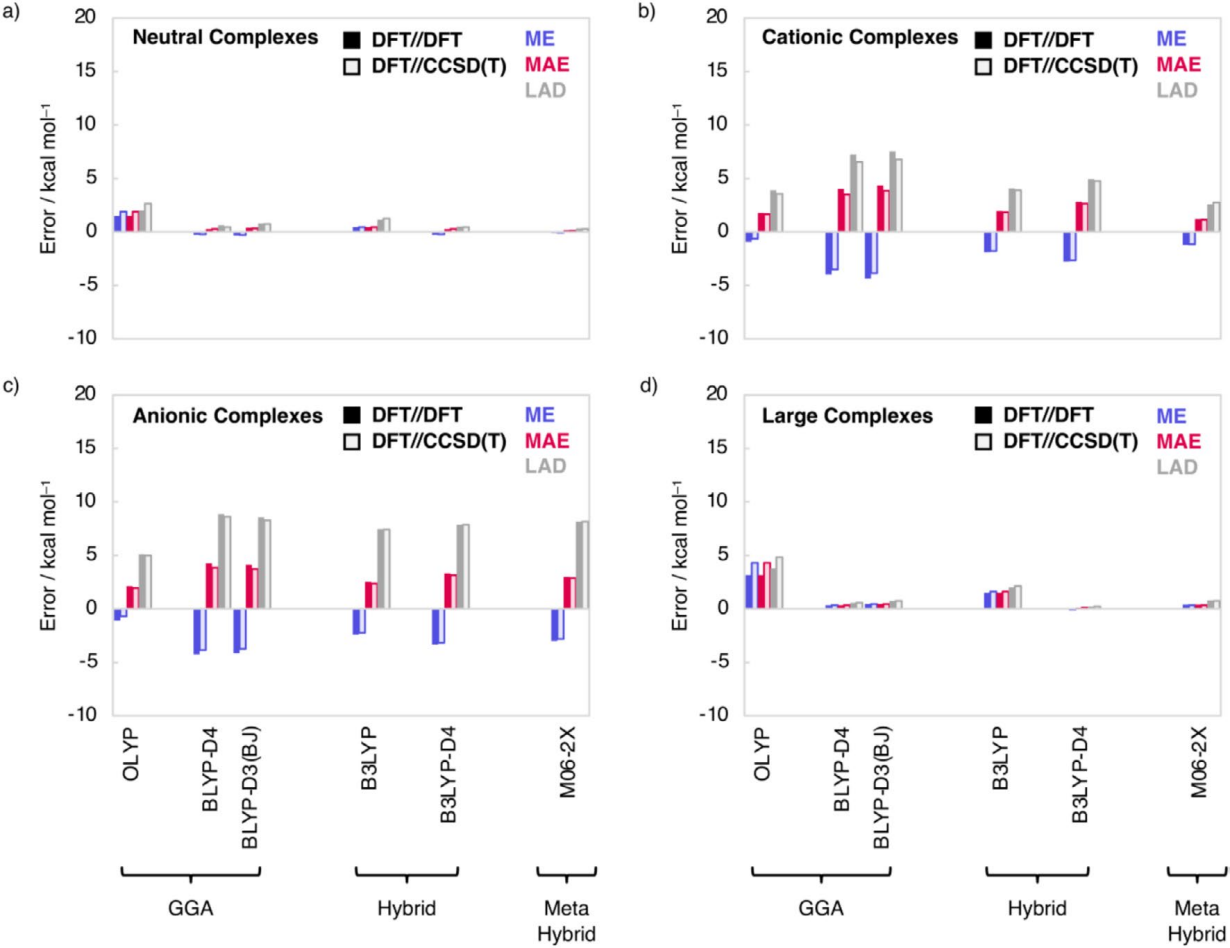
Mean error (ME), mean absolute error (MAE), and largest absolute deviation (LAD) for the hydrogen bond energies computed at ZORA‐DFT/TZ2P of (a) the neutral H_m_X•••H_m_Y complexes; (b) the cationic H_m + 1_X^+^•••H_m_Y complexes; (c) the anionic H_m_X•••H_m–1_Y^−^complexes relative to CCSDT(Q)/CBS//CCSD(T)/aVTZ; and (d) the large complexes H_2_CO*•••* **Am–X** and H_2_CO*•••* **Ur–X** (X = O, S) relative to CCSD(T)/CBS//CCSD(T)/aVTZ and for H_2_CO*•••* **Delt–X** and H_2_CO*•••* **Squar–X** (X = O, S) relative to CCSD(T)/CBS//CCSD(T)/aVDZ using equilibrium geometries computed at CCSD(T) and DFT level.

Next, we compute the hydrogen‐bond energies of these DFT geometries at ZORA‐DFT/TZ2P level and evaluate their accuracy relative to our accurate CCSDT(Q)/CBS benchmark values atop CCSD(T) geometries (Figure 5). We find that for the neutral and large complexes, the performance in hydrogen‐bond energy determination of the DFT functionals increases when going from CCSD(T) to DFT geometries. In contrast, for the cationic and anionic complexes, the description of the hydrogen‐bond energy becomes worse going from CCSD(T) to DFT geometries. Looking at the performance of the individual DFT functionals, we see that M06‐2X gives, for both DFT and CCSD(T) geometries, the most accurate hydrogen‐bond energies, with MAE ranging from 0.1 to 3.0 kcal mol^−1^. The less expensive BLYP‐D3(BJ) and BLYP‐D4(BJ) remain to perform well, having MAEs ranging from 0.3 to 4.2 kcal mol^−1^. The largest errors in hydrogen‐bond energies are found for the cationic and anionic complexes, in the latter case due to the lack of diffuse functions. As mentioned before, OLYP performs reasonably well for the charged systems, with a MAE of 1.8 kcal mol^−1^ for the cationic complexes and 2.1 kcal mol^−1^ for the anionic complexes. Overall, the use of M06‐2X results in accurate hydrogen bonding geometries and energies. However, the significantly more cost‐efficient BLYP‐D3(BJ) and BLYP‐D4 functionals closely follow in accuracy, especially for the neutral and large complexes.

## 4 Conclusions

In this work, we have carried out a comprehensive ab initio benchmark study to establish highly accurate reference data for hydrogen‐bonded complexes. Our dataset encompasses the neutral H_m_X•••H_m_Y, cationic H_m + 1_X^+^•••H_m_Y, and anionic H_m_X•••H_m–1_Y^−^ complexes (X = N, O, F; Y = N, P, As, O, S, Se, F, Cl, Br; m = 3, 2, 1) and the large complexes involving chalcoamides (**Am–X**), chalcoureas (**Ur–X**), chalcodeltamides (**Delt–X**), and chalocosquaramides (**Squar–X**) with formaldehyde (H_2_CO) (X = O, S). Our reference hydrogen‐bond energies are derived from a hierarchical series of high‐level ab initio quantum chemical methods, reaching CCSDT(Q) for the small neutral, cationic, and anionic complexes and CCSD(T) for the larger systems, combined with systematic BS extrapolation to the CBS limit. This protocol ensures convergence of interaction energies to within a few tenths of a kcal mol^−1^.

We assessed the performance of 60 DFT (including 12 dispersion‐corrected) with a TZ2P STO BS and the ZORA to account for relativistic effects. Among these, the meta‐hybrid functional M06‐2X stands out as best overall performer. A more detailed analysis shows distinct performance trends depending on the nature of the hydrogen‐bonding complex: (i) M06‐2X performs very well for the neutral complexes (MAE = 0.1 kcal mol^−1^), (ii) OPBE0 is optimal for cationic complexes (MAE = 0.8 kcal mol^−1^), (iii) M06‐L, OPBE0, and M06 deliver comparable accuracy for the anionic complexes (MAE = 1.8 kcal mol^−1^), and (iv) the dispersion‐corrected hybrid functionals B3LYP‐D4 and PBE0‐D4 perform best for the large complexes, each achieving a MAE of 0.1 kcal mol^−1^. For the charged complexes, the inclusion of diffuse functions and CPC is found to improve the agreement with the benchmark data further.

Looking at geometrical accuracy, M06‐2X is again the best performing DFT with a MAE in hydrogen‐bond lengths of only 0.005 Å for the large complexes and 0.045 Å for the cationic systems. The dispersion‐corrected GGA BLYP‐D3(BJ) and BLYP‐D4 also prove to be valuable alternatives, as they deliver not only accurate hydrogen‐bond energies but also reliable geometries, making them an attractive, cost‐effective choice for studying large and complex hydrogen‐bonded systems. This benchmark establishes one of the most accurate and diverse datasets while providing clear guidance for selecting reliable density functionals across a variety of hydrogen‐bonded complexes.

## Conflicts of Interest

The authors declare no conflicts of interest.

## Supporting information


**Data S1:** jcc70265‐sup‐0001‐SupinfoA.pdf.

**Data S2:** jcc70265‐sup‐0002‐SupinfoB.xlsx.

**Data S3:** jcc70265‐sup‐0003‐SupinfoC.zip.

## Data Availability

The data that supports the findings of this study are available in the Supporting Information of this article.
