# Supplementary material for: Hydrogen Bond Benchmark: Focal‐Point Analysis and Assessment of DFT Functionals
Source: J Comput Chem. 2025 Nov 7;46(30):e70265. doi: 10.1002/jcc.70265 (PMC12595407; doi:10.1002/jcc.70265)
Supplement: Supplementary file 1 — Data S1: jcc70265‐sup‐0001‐SupinfoA.pdf. [file JCC-46-0-s002.pdf]

Content

Table S1. Harmonic vibrational frequencies (in cm^{-1}) of the fragments of the neutral H_mX•••H_mY, cationic $H_{m+1}X^+$•••H_mY, and anionic H_mX•••$H_{m-1}Y^-$ complexes, computed at CCSD(T)/aVTZ.

Table S2. Harmonic vibrational frequencies (in cm^{-1}) of the neutral H_mX•••H_mY complexes, computed at CCSD(T)/aVTZ.

Table S3. Harmonic vibrational frequencies (in cm^{-1}) of the cationic $H_{m+1}X^+$•••H_mY complexes, computed at CCSD(T)/aVTZ.

Table S4. Harmonic vibrational frequencies (in cm^{-1}) of the anionic H_mX•••$H_{m-1}Y^-$complexes, computed at CCSD(T)/aVTZ.

Table S5. Harmonic vibrational frequencies (in cm^{-1}) of the H_2CO•••**Am–X** complexes, computed at CCSD(T)/jul-VTZ.

Table S6. Harmonic vibrational frequencies (in cm^{-1}) of the H_2CO•••**Ur–X** complexes, computed at CCSD(T)/jul-VTZ.

Table S7. Harmonic vibrational frequencies (in cm^{-1}) of the H_2CO•••**Delt–X** complexes, computed at CCSD(T)/jul-VDZ.

Table S8. Harmonic vibrational frequencies (in cm^{-1}) of the H_2CO•••**Squar–X** complexes, computed at CCSD(T)/jul-VDZ.

Table S9. Focal point analysis for the NH_3•••NH_3 complex with further additive corrections at the CCSD(T) level of theory with units in kcal mol^{-1}. Bracketed values indicate the extrapolated energies or additive corrections. Additive corrections are indicated by the δ which represents the incremental change from the previous energy.

Table S10. Focal point analysis for the NH_3•••PH_3 complex with further additive corrections at the CCSD(T) level of theory with units in kcal mol^{-1}. Bracketed values indicate the extrapolated energies or additive corrections. Additive corrections are indicated by the δ which represents the incremental change from the previous energy.

Table S11. Focal point analysis for the H_2O•••H_2O complex with further additive corrections at the CCSD(T) level of theory with units in kcal mol^{-1}. Bracketed values indicate the extrapolated energies or additive corrections. Additive corrections are indicated by the δ which represents the incremental change from the previous energy.

Table S12. Focal point analysis for the H_2O•••H_2S complex with further additive corrections at the CCSD(T) level of theory with units in kcal mol^{-1}. Bracketed values indicate the extrapolated energies or additive corrections. Additive corrections are indicated by the δ which represents the incremental change from the previous energy.

Table S13. Focal point analysis for the H_2O•••H_2Se complex with further additive corrections at the CCSD(T) level of theory with units in kcal mol^{-1}. Bracketed values indicate the

extrapolated energies or additive corrections. Additive corrections are indicated by the δ which represents the incremental change from the previous energy.

Table S14. Focal point analysis for the HF•••HF complex with further additive corrections at the CCSD(T) level of theory with units in kcal mol^{-1}. Bracketed values indicate the extrapolated energies or additive corrections. Additive corrections are indicated by the δ which represents the incremental change from the previous energy.

Table S15. Focal point analysis for the HF•••HCl complex with further additive corrections at the CCSD(T) level of theory with units in kcal mol^{-1}. Bracketed values indicate the extrapolated energies or additive corrections. Additive corrections are indicated by the δ which represents the incremental change from the previous energy.

Table S16. Focal point analysis for the HF•••HBr complex with further additive corrections at the CCSD(T) level of theory with units in kcal mol^{-1}. Bracketed values indicate the extrapolated energies or additive corrections. Additive corrections are indicated by the δ which represents the incremental change from the previous energy.

Table S17. Focal point analysis for the NH_4^+•••NH_3 complex with further additive corrections at the CCSD(T) level of theory with units in kcal mol^{-1}. Bracketed values indicate the extrapolated energies or additive corrections. Additive corrections are indicated by the δ which represents the incremental change from the previous energy.

Table S18. Focal point analysis for the NH_4^+•••PH_3 complex with further additive corrections at the CCSD(T) level of theory with units in kcal mol^{-1}. Bracketed values indicate the extrapolated energies or additive corrections. Additive corrections are indicated by the δ which represents the incremental change from the previous energy.

Table S19. Focal point analysis for the NH_4^+•••AsH_3 complex with further additive corrections at the CCSD(T) level of theory with units in kcal mol^{-1}. Bracketed values indicate the extrapolated energies or additive corrections. Additive corrections are indicated by the δ which represents the incremental change from the previous energy.

Table S20. Focal point analysis for the H_3O^+•••H_2O complex with further additive corrections at the CCSD(T) level of theory with units in kcal mol^{-1}. Bracketed values indicate the extrapolated energies or additive corrections. Additive corrections are indicated by the δ which represents the incremental change from the previous energy.

Table S21. Focal point analysis for the H_3O^+•••H_2S complex with further additive corrections at the CCSD(T) level of theory with units in kcal mol^{-1}. Bracketed values indicate the extrapolated energies or additive corrections. Additive corrections are indicated by the δ which represents the incremental change from the previous energy.

Table S22. Focal point analysis for the H_3O^+•••H_2Se complex with further additive corrections at the CCSD(T) level of theory with units in kcal mol^{-1}. Bracketed values indicate the extrapolated energies or additive corrections. Additive corrections are indicated by the δ which represents the incremental change from the previous energy.

Table S23. Focal point analysis for the H_2F^+•••HF complex with further additive corrections at the CCSD(T) level of theory with units in kcal mol^{-1}. Bracketed values indicate the extrapolated energies or additive corrections. Additive corrections are indicated by the δ which represents the incremental change from the previous energy.

Table S24. Focal point analysis for the H_2F^+•••HCl complex with further additive corrections at the CCSD(T) level of theory with units in kcal mol^{-1}. Bracketed values indicate the extrapolated energies or additive corrections. Additive corrections are indicated by the δ which represents the incremental change from the previous energy.

Table S25. Focal point analysis for the H_2F^+•••HBr complex with further additive corrections at the CCSD(T) level of theory with units in kcal mol^{-1}. Bracketed values indicate the extrapolated energies or additive corrections. Additive corrections are indicated by the δ which represents the incremental change from the previous energy.

Table S26. Focal point analysis for the NH_3•••NH_2^-complex with further additive corrections at the CCSD(T) level of theory with units in kcal mol^{-1}. Bracketed values indicate the extrapolated energies or additive corrections. Additive corrections are indicated by the δ which represents the incremental change from the previous energy.

Table S27. Focal point analysis for the NH_3•••PH_2^-complex with further additive corrections at the CCSD(T) level of theory with units in kcal mol^{-1}. Bracketed values indicate the extrapolated energies or additive corrections. Additive corrections are indicated by the δ which represents the incremental change from the previous energy.

Table S28. Focal point analysis for the NH_3•••AsH_2^-complex with further additive corrections at the CCSD(T) level of theory with units in kcal mol^{-1}. Bracketed values indicate the extrapolated energies or additive corrections. Additive corrections are indicated by the δ which represents the incremental change from the previous energy.

Table S29. Focal point analysis for the H_2O•••OH^- complex with further additive corrections at the CCSD(T) level of theory with units in kcal mol^{-1}. Bracketed values indicate the extrapolated energies or additive corrections. Additive corrections are indicated by the δ which represents the incremental change from the previous energy.

Table S30. Focal point analysis for the H_2O•••SH^- complex with further additive corrections at the CCSD(T) level of theory with units in kcal mol^{-1}. Bracketed values indicate the extrapolated energies or additive corrections. Additive corrections are indicated by the δ which represents the incremental change from the previous energy.

Table S31. Focal point analysis for the H_2O•••SeH^- complex with further additive corrections at the CCSD(T) level of theory with units in kcal mol^{-1}. Bracketed values indicate the extrapolated energies or additive corrections. Additive corrections are indicated by the δ which represents the incremental change from the previous energy.

Table S32. Focal point analysis for the HF•••F^- complex with further additive corrections at the CCSD(T) level of theory with units in kcal mol^{-1}. Bracketed values indicate the extrapolated energies or additive corrections. Additive corrections are indicated by the δ which represents the incremental change from the previous energy.

Table S33. Focal point analysis for the HF•••Cl^- complex with further additive corrections at the CCSD(T) level of theory with units in kcal mol^{-1}. Bracketed values indicate the extrapolated energies or additive corrections. Additive corrections are indicated by the δ which represents the incremental change from the previous energy.

Table S34. Focal point analysis for the HF•••Br^- complex with further additive corrections at the CCSD(T) level of theory with units in kcal mol^{-1}. Bracketed values indicate the extrapolated energies or additive corrections. Additive corrections are indicated by the δ which represents the incremental change from the previous energy.

Table S35. Focal point analysis for the H_2CO•••**Am–O** complex with further additive corrections at the CCSD(T) level of theory with units in kcal mol^{-1}. Bracketed values indicate the extrapolated energies or additive corrections. Additive corrections are indicated by the δ which represents the incremental change from the previous energy.

Table S36. Focal point analysis for the H_2CO•••**Am–S** complex with further additive corrections at the CCSD(T) level of theory with units in kcal mol^{-1}. Bracketed values indicate the extrapolated energies or additive corrections. Additive corrections are indicated by the δ which represents the incremental change from the previous energy.

Table S37. Focal point analysis for the H_2CO•••**Ur–O** complex in C_1 with further additive corrections at the CCSD(T) level of theory with units in kcal mol^{-1}. Bracketed values indicate the extrapolated energies or additive corrections. Additive corrections are indicated by the δ which represents the incremental change from the previous energy.

Table S38. Focal point analysis for the H_2CO•••**Ur–O** complex in C_{2v} with further additive corrections at the CCSD(T) level of theory with units in kcal mol^{-1}. Bracketed values indicate the extrapolated energies or additive corrections. Additive corrections are indicated by the δ which represents the incremental change from the previous energy.

Table S39. Focal point analysis for the H_2CO•••**Ur–S** complex in C_1 with further additive corrections at the CCSD(T) level of theory with units in kcal mol^{-1}. Bracketed values indicate the extrapolated energies or additive corrections. Additive corrections are indicated by the δ which represents the incremental change from the previous energy.

Table S40. Focal point analysis for the H_2CO•••**Ur–S** complex in C_{2v} with further additive corrections at the CCSD(T) level of theory with units in kcal mol^{-1}. Bracketed values indicate the extrapolated energies or additive corrections. Additive corrections are indicated by the δ which represents the incremental change from the previous energy.

Table S41. Focal point analysis for the H_2CO•••**Delt–O** complex with further additive corrections at the CCSD(T) level of theory with units in kcal mol^{-1}. Bracketed values indicate the extrapolated energies or additive corrections. Additive corrections are indicated by the δ which represents the incremental change from the previous energy.

Table S42. Focal point analysis for the H_2CO•••**Delt–S** complex with further additive corrections at the CCSD(T) level of theory with units in kcal mol^{-1}. Bracketed values indicate the extrapolated energies or additive corrections. Additive corrections are indicated by the δ which represents the incremental change from the previous energy.

Table S43. Focal point analysis for the H_2CO•••**Squar–O** complex with further additive corrections at the CCSD(T) level of theory with units in kcal mol^{-1}. Bracketed values indicate the extrapolated energies or additive corrections. Additive corrections are indicated by the δ which represents the incremental change from the previous energy.

Table S44. Focal point analysis for the H_2CO•••**Squar–S** complex with further additive corrections at the CCSD(T) level of theory with units in kcal mol^{-1}. Bracketed values indicate the extrapolated energies or additive corrections. Additive corrections are indicated by the δ which represents the incremental change from the previous energy.

Table S45. Final extrapolated hydrogen bond energies (ΔE), strain energies (ΔE_{strain}), and interaction energies (ΔE_{int}, in kcal mol^{-1}) targeting the CCSDT(Q)/CBS limit for the neutral H_mX•••H_mY, cationic $H_{m+1}X^+$•••H_mY, and anionic H_mX•••$H_{m-1}Y^-$ complexes and CCSD(T)/CBS limit for the large complexes, including a core-correlation correction, a relativistic correction, and the diagonal Born-Oppenheimer correction where appropriate.

Table S46. Mean error (ME), mean absolute error (MAE), and largest absolute deviation (LAD) for the hydrogen bond energies of the neutral H_mX•••H_mY complexes without counterpoise corrections, computed using various density functional approximations at ZORA-DFT/TZ2P//CCSD(T)/aVTZ compared to FPA methods targeting CCSDT(Q)/CBS//CCSD(T)/aVTZ.

Table S47. Mean error (ME), mean absolute error (MAE), and largest absolute deviation (LAD) for the hydrogen bond energies of the neutral H_mX•••H_mY complexes with counterpoise corrections, computed using various density functional approximations at ZORA-DFT/TZ2P//CCSD(T)/aVTZ compared to FPA methods targeting CCSDT(Q)/CBS//CCSD(T)/aVTZ.

Table S48. Mean error (ME), mean absolute error (MAE), and largest absolute deviation (LAD) for the hydrogen bond energies of the cationic $H_{m+1}X^+$•••H_mY complexes without counterpoise corrections, computed using various density functional approximations at ZORA-DFT/TZ2P//CCSD(T)/aVTZ compared to FPA methods targeting CCSDT(Q)/CBS//CCSD(T)/aVTZ.

Table S49. Mean error (ME), mean absolute error (MAE), and largest absolute deviation (LAD) for the hydrogen bond energies of the cationic $H_{m+1}X^+$•••H_mY complexes with counterpoise corrections, computed using various density functional approximations at ZORA-DFT/TZ2P//CCSD(T)/aVTZ compared to FPA methods targeting CCSDT(Q)/CBS//CCSD(T)/aVTZ.

Table S50. Mean error (ME), mean absolute error (MAE), and largest absolute deviation (LAD) for the hydrogen bond energies of the anionic H_mX•••$H_{m-1}Y^-$ complexes without counterpoise corrections, computed using various density functional approximations at ZORA-DFT/TZ2P//CCSD(T)/aVTZ compared to FPA methods targeting CCSDT(Q)/CBS//CCSD(T)/aVTZ.

Table S51. Mean error (ME), mean absolute error (MAE), and largest absolute deviation (LAD) for the hydrogen bond energies of the anionic $H_mX•••H_{m–1}Y^–$ complexes with counterpoise corrections, computed using various density functional approximations at ZORA-DFT/TZ2P//CCSD(T)/aVTZ compared to FPA methods targeting CCSDT(Q)/CBS//CCSD(T)/aVTZ.

Table S52. Mean error (ME), mean absolute error (MAE), and largest absolute deviation (LAD) for the hydrogen bond energies of the large complexes without counterpoise correction, computed using various density functional approximations at ZORA-DFT/TZ2P//jul-VTZ for H_2CO•••**Am–X** and H_2CO•••**Ur–X** (X = O, S), and ZORA-DFT/TZ2P//CCSD(T)/jul-VDZ for H_2CO•••**Delt–X** and H_2CO•••**Squar–X** (X = O, S) compared to FPA methods targeting CCSD(T)/CBS//CCSD(T)/aVTZ and CCSD(T)/CBS//CCSD(T)/aVDZ, respectively.

Table S53. Mean error (ME), mean absolute error (MAE), and largest absolute deviation (LAD) for the hydrogen bond energies of the large complexes with counterpoise correction, computed using various density functional approximations at ZORA-DFT/TZ2P//jul-VTZ for H_2CO•••**Am–X** and H_2CO•••**Ur–X** (X = O, S), and ZORA-DFT/TZ2P//CCSD(T)/jul-VDZ for H_2CO•••**Delt–X** and H_2CO•••**Squar–X** (X = O, S) compared to FPA methods targeting CCSD(T)/CBS//CCSD(T)/aVTZ and CCSD(T)/CBS//CCSD(T)/aVDZ, respectively.

Table S54. Mean error (ME), mean absolute error (MAE), and largest absolute deviation (LAD) for the hydrogen bond energies without and with counterpoise correction computed at ZORA-DFT/(AUG-)TZ2P of a) the cationic $H_{m+1}X^+•••H_mY$ complexes; and b) the anionic $H_mX•••H_{m–1}Y^–$complexes relative to CCSDT(Q)/CBS//CCSD(T)/aVTZ on CCSD(T)/aVTZ equilibrium geometries.

Table S55. Mean error (ME), mean absolute error (MAE), and largest absolute deviation (LAD) for hydrogen-bond energies (in kcal $mol^{–1}$) without and with counterpoise corrections (CPC), and hydrogen bond distances (in Å), and the Cartesian root-mean-square deviation (RMSD) analysis of the complete hydrogen-bonded complex for selected DFT functionals. Computed at ZORA-DFT/TZ2P.

Table S1. Harmonic vibrational frequencies (in cm^{-1}) of the fragments in of the neutral $H_mX{\bullet}{\bullet}{\bullet}H_mY$, cationic $H_{m+1}X^+{\bullet}{\bullet}{\bullet}H_mY$, and anionic $H_mX{\bullet}{\bullet}{\bullet}H_{m-1}Y^-$ complexes, computed at CCSD(T)/aVTZ.

NH_3

Mode	Frequency	Mode	Frequency
ω_1	1063.01	ω_4	3463.82
ω_2	1672.45	ω_5	3592.46
ω_3	1672.54	ω_6	3592.58

PH_3

Mode	Frequency	Mode	Frequency
ω_1	1012.71	ω_4	2409.08
ω_2	1142.85	ω_5	2416.25
ω_3	1142.94	ω_6	2416.33

AsH_3

Mode	Frequency	Mode	Frequency
ω_1	928.6	ω_4	2189.93
ω_2	1021.39	ω_5	2200.68
ω_3	1021.46	ω_6	2200.78

H_2O

Mode	Frequency	Mode	Frequency
ω_1	1645.59	ω_3	3919.73
ω_2	3810.66		

H_2S

Mode	Frequency	Mode	Frequency
ω_1	1211.16	ω_3	2731.17
ω_2	2715.76		

H_2Se

Mode	Frequency	Mode	Frequency
ω_1	1062.19	ω_3	2438.50
ω_2	2424.13		

HF

Mode	Frequency
ω_1	4124.97

HCl

Mode	Frequency
ω_1	2995.59

HBr

Mode	Frequency
ω_1	2644.22

NH_4^+

Mode	Frequency	Mode	Frequency
ω_1	1492.12	ω_6	3378.07
ω_2	1492.24	ω_7	3498.50
ω_3	1492.25	ω_8	3498.87
ω_4	1742.27	ω_9	3498.95
ω_5	1742.34		

H_3O^+

Mode	Frequency	Mode	Frequency
ω_1	919.41	ω_4	3585.48
ω_2	1700.07	ω_5	3682.52
ω_3	1700.16	ω_6	3682.85

H_2F^+

Mode	Frequency	Mode	Frequency
ω_1	1454.48	ω_3	3537.44
ω_2	3534.16		

NH_2^-

Mode	Frequency	Mode	Frequency
ω_1	1486.05	ω_3	3358.38
ω_2	3275.47		

PH_2^-

Mode	Frequency	Mode	Frequency
ω_1	1082.08	ω_3	2284.47
ω_2	2283.26		

AsH_2^-

Mode	Frequency	Mode	Frequency
ω_1	966.86	ω_3	2062.25
ω_2	2057.50		

OH^-

Mode	Frequency
ω_1	3722.68

SH^-

Mode	Frequency
ω_1	2641.56

SeH^-

Mode	Frequency
ω_1	2339.98

Table S2. Harmonic vibrational frequencies (in cm^{-1}) of the neutral $H_mX\cdots H_mY$ complexes, computed at CCSD(T)/aVTZ.

$NH_3\cdots NH_3$

Mode	Frequency	Mode	Frequency
ω_1	44.32i	ω_{10}	1671.75
ω_2	71.58	ω_{11}	1672.64
ω_3	114.39	ω_{12}	1700.38
ω_4	141.19	ω_{13}	3435.36
ω_5	254.93	ω_{14}	3461.82
ω_6	364.71	ω_{15}	3559.77
ω_7	1086.84	ω_{16}	3586.73
ω_8	1093.05	ω_{17}	3587.69
ω_9	1668.09	ω_{18}	3591.57

NH_3•••PH_3

Mode	Frequency	Mode	Frequency
ω_1	6.48	ω_{10}	1144.36
ω_2	82.33	ω_{11}	1670.13
ω_3	83.93	ω_{12}	1673.42
ω_4	102.92	ω_{13}	2400.63
ω_5	163.08	ω_{14}	2428.28
ω_6	205.43	ω_{15}	2434.23
ω_7	1014.22	ω_{16}	3458.99
ω_8	1074.92	ω_{17}	3587.44
ω_9	1133.79	ω_{18}	3588.58

H_2O•••H_2O

Mode	Frequency	Mode	Frequency
ω_1	131.48	ω_7	1646.30
ω_2	147.02	ω_8	1667.45
ω_3	153.83	ω_9	3731.09
ω_4	184.68	ω_{10}	3805.22
ω_5	359.54	ω_{11}	3891.29
ω_6	623.98	ω_{12}	3910.83

H_2O•••H_2S

Mode	Frequency	Mode	Frequency
ω_1	68.76	ω_7	1209.46
ω_2	89.99	ω_8	1652.88
ω_3	110.31	ω_9	2712.29
ω_4	127.46	ω_{10}	2727.40
ω_5	275.41	ω_{11}	3759.86
ω_6	430.99	ω_{12}	3891.87

H_2O•••H_2Se

Mode	Frequency	Mode	Frequency
ω_1	39.66	ω_7	1061.37
ω_2	80.52	ω_8	1651.10
ω_3	105.13	ω_9	2423.15
ω_4	180.61	ω_{10}	2437.17
ω_5	253.14	ω_{11}	3772.64
ω_6	358.88	ω_{12}	3880.18

HF•••HF

Mode	Frequency	Mode	Frequency
ω_1	161.56	ω_4	579.15
ω_2	220.57	ω_5	4009.17
ω_3	473.01	ω_6	4088.22

HF•••HCl

Mode	Frequency	Mode	Frequency
ω_1	118.04	ω_4	483.62
ω_2	161.38	ω_5	2978.00
ω_3	373.96	ω_6	4011.02

HF•••HBr

Mode	Frequency	Mode	Frequency
ω_1	103.47	ω_4	448.97
ω_2	141.76	ω_5	2633.35
ω_3	353.19	ω_6	4004.93

Table S3. Harmonic vibrational frequencies (in cm^{-1}) of the cationic $H_{m+1}X^{+}$•••H_mY complexes, computed at CCSD(T)/aVTZ.

NH_4^+•••NH_3

Mode	Frequency	Mode	Frequency
ω_1	28.88	ω_{12}	1671.53
ω_2	312.65	ω_{13}	1778.19
ω_3	373.28	ω_{14}	1778.25
ω_4	373.59	ω_{15}	2051.50
ω_5	576.12	ω_{16}	3433.83
ω_6	576.29	ω_{17}	3446.57
ω_7	1273.17	ω_{18}	3537.09
ω_8	1395.93	ω_{19}	3537.34
ω_9	1574.86	ω_{20}	3553.96
ω_{10}	1574.94	ω_{21}	3554.23
ω_{11}	1671.25		

NH_4^+•••PH_3

Mode	Frequency	Mode	Frequency
ω_1	13.44i	ω_{12}	1519.08
ω_2	196.95	ω_{13}	1744.37
ω_3	215.91	ω_{14}	1744.46
ω_4	216.26	ω_{15}	2464.01
ω_5	341.17	ω_{16}	2479.33
ω_6	341.30	ω_{17}	2479.45
ω_7	992.79	ω_{18}	2893.66
ω_8	1128.98	ω_{19}	3423.43
ω_9	1129.08	ω_{20}	3521.53
ω_{10}	1449.17	ω_{21}	3521.67
ω_{11}	1519.02		

NH_4^+•••AsH_3

Mode	Frequency	Mode	Frequency
ω_1	15.30i	ω_{12}	1513.92
ω_2	167.52	ω_{13}	1742.63
ω_3	189.38	ω_{14}	1742.70
ω_4	189.73	ω_{15}	2261.54
ω_5	310.28	ω_{16}	2277.07
ω_6	310.47	ω_{17}	2277.18
ω_7	906.15	ω_{18}	2965.62
ω_8	1014.96	ω_{19}	3422.23
ω_9	1015.05	ω_{20}	3519.06
ω_{10}	1453.24	ω_{21}	3519.30
ω_{11}	1513.87		

H_3O^+•••H_2O

Mode	Frequency	Mode	Frequency
ω_1	169.26	ω_9	1563.56
ω_2	339.18	ω_{10}	1719.17
ω_3	464.57	ω_{11}	1768.40
ω_4	535.78	ω_{12}	3739.10
ω_5	543.37	ω_{13}	3746.54
ω_6	625.58	ω_{14}	3831.44
ω_7	850.01	ω_{15}	3831.86
ω_8	1481.11		

H_3O^+•••H_2S

Mode	Frequency	Mode	Frequency
ω_1	75.62	ω_9	1452.80
ω_2	282.11	ω_{10}	1625.67
ω_3	372.26	ω_{11}	1771.35
ω_4	405.76	ω_{12}	2684.61
ω_5	565.60	ω_{13}	2697.74
ω_6	597.72	ω_{14}	3684.16
ω_7	1155.67	ω_{15}	3772.59
ω_8	1197.43		

H_3O^+•••H_2Se

Mode	Frequency	Mode	Frequency
ω_1	62.47	ω_9	1536.64
ω_2	248.39	ω_{10}	1608.37
ω_3	341.85	ω_{11}	1807.86
ω_4	376.22	ω_{12}	2407.85
ω_5	524.13	ω_{13}	2420.26
ω_6	582.14	ω_{14}	3675.68
ω_7	1054.52	ω_{15}	3764.68
ω_8	1107.56		

H_2F^+•••HF

Mode	Frequency	Mode	Frequency
ω_1	79.65	ω_6	1380.44
ω_2	484.51	ω_7	1708.52
ω_3	595.33	ω_8	3787.65
ω_4	802.23	ω_9	3798.08
ω_5	1034.24		

H_2F^+•••HCl

Mode	Frequency	Mode	Frequency
ω_1	93.22	ω_6	1257.84
ω_2	259.39	ω_7	1854.98
ω_3	365.01	ω_8	2817.65
ω_4	472.14	ω_9	3924.10
ω_5	771.00		

H_2F^+•••HBr

Mode	Frequency	Mode	Frequency
ω_1	83.06	ω_6	1111.95
ω_2	193.07	ω_7	1891.32
ω_3	307.71	ω_8	2523.30
ω_4	388.03	ω_9	3952.05
ω_5	644.18		

Table S4. Harmonic vibrational frequencies (in cm^{-1}) of the anionic $H_mX\bullet\bullet\bullet H_{m-1}Y^-$complexes, computed at CCSD(T)/aVTZ.

$NH_3\bullet\bullet\bullet NH_2^-$

Mode	Frequency	Mode	Frequency
ω_1	124.91	ω_9	1652.94
ω_2	153.41	ω_{10}	1708.64
ω_3	173.47	ω_{11}	2836.78
ω_4	238.32	ω_{12}	3319.78
ω_5	367.19	ω_{13}	3403.90
ω_6	514.25	ω_{14}	3455.80
ω_7	1268.26	ω_{15}	3532.53
ω_8	1498.59		

$NH_3\bullet\bullet\bullet PH_2^-$

Mode	Frequency	Mode	Frequency
ω_1	39.44	ω_9	1658.75
ω_2	97.17	ω_{10}	1693.67
ω_3	107.94	ω_{11}	2287.34
ω_4	126.23	ω_{12}	2294.05
ω_5	256.71	ω_{13}	3272.02
ω_6	348.58	ω_{14}	3480.20
ω_7	1081.27	ω_{15}	3547.40
ω_8	1188.56		

NH_3•••AsH_2^-

Mode	Frequency	Mode	Frequency
ω_1	21.66	ω_9	1660.77
ω_2	79.65	ω_{10}	1692.06
ω_3	99.16	ω_{11}	2058.59
ω_4	103.95	ω_{12}	2063.34
ω_5	242.38	ω_{13}	3305.98
ω_6	309.31	ω_{14}	3483.34
ω_7	966.29	ω_{15}	3547.23
ω_8	1187.08		

H_2O•••OH^-

Mode	Frequency	Mode	Frequency
ω_1	188.92	ω_6	1672.00
ω_2	390.42	ω_7	2153.74
ω_3	451.80	ω_8	3809.57
ω_4	542.62	ω_9	3835.67
ω_5	1276.50		

H_2O•••SH^-

Mode	Frequency	Mode	Frequency
ω_1	153.40	ω_6	1676.06
ω_2	188.95	ω_7	2654.72
ω_3	217.35	ω_8	3318.18
ω_4	351.41	ω_9	3859.03
ω_5	795.38		

H_2O•••SeH^-

Mode	Frequency	Mode	Frequency
ω_1	141.47	ω_6	1672.17
ω_2	153.01	ω_7	2352.56
ω_3	186.14	ω_8	3420.45
ω_4	310.23	ω_9	3858.33
ω_5	722.57		

HF•••F^-

Mode	Frequency	Mode	Frequency
ω_1	641.03	ω_3	1366.10
ω_2	1270.59	ω_4	1366.10

$HF\cdots Cl^-$

Mode	Frequency	Mode	Frequency
ω_1	254.24	ω_3	880.34
ω_2	880.34	ω_4	3119.41

$HF\cdots Br^-$

Mode	Frequency	Mode	Frequency
ω_1	200.85	ω_3	796.13
ω_2	796.13	ω_4	3308.63

Table S5. Harmonic vibrational frequencies (in cm^{-1}) of the H_2CO•••**Am–X** complexes, computed at CCSD(T)/jul-VTZ.

Formaldehyde (H_2CO)

Mode	Frequency	Mode	Frequency
ω_1	0.00i	ω_7	1187.12
ω_2	0.00i	ω_8	1265.82
ω_3	0.00i	ω_9	1534.39
ω_4	0.00	ω_{10}	1766.65
ω_5	0.00	ω_{11}	2932.47
ω_6	0.00	ω_{12}	3002.06

Carboxamide (Am–O)

Mode	Frequency	Mode	Frequency
ω_1	0.00i	ω_{10}	1042.08
ω_2	0.00i	ω_{11}	1051.09
ω_3	0.00i	ω_{12}	1271.37
ω_4	0.00i	ω_{13}	1417.34
ω_5	0.00	ω_{14}	1619.74
ω_6	0.00	ω_{16}	1782.18
ω_7	34.35	ω_{16}	2989.32
ω_8	562.45	ω_{17}	3597.32
ω_9	629.67	ω_{18}	3742.14

Thioamide (Am–S)

Mode	Frequency	Mode	Frequency
ω_1	0.00i	ω_{10}	885.77
ω_2	0.00i	ω_{11}	959.38
ω_3	0.00	ω_{12}	1136.77
ω_4	0.00	ω_{13}	1306.21
ω_5	0.00	ω_{14}	1461.31
ω_6	0.00	ω_{16}	1639.20
ω_7	288.00	ω_{16}	3109.87
ω_8	429.67	ω_{17}	3572.91
ω_9	615.68	ω_{18}	3715.99

H_2CO•••**Am–O**

Mode	Frequency	Mode	Frequency
ω_1	0.00i	ω_{16}	1045.00
ω_2	0.00i	ω_{17}	1084.01
ω_3	0.00i	ω_{18}	1196.76
ω_4	0.00	ω_{19}	1269.38
ω_5	0.00	ω_{20}	1280.85
ω_6	0.00	ω_{21}	1415.95
ω_7	26.14	ω_{22}	1530.90
ω_8	55.08	ω_{23}	1639.16
ω_9	60.29	ω_{24}	1755.52
ω_{10}	96.84	ω_{25}	1776.39
ω_{11}	162.79	ω_{26}	2952.53
ω_{12}	173.20	ω_{27}	2977.59
ω_{13}	431.28	ω_{28}	3035.51
ω_{14}	577.22	ω_{29}	3534.71
ω_{15}	713.37	ω_{30}	3690.19

H_2CO•••**Am–S**

Mode	Frequency	Mode	Frequency
ω_1	0.00i	ω_{16}	893.75
ω_2	0.00i	ω_{17}	972.59
ω_3	0.00i	ω_{18}	1153.71
ω_4	0.00i	ω_{19}	1196.95
ω_5	0.00	ω_{20}	1269.21
ω_6	0.00	ω_{21}	1316.63
ω_7	22.12	ω_{22}	1472.77
ω_8	51.04	ω_{23}	1531.62
ω_9	58.13	ω_{24}	1650.91
ω_{10}	92.91	ω_{25}	1755.07
ω_{11}	166.07	ω_{26}	2953.47
ω_{12}	170.81	ω_{27}	3038.21
ω_{13}	440.97	ω_{28}	3100.29
ω_{14}	500.07	ω_{29}	3507.63
ω_{15}	699.74	ω_{30}	3668.78

Table S6. Harmonic vibrational frequencies (in cm^{-1}) of the H_2CO•••**Ur–X** complexes, computed at CCSD(T)/jul-VTZ.

Urea (Ur–O)

Mode	Frequency	Mode	Frequency
ω_1	0.00i	ω_{13}	783.76
ω_2	0.00i	ω_{14}	949.38
ω_3	0.00i	ω_{15}	1064.70
ω_4	0.00	ω_{16}	1190.70
ω_5	0.00	ω_{17}	1417.83
ω_6	0.00	ω_{18}	1637.47
ω_7	375.52	ω_{19}	1643.15
ω_8	448.20	ω_{20}	1799.33
ω_9	471.53	ω_{21}	3569.22
ω_{10}	548.16	ω_{22}	3572.15
ω_{11}	582.64	ω_{23}	3685.24
ω_{12}	601.56	ω_{24}	3685.83

Urea (Ur–O) – C_{2v}

Mode	Frequency	Mode	Frequency
ω_1	423.64i	ω_{13}	772.20
ω_2	340.57i	ω_{14}	970.52
ω_3	0.00i	ω_{15}	990.00
ω_4	0.00i	ω_{16}	1143.40
ω_5	0.00i	ω_{17}	1428.10
ω_6	0.00i	ω_{18}	1626.61
ω_7	0.00i	ω_{19}	1627.74
ω_8	0.00	ω_{20}	1777.61
ω_9	363.56	ω_{21}	3627.88
ω_{10}	475.43	ω_{22}	3635.23
ω_{11}	562.26	ω_{23}	3768.81
ω_{12}	567.62	ω_{24}	3771.25

Thiourea (Ur–S)

Mode	Frequency	Mode	Frequency
ω_1	0.00i	ω_{13}	639.13
ω_2	0.00i	ω_{14}	771.99
ω_3	0.00i	ω_{15}	1073.21
ω_4	0.00i	ω_{16}	1084.37
ω_5	0.00	ω_{17}	1421.59
ω_6	0.00	ω_{18}	1423.07
ω_7	358.06	ω_{19}	1637.30
ω_8	395.39	ω_{20}	1659.16
ω_9	398.95	ω_{21}	3562.33
ω_{10}	455.29	ω_{22}	3567.17
ω_{11}	521.95	ω_{23}	3692.71
ω_{12}	581.91	ω_{24}	3693.38

Thiourea (Ur–S) – C_{2v}

Mode	Frequency	Mode	Frequency
ω_1	314.84i	ω_{13}	644.63
ω_2	181.96i	ω_{14}	766.61
ω_3	0.00i	ω_{15}	1029.27
ω_4	0.00i	ω_{16}	1059.48
ω_5	0.00	ω_{17}	1405.55
ω_6	0.00	ω_{18}	1444.37
ω_7	0.00	ω_{19}	1625.58
ω_8	0.00	ω_{20}	1651.46
ω_9	394.46	ω_{21}	3597.58
ω_{10}	411.24	ω_{22}	3605.13
ω_{11}	454.69	ω_{23}	3743.68
ω_{12}	594.07	ω_{24}	3745.08

H_2CO•••**Ur–O**

Mode	Frequency	Mode	Frequency
ω_1	0.00i	ω_{19}	777.57
ω_2	0.00i	ω_{20}	959.47
ω_3	0.00i	ω_{21}	1065.39
ω_4	0.00i	ω_{22}	1194.85
ω_5	0.00	ω_{23}	1199.52
ω_6	0.00	ω_{24}	1267.68
ω_7	18.74	ω_{25}	1423.66
ω_8	46.22	ω_{26}	1534.35
ω_9	93.28	ω_{27}	1645.86
ω_{10}	105.44	ω_{28}	1645.91
ω_{11}	110.22	ω_{29}	1762.37
ω_{12}	113.14	ω_{30}	1783.25
ω_{13}	443.99	ω_{31}	2957.11
ω_{14}	469.98	ω_{32}	3038.28
ω_{15}	491.93	ω_{33}	3572.74
ω_{16}	504.81	ω_{34}	3578.49
ω_{17}	539.94	ω_{35}	3701.87
ω_{18}	589.99	ω_{36}	3704.26

H_2CO•••**Ur–O** – C_{2v}

Mode	Frequency	Mode	Frequency
ω_1	343.93i	ω_{19}	780.10
ω_2	263.29i	ω_{20}	975.05
ω_3	0.00i	ω_{21}	1017.51
ω_4	0.00i	ω_{22}	1162.77
ω_5	0.00	ω_{23}	1195.77
ω_6	0.00	ω_{24}	1267.76
ω_7	0.00	ω_{25}	1428.19
ω_8	0.00	ω_{26}	1534.22
ω_9	26.85	ω_{27}	1632.30
ω_{10}	45.79	ω_{28}	1634.12
ω_{11}	110.30	ω_{29}	1761.43
ω_{12}	111.78	ω_{30}	1768.77
ω_{13}	119.22	ω_{31}	2959.40
ω_{14}	151.15	ω_{32}	3041.81
ω_{15}	457.60	ω_{33}	3611.46
ω_{16}	493.12	ω_{34}	3621.89
ω_{17}	563.36	ω_{35}	3761.11
ω_{18}	625.84	ω_{36}	3764.28

$H_2CO\cdots$**Ur–S**

Mode	Frequency	Mode	Frequency
ω_1	0.00i	ω_{19}	656.15
ω_2	0.00i	ω_{20}	769.69
ω_3	0.00i	ω_{21}	1074.96
ω_4	0.00	ω_{22}	1075.70
ω_5	0.00	ω_{23}	1197.30
ω_6	0.00	ω_{24}	1267.97
ω_7	22.70	ω_{25}	1430.25
ω_8	46.36	ω_{26}	1442.03
ω_9	70.42	ω_{27}	1534.38
ω_{10}	103.98	ω_{28}	1636.43
ω_{11}	108.87	ω_{29}	1658.00
ω_{12}	109.61	ω_{30}	1760.66
ω_{13}	284.14	ω_{31}	2962.98
ω_{14}	332.90	ω_{32}	3046.96
ω_{15}	396.71	ω_{33}	3569.29
ω_{16}	469.44	ω_{34}	3579.99
ω_{17}	492.00	ω_{35}	3723.24
ω_{18}	628.00	ω_{36}	3726.03

H_2CO•••**Ur–S** – C_{2v}

Mode	Frequency	Mode	Frequency
ω_1	146.68i	ω_{19}	672.2903
ω_2	59.91i	ω_{20}	767.6791
ω_3	0.00i	ω_{21}	1058.53
ω_4	0.00i	ω_{22}	1072.188
ω_5	0.00	ω_{23}	1197.599
ω_6	0.00	ω_{24}	1268.005
ω_7	0.00	ω_{25}	1424.387
ω_8	0.00	ω_{26}	1447.352
ω_9	26.8775	ω_{27}	1534.336
ω_{10}	46.8151	ω_{28}	1631.829
ω_{11}	110.8046	ω_{29}	1654.346
ω_{12}	111.7088	ω_{30}	1760.42
ω_{13}	145.1642	ω_{31}	2963.792
ω_{14}	237.0507	ω_{32}	3048.199
ω_{15}	396.0063	ω_{33}	3578.236
ω_{16}	469.9647	ω_{34}	3590.533
ω_{17}	496.768	ω_{35}	3737.908
ω_{18}	633.2033	ω_{36}	3741.071

Table S7. Harmonic vibrational frequencies (in cm^{-1}) of the H_2CO•••**Delt–X** complexes, computed at CCSD(T)/jul-VDZ.

2,3-diaminocycloprop-2-en-1-one (Delt–O)

Mode	Frequency	Mode	Frequency
ω_1	477.34i	ω_{16}	646.81
ω_2	459.45i	ω_{17}	756.38
ω_3	0.00i	ω_{18}	829.05
ω_4	0.00i	ω_{19}	911.61
ω_5	0.00i	ω_{20}	1178.89
ω_6	0.00	ω_{21}	1224.25
ω_7	0.00	ω_{22}	1376.42
ω_8	0.00	ω_{23}	1627.26
ω_9	179.29	ω_{24}	1632.85
ω_{10}	217.41	ω_{25}	1702.70
ω_{11}	237.94	ω_{26}	1966.49
ω_{12}	345.63	ω_{27}	3609.42
ω_{13}	400.53	ω_{28}	3612.73
ω_{14}	565.60	ω_{29}	3744.25
ω_{15}	613.88	ω_{30}	3744.71

2,3-diaminocycloprop-2-en-1-thione (Delt–S)

Mode	Frequency	Mode	Frequency
ω_1	406.77i	ω_{16}	584.14
ω_2	387.87i	ω_{17}	602.37
ω_3	0.00i	ω_{18}	845.54
ω_4	0.00i	ω_{19}	853.78
ω_5	0.00i	ω_{20}	1123.43
ω_6	0.00i	ω_{21}	1189.40
ω_7	0.00	ω_{22}	1428.94
ω_8	0.00	ω_{23}	1438.90
ω_9	162.00	ω_{24}	1632.09
ω_{10}	172.89	ω_{25}	1634.60
ω_{11}	212.38	ω_{26}	1952.86
ω_{12}	386.68	ω_{27}	3602.37
ω_{13}	454.12	ω_{28}	3605.05
ω_{14}	516.19	ω_{29}	3736.56
ω_{15}	558.15	ω_{30}	3737.07

H_2CO•••**Delt–O**

Mode	Frequency	Mode	Frequency	Mode	Frequency
ω_1	447.46i	ω_{15}	191.12	ω_{29}	1247.47
ω_2	436.71i	ω_{16}	228.96	ω_{30}	1387.39
ω_3	15.45i	ω_{17}	242.48	ω_{31}	1515.20
ω_4	0.00i	ω_{18}	453.94	ω_{32}	1610.78
ω_5	0.00i	ω_{19}	492.71	ω_{33}	1616.96
ω_6	0.00i	ω_{20}	576.16	ω_{34}	1693.63
ω_7	0.00i	ω_{21}	617.13	ω_{35}	1731.70
ω_8	0.00	ω_{22}	646.98	ω_{36}	1964.17
ω_9	0.00	ω_{23}	758.54	ω_{37}	2954.37
ω_{10}	59.37	ω_{24}	832.29	ω_{38}	3044.24
ω_{11}	84.57	ω_{25}	921.54	ω_{39}	3596.70
ω_{12}	90.11	ω_{26}	1170.25	ω_{40}	3601.99
ω_{13}	99.17	ω_{27}	1188.53	ω_{41}	3743.60
ω_{14}	136.35	ω_{28}	1235.72	ω_{42}	3744.53

H_2CO•••**Delt–S**

Mode	Frequency	Mode	Frequency	Mode	Frequency
ω_1	370.74i	ω_{15}	170.20	ω_{29}	1247.80
ω_2	358.73i	ω_{16}	183.31	ω_{30}	1435.79
ω_3	10.55i	ω_{17}	221.23	ω_{31}	1439.67
ω_4	0.00i	ω_{18}	482.87	ω_{32}	1515.49
ω_5	0.00i	ω_{19}	517.44	ω_{33}	1612.47
ω_6	0.00	ω_{20}	539.26	ω_{34}	1616.38
ω_7	0.00	ω_{21}	570.55	ω_{35}	1730.03
ω_8	0.00	ω_{22}	581.46	ω_{36}	1956.77
ω_9	0.00	ω_{23}	595.81	ω_{37}	2956.36
ω_{10}	59.13	ω_{24}	855.02	ω_{38}	3047.17
ω_{11}	86.37	ω_{25}	859.36	ω_{39}	3593.09
ω_{12}	95.02	ω_{26}	1133.76	ω_{40}	3597.39
ω_{13}	95.20	ω_{27}	1171.18	ω_{41}	3735.70
ω_{14}	140.14	ω_{28}	1200.40	ω_{42}	3736.75

Table S8. Harmonic vibrational frequencies (in cm^{-1}) of the H_2CO•••**Squar–X** complexes, computed at CCSD(T)/jul-VDZ.

3,4-diamonocyclobut-3-ene-1,2-dione (Squar–O)

Mode	Frequency	Mode	Frequency
ω_1	384.62i	ω_{19}	675.07
ω_2	342.57i	ω_{20}	704.82
ω_3	0.00i	ω_{21}	728.63
ω_4	0.00i	ω_{22}	937.63
ω_5	0.00	ω_{23}	944.87
ω_6	0.00	ω_{24}	1048.37
ω_7	0.00	ω_{25}	1243.69
ω_8	0.00	ω_{26}	1283.09
ω_9	107.20	ω_{27}	1425.14
ω_{10}	195.75	ω_{28}	1617.57
ω_{11}	206.47	ω_{29}	1637.37
ω_{12}	237.90	ω_{30}	1687.35
ω_{13}	290.37	ω_{31}	1762.98
ω_{14}	394.21	ω_{32}	1852.22
ω_{15}	453.40	ω_{33}	3591.91
ω_{16}	551.33	ω_{34}	3593.50
ω_{17}	584.89	ω_{35}	3724.23
ω_{18}	601.97	ω_{36}	3724.38

3,4-diamonocyclobut-3-ene-1,2-dithione (Squar–S)

Mode	Frequency	Mode	Frequency
ω_1	305.81i	ω_{19}	546.50
ω_2	251.63i	ω_{20}	615.29
ω_3	0.00i	ω_{21}	706.08
ω_4	0.00i	ω_{22}	872.87
ω_5	0.00i	ω_{23}	875.17
ω_6	0.00i	ω_{24}	1015.12
ω_7	0.00	ω_{25}	1165.02
ω_8	0.00	ω_{26}	1217.04
ω_9	84.30	ω_{27}	1343.76
ω_{10}	144.26	ω_{28}	1408.82
ω_{11}	163.02	ω_{29}	1456.45
ω_{12}	191.62	ω_{30}	1613.59
ω_{13}	233.26	ω_{31}	1636.22
ω_{14}	448.20	ω_{32}	1758.30
ω_{15}	471.80	ω_{33}	3568.06
ω_{16}	495.57	ω_{34}	3568.19
ω_{17}	512.45	ω_{35}	3714.73
ω_{18}	543.69	ω_{36}	3715.92

H_2CO••• **Squar–O**

Mode	Frequency	Mode	Frequency	Mode	Frequency
ω_1	271.90i	ω_{17}	226.17	ω_{33}	1255.87
ω_2	242.03i	ω_{18}	252.83	ω_{34}	1305.35
ω_3	0.00i	ω_{19}	294.29	ω_{35}	1438.26
ω_4	0.00i	ω_{20}	510.55	ω_{36}	1515.44
ω_5	0.00i	ω_{21}	547.70	ω_{37}	1610.61
ω_6	0.00	ω_{22}	562.99	ω_{38}	1634.79
ω_7	0.00	ω_{23}	603.88	ω_{39}	1681.69
ω_8	0.00	ω_{24}	607.68	ω_{40}	1729.73
ω_9	20.95	ω_{25}	677.90	ω_{41}	1752.70
ω_{10}	57.38	ω_{26}	713.66	ω_{42}	1848.63
ω_{11}	92.51	ω_{27}	731.01	ω_{43}	2965.73
ω_{12}	107.77	ω_{28}	951.96	ω_{44}	3060.46
ω_{13}	122.24	ω_{29}	956.96	ω_{45}	3567.46
ω_{14}	130.16	ω_{30}	1056.58	ω_{46}	3574.52
ω_{15}	162.74	ω_{31}	1174.71	ω_{47}	3706.79
ω_{16}	212.85	ω_{32}	1248.44	ω_{48}	3709.50

H_2CO••• **Squar–S**

Mode	Frequency	Mode	Frequency	Mode	Frequency
ω_1	95.17i	ω_{17}	205.73	ω_{33}	1228.71
ω_2	90.66i	ω_{18}	209.09	ω_{34}	1248.98
ω_3	0.00i	ω_{19}	257.12	ω_{35}	1343.62
ω_4	0.00i	ω_{20}	496.38	ω_{36}	1415.62
ω_5	0.00i	ω_{21}	513.90	ω_{37}	1471.19
ω_6	0.00i	ω_{22}	514.60	ω_{38}	1515.56
ω_7	0.00	ω_{23}	543.50	ω_{39}	1609.36
ω_8	0.00	ω_{24}	559.26	ω_{40}	1633.79
ω_9	20.26	ω_{25}	613.74	ω_{41}	1728.20
ω_{10}	55.43	ω_{26}	619.87	ω_{42}	1758.80
ω_{11}	86.84	ω_{27}	713.18	ω_{43}	2968.39
ω_{12}	90.93	ω_{28}	881.81	ω_{44}	3064.35
ω_{13}	118.13	ω_{29}	881.87	ω_{45}	3548.56
ω_{14}	152.59	ω_{30}	1030.62	ω_{46}	3553.27
ω_{15}	160.17	ω_{31}	1175.96	ω_{47}	3688.42
ω_{16}	188.38	ω_{32}	1179.44	ω_{48}	3692.74

Table S9. Focal point analysis for the NH_3•••NH_3 complex with further additive corrections at the CCSD(T) level of theory with units in kcal mol^{-1}. Bracketed values indicate the extrapolated energies or additive corrections. Additive corrections are indicated by the δ which represents the incremental change from the previous energy.

	RHF	+δMP2	+δCCSD	+δ(T)	+δT	+δ(Q)	NET
aVDZ	–1.546	–1.138	+0.249	–0.195	–0.001	–0.012	[–2.643]
aVTZ	–1.561	–1.408	+0.248	–0.243	+0.004	–0.017	[–2.977]
aVQZ	–1.573	–1.492	+0.260	–0.254	[+0.004]	[–0.017]	[–3.073]
aV5Z	–1.573	–1.521	+0.269	–0.257	[+0.004]	[–0.017]	[–3.096]
CBS	[–1.572]	[–1.552]	[+0.279]	[–0.260]	[+0.004]	[–0.017]	[–3.118]

$$\Delta E_{int}^{CP} = E_{CCSDT(Q)/CBS} + \Delta E_{core} + \Delta E_{rel} + \Delta E_{DBOC}$$

$$\Delta E_{int}^{CP} = -3.118 - 0.016 + 0.002 - 0.009 = \mathbf{-3.14}$$

	RHF	+δMP2	+δCCSD	+δ(T)	+δT	+δ(Q)	NET
aVDZ	–0.283	–0.609	+0.051	–0.079	+0.003	+0.003	[–0.914]
aVTZ	+0.046	–0.314	+0.037	–0.026	+0.002	–0.002	[–0.258]
aVQZ	+0.069	–0.169	+0.041	–0.017	[+0.002]	[–0.002]	[–0.076]
aV5Z	+0.081	–0.109	+0.028	–0.012	[+0.002]	[–0.002]	[–0.012]
CBS	[+0.087]	[–0.046]	[+0.016]	[–0.008]	[+0.002]	[–0.002]	[+0.049]

$$\Delta E_{strain} = E_{CCSDT(Q)/CBS} + \Delta E_{core} + \Delta E_{rel} + \Delta E_{DBOC}$$

$$\Delta E_{strain} = 0.049 - 0.011 + 0.003 - 0.001 = +\mathbf{0.04}$$

Table S10. Focal point analysis for the NH_3•••PH_3 complex with further additive corrections at the CCSD(T) level of theory with units in kcal mol^{-1}. Bracketed values indicate the extrapolated energies or additive corrections. Additive corrections are indicated by the δ which represents the incremental change from the previous energy.

	RHF	+δMP2	+δCCSD	+δ(T)	+δT	+δ(Q)	NET
aVDZ	–0.255	–1.208	+0.278	–0.192	+0.002	–0.011	[–1.386]
aVTZ	–0.250	–1.442	+0.296	–0.241	+0.009	–0.017	[–1.646]
aVQZ	–0.256	–1.521	+0.306	–0.257	[+0.009]	[–0.017]	[–1.736]
aV5Z	–0.259	–1.545	+0.313	–0.261	[+0.009]	[–0.017]	[–1.760]
CBS	[–0.260]	[–1.570]	[+0.321]	[–0.265]	[+0.009]	[–0.017]	[–1.782]

$$\Delta E_{int}^{CP} = E_{CCSDT(Q)/CBS} + \Delta E_{core} + \Delta E_{rel} + \Delta E_{DBOC}$$

$$\Delta E_{int}^{CP} = -1.782 - 0.008 + 0.001 - 0.001 = \mathbf{-1.78}$$

	RHF	+δMP2	+δCCSD	+δ(T)	+δT	+δ(Q)	NET
aVDZ	–0.262	–0.260	+0.027	–0.028	+0.001	+0.001	[–0.521]
aVTZ	–0.129	–0.147	+0.024	–0.004	+0.002	+0.000	[–0.254]
aVQZ	–0.095	–0.001	+0.031	+0.007	[+0.002]	[+0.000]	[–0.056]
aV5Z	–0.089	+0.045	+0.019	+0.010	[+0.002]	[+0.000]	[–0.012]
CBS	[–0.088]	[+0.094]	[+0.007]	[+0.014]	[+0.002]	[+0.000]	[+0.029]

$$\Delta E_{strain} = E_{CCSDT(Q)/CBS} + \Delta E_{core} + \Delta E_{rel} + \Delta E_{DBOC}$$

$$\Delta E_{strain} = 0.029 - 0.037 + 0.003 - 0.000 = +\mathbf{0.00}$$

Table S11. Focal point analysis for the $H_2O{\bullet}{\bullet}{\bullet}H_2O$ complex with further additive corrections at the CCSD(T) level of theory with units in kcal mol^{-1}. Bracketed values indicate the extrapolated energies or additive corrections. Additive corrections are indicated by the δ which represents the incremental change from the previous energy.

	RHF	+δMP2	+δCCSD	+δ(T)	+δT	+δ(Q)	NET
aVDZ	–3.631	–0.791	+0.219	–0.177	+0.007	–0.010	[–4.383]
aVTZ	–3.615	–1.130	+0.197	–0.230	+0.005	–0.015	[–4.787]
aVQZ	–3.651	–1.261	+0.201	–0.244	[+0.005]	[–0.015]	[–4.966]
aV5Z	–3.651	–1.309	+0.212	–0.248	[+0.005]	[–0.015]	[–5.006]
CBS	[–3.647]	[–1.360]	[+0.224]	[–0.252]	[+0.005]	[–0.015]	[–5.045]

$$\Delta E_{\text{int}}^{\text{CP}} = E_{\text{CCSDT(Q)/CBS}} + \Delta E_{\text{core}} + \Delta E_{\text{rel}} + \Delta E_{\text{DBOC}}$$

$$\Delta E_{\text{int}}^{\text{CP}} = -5.045 - 0.026 + 0.011 - 0.015 = \mathbf{-5.07}$$

	RHF	+δMP2	+δCCSD	+δ(T)	+δT	+δ(Q)	NET
aVDZ	–0.082	–0.758	+0.038	–0.117	+0.004	+0.006	[–0.909]
aVTZ	+0.098	–0.533	+0.043	–0.048	+0.003	–0.004	[–0.442]
aVQZ	+0.155	–0.336	+0.068	–0.032	[+0.003]	[–0.004]	[–0.147]
aV5Z	+0.183	–0.257	+0.063	–0.026	[+0.003]	[–0.004]	[–0.040]
CBS	[+0.197]	[–0.175]	[+0.058]	[–0.020]	[+0.003]	[–0.004]	[+0.059]

$$\Delta E_{\text{strain}} = E_{\text{CCSDT(Q)/CBS}} + \Delta E_{\text{core}} + \Delta E_{\text{rel}} + \Delta E_{\text{DBOC}}$$

$$\Delta E_{\text{strain}} = 0.059 - 0.013 + 0.010 - 0.001 = +\mathbf{0.05}$$

Table S12. Focal point analysis for the $H_2O{\cdots}H_2S$ complex with further additive corrections at the CCSD(T) level of theory with units in kcal mol^{-1}. Bracketed values indicate the extrapolated energies or additive corrections. Additive corrections are indicated by the δ which represents the incremental change from the previous energy.

	RHF	+δMP2	+δCCSD	+δ(T)	+δT	+δ(Q)	NET
aVDZ	–1.234	–1.291	+0.366	–0.217	–0.000	–0.015	[–2.392]
aVTZ	–1.193	–1.664	+0.386	–0.292	+0.007	–0.022	[–2.778]
aVQZ	–1.187	–1.773	+0.397	–0.309	[+0.007]	[–0.022]	[–2.888]
aV5Z	–1.188	–1.814	+0.409	–0.314	[+0.007]	[–0.022]	[–2.922]
CBS	[–1.189]	[–1.857]	[+0.423]	[–0.320]	[+0.007]	[–0.022]	[–2.958]

$$\Delta E_{\text{int}}^{\text{CP}} = E_{\text{CCSDT(Q)/CBS}} + \Delta E_{\text{core}} + \Delta E_{\text{rel}} + \Delta E_{\text{DBOC}}$$

$$\Delta E_{\text{int}}^{\text{CP}} = -2.958 - 0.015 + 0.002 - 0.008 = \mathbf{-2.98}$$

	RHF	+δMP2	+δCCSD	+δ(T)	+δT	+δ(Q)	NET
aVDZ	–0.182	–0.696	+0.014	–0.088	–0.001	–0.001	[–0.954]
aVTZ	+0.041	–0.483	+0.062	–0.042	+0.003	–0.003	[–0.422]
aVQZ	+0.095	–0.274	+0.075	–0.025	[+0.003]	[–0.003]	[–0.130]
aV5Z	+0.110	–0.192	+0.057	–0.020	[+0.003]	[–0.003]	[–0.045]
CBS	[+0.116]	[–0.106]	[+0.040]	[–0.014]	[+0.003]	[–0.003]	[+0.035]

$$\Delta E_{\text{strain}} = E_{\text{CCSDT(Q)/CBS}} + \Delta E_{\text{core}} + \Delta E_{\text{rel}} + \Delta E_{\text{DBOC}}$$

$$\Delta E_{\text{strain}} = 0.035 - 0.080 + 0.004 - 0.001 = \mathbf{-0.04}$$

Table S13. Focal point analysis for the $H_2O{\bullet}{\bullet}{\bullet}H_2Se$ complex with further additive corrections at the CCSD(T) level of theory with units in kcal mol^{-1}. Bracketed values indicate the extrapolated energies or additive corrections. Additive corrections are indicated by the δ which represents the incremental change from the previous energy.

	RHF	+δMP2	+δCCSD	+δ(T)	+δT	+δ(Q)	NET
aVDZ	–0.954	–1.324	+0.325	–0.215	+0.000	–0.014	[–2.182]
aVTZ	–0.901	–1.673	+0.348	–0.288	+0.007	–0.020	[–2.527]
aVQZ	–0.895	–1.779	+0.360	–0.304	[+0.007]	[–0.020]	[–2.631]
aV5Z	–0.894	–1.817	+0.372	–0.309	[+0.007]	[–0.020]	[–2.661]
CBS	[–0.894]	[–1.857]	[+0.385]	[–0.315]	[+0.007]	[–0.020]	[–2.694]

$$\Delta E_{\text{int}}^{\text{CP}} = E_{\text{CCSDT(Q)/CBS}} + \Delta E_{\text{core}}$$

$$\Delta E_{\text{int}}^{\text{CP}} = -2.694 - 0.035 = \mathbf{-2.73}$$

	RHF	+δMP2	+δCCSD	+δ(T)	+δT	+δ(Q)	NET
aVDZ	–0.088	–0.756	+0.004	–0.083	–0.001	–0.001	[–0.923]
aVTZ	+0.068	–0.448	+0.065	–0.040	+0.003	–0.003	[–0.355]
aVQZ	+0.098	–0.267	+0.076	–0.024	[+0.003]	[–0.003]	[–0.118]
aV5Z	+0.106	–0.185	+0.056	–0.018	[+0.003]	[–0.003]	[–0.042]
CBS	[+0.109]	[–0.099]	[+0.034]	[–0.012]	[+0.003]	[–0.003]	[+0.032]

$$\Delta E_{\text{strain}} = E_{\text{CCSDT(Q)/CBS}} + \Delta E_{\text{core}}$$

$$\Delta E_{\text{strain}} = 0.032 - 0.248 = \mathbf{-0.22}$$

Table S14. Focal point analysis for the HF•••HF complex with further additive corrections at the CCSD(T) level of theory with units in kcal mol^{-1}. Bracketed values indicate the extrapolated energies or additive corrections. Additive corrections are indicated by the δ which represents the incremental change from the previous energy.

	RHF	+δMP2	+δCCSD	+δ(T)	+δT	+δ(Q)	NET
aVDZ	–3.782	–0.192	+0.073	–0.108	+0.013	–0.006	[–4.002]
aVTZ	–3.770	–0.468	+0.042	–0.145	+0.003	–0.010	[–4.348]
aVQZ	–3.845	–0.584	+0.031	–0.155	[+0.003]	[–0.010]	[–4.561]
aV5Z	–3.850	–0.627	+0.037	–0.158	[+0.003]	[–0.010]	[–4.605]
CBS	[–3.844]	[–0.672]	[+0.043]	[–0.161]	[+0.003]	[–0.010]	[–4.641]

$$\Delta E_{int}^{CP} = E_{CCSDT(Q)/CBS} + \Delta E_{core} + \Delta E_{rel} + \Delta E_{DBOC}$$
$$\Delta E_{int}^{CP} = -4.641 - 0.020 + 0.010 - 0.012 = \mathbf{-4.66}$$

	RHF	+δMP2	+δCCSD	+δ(T)	+δT	+δ(Q)	NET
aVDZ	+0.048	–0.754	+0.024	–0.121	–0.000	+0.004	[–0.800]
aVTZ	+0.187	–0.656	+0.037	–0.056	+0.001	–0.006	[–0.493]
aVQZ	+0.273	–0.480	+0.078	–0.043	[+0.001]	[–0.006]	[–0.177]
aV5Z	+0.323	–0.428	+0.092	–0.042	[+0.001]	[–0.006]	[–0.061]
CBS	[+0.354]	[–0.375]	[+0.106]	[–0.040]	[+0.001]	[–0.006]	[+0.040]

$$\Delta E_{strain} = E_{CCSDT(Q)/CBS} + \Delta E_{core} + \Delta E_{rel} + \Delta E_{DBOC}$$
$$\Delta E_{strain} = 0.040 - 0.010 + 0.019 - 0.001 = +\mathbf{0.05}$$

Table S15. Focal point analysis for the HF•••HCl complex with further additive corrections at the CCSD(T) level of theory with units in kcal mol^{-1}. Bracketed values indicate the extrapolated energies or additive corrections. Additive corrections are indicated by the δ which represents the incremental change from the previous energy.

	RHF	+δMP2	+δCCSD	+δ(T)	+δT	+δ(Q)	NET
aVDZ	–1.414	–0.899	+0.269	–0.169	–0.002	–0.008	[–2.222]
aVTZ	–1.512	–1.296	+0.288	–0.246	+0.000	–0.014	[–2.780]
aVQZ	–1.521	–1.411	+0.293	–0.262	[+0.000]	[–0.014]	[–2.914]
aV5Z	–1.522	–1.456	+0.304	–0.267	[+0.000]	[–0.014]	[–2.955]
CBS	[–1.522]	[–1.503]	[+0.316]	[–0.273]	[+0.000]	[–0.014]	[–2.997]

$$\Delta E_{int}^{CP} = E_{CCSDT(Q)/CBS} + \Delta E_{core} + \Delta E_{rel} + \Delta E_{DBOC}$$

$$\Delta E_{int}^{CP} = -2.997 - 0.014 + 0.003 - 0.007 = \mathbf{-3.01}$$

	RHF	+δMP2	+δCCSD	+δ(T)	+δT	+δ(Q)	NET
aVDZ	–0.035	–0.736	–0.003	–0.101	–0.003	+0.000	[–0.879]
aVTZ	+0.138	–0.640	+0.046	–0.057	+0.002	–0.005	[–0.515]
aVQZ	+0.195	–0.421	+0.093	–0.039	[+0.002]	[–0.005]	[–0.175]
aV5Z	+0.218	–0.347	+0.090	–0.034	[+0.002]	[–0.005]	[–0.077]
CBS	[+0.229]	[–0.270]	[+0.086]	[–0.028]	[+0.002]	[–0.005]	[+0.014]

$$\Delta E_{strain} = E_{CCSDT(Q)/CBS} + \Delta E_{core} + \Delta E_{rel} + \Delta E_{DBOC}$$

$$\Delta E_{strain} = 0.014 - 0.070 + 0.010 - 0.001 = \mathbf{-0.05}$$

Table S16. Focal point analysis for the HF•••HBr complex with further additive corrections at the CCSD(T) level of theory with units in kcal mol^{-1}. Bracketed values indicate the extrapolated energies or additive corrections. Additive corrections are indicated by the δ which represents the incremental change from the previous energy.

	RHF	+δMP2	+δCCSD	+δ(T)	+δT	+δ(Q)	NET
aVDZ	–1.119	–0.981	+0.262	–0.172	–0.001	–0.008	[–2.018]
aVTZ	–1.207	–1.399	+0.282	–0.254	+0.001	–0.013	[–2.589]
aVQZ	–1.211	–1.517	+0.291	–0.271	[+0.001]	[–0.013]	[–2.719]
aV5Z	–1.209	–1.559	+0.302	–0.277	[+0.001]	[–0.013]	[–2.755]
CBS	[–1.208]	[–1.604]	[+0.314]	[–0.282]	[+0.001]	[–0.013]	[–2.792]

$$\Delta E_{int}^{CP} = E_{CCSDT(Q)/CBS} + \Delta E_{core}$$

$$\Delta E_{int}^{CP} = -2.792 - 0.030 = \mathbf{-2.82}$$

	RHF	+δMP2	+δCCSD	+δ(T)	+δT	+δ(Q)	NET
aVDZ	+0.022	–0.853	–0.034	–0.113	–0.003	+0.000	[–0.980]
aVTZ	+0.168	–0.674	+0.046	–0.059	+0.003	–0.005	[–0.522]
aVQZ	+0.208	–0.433	+0.104	–0.040	[+0.003]	[–0.005]	[–0.163]
aV5Z	+0.223	–0.331	+0.087	–0.032	[+0.003]	[–0.005]	[–0.056]
CBS	[+0.229]	[–0.224]	[+0.069]	[–0.024]	[+0.003]	[–0.005]	[+0.048]

$$\Delta E_{strain} = E_{CCSDT(Q)/CBS} + \Delta E_{core}$$

$$\Delta E_{strain} = 0.048 - 0.245 = \mathbf{-0.20}$$

Table S17. Focal point analysis for the NH_4^+•••NH_3 complex with further additive corrections at the CCSD(T) level of theory with units in kcal mol^{-1}. Bracketed values indicate the extrapolated energies or additive corrections. Additive corrections are indicated by the δ which represents the incremental change from the previous energy.

	RHF	+δMP2	+δCCSD	+δ(T)	+δT	+δ(Q)	NET
aVDZ	–25.456	–2.681	+1.138	–0.441	+0.007	–0.028	[–27.461]
aVTZ	–25.706	–3.639	+1.037	–0.566	+0.017	–0.037	[–28.894]
aVQZ	–25.725	–4.009	+1.049	–0.599	[+0.017]	[–0.037]	[–29.304]
aV5Z	–25.736	–4.136	+1.075	–0.608	[+0.017]	[–0.037]	[–29.424]
CBS	[–25.743]	[–4.269]	[+1.104]	[–0.618]	[+0.017]	[–0.037]	[–29.545]

$$\Delta E_{\text{int}}^{\text{CP}} = E_{\text{CCSDT(Q)/CBS}} + \Delta E_{\text{core}} + \Delta E_{\text{rel}} + \Delta E_{\text{DBOC}}$$

$$\Delta E_{\text{int}}^{\text{CP}} = -29.545 - 0.100 - 0.003 - 0.017 = \mathbf{-29.66}$$

	RHF	+δMP2	+δCCSD	+δ(T)	+δT	+δ(Q)	NET
aVDZ	+3.694	–2.505	+0.043	–0.261	+0.000	–0.007	[+0.963]
aVTZ	+4.400	–1.762	+0.084	–0.192	+0.004	–0.016	[+2.519]
aVQZ	+4.497	–1.403	+0.115	–0.177	[+0.004]	[–0.016]	[+3.020]
aV5Z	+4.532	–1.230	+0.083	–0.168	[+0.004]	[–0.016]	[+3.206]
CBS	[+4.547]	[–1.047]	[+0.050]	[–0.158]	[+0.004]	[–0.016]	[+3.380]

$$\Delta E_{\text{strain}} = E_{\text{CCSDT(Q)/CBS}} + \Delta E_{\text{core}} + \Delta E_{\text{rel}} + \Delta E_{\text{DBOC}}$$

$$\Delta E_{\text{strain}} = 3.380 + 0.035 + 0.004 - 0.007 = +\mathbf{3.41}$$

Table S18. Focal point analysis for the $NH_4^+\cdots PH_3$ complex with further additive corrections at the CCSD(T) level of theory with units in kcal mol^{-1}. Bracketed values indicate the extrapolated energies or additive corrections. Additive corrections are indicated by the δ which represents the incremental change from the previous energy.

	RHF	+δMP2	+δCCSD	+δ(T)	+δT	+δ(Q)	NET
aVDZ	–12.590	–2.048	+1.047	–0.388	–0.005	–0.027	[–14.012]
aVTZ	–12.655	–2.736	+0.898	–0.461	+0.013	[–0.027]	[–14.968]
aVQZ	–12.663	–3.009	+0.892	–0.480	[+0.013]	[–0.027]	[–15.275]
aV5Z	–12.663	–3.105	+0.918	–0.487	[+0.013]	[–0.027]	[–15.351]
CBS	[–12.662]	[–3.207]	[+0.946]	[–0.493]	[+0.013]	[–0.027]	[–15.430]

$$\Delta E_{\mathrm{int}}^{\mathrm{CP}} = E_{\mathrm{CCSDT(Q)/CBS}} + \Delta E_{\mathrm{core}} + \Delta E_{\mathrm{rel}} + \Delta E_{\mathrm{DBOC}}$$

$$\Delta E_{\mathrm{int}}^{\mathrm{CP}} = -15.430 + 0.033 + 0.070 - 0.015 = \mathbf{-15.34}$$

	RHF	+δMP2	+δCCSD	+δ(T)	+δT	+δ(Q)	NET
aVDZ	+0.634	–0.680	+0.062	–0.063	+0.001	+0.001	[–0.045]
aVTZ	+0.767	–0.084	+0.112	+0.025	+0.008	[+0.001]	[+0.829]
aVQZ	+0.792	+0.175	+0.103	+0.049	[+0.008]	[+0.001]	[+1.129]
aV5Z	+0.796	+0.272	+0.073	+0.057	[+0.008]	[+0.001]	[+1.207]
CBS	[+0.796]	[+0.374]	[+0.042]	[+0.065]	[+0.008]	[+0.001]	[+1.286]

$$\Delta E_{\mathrm{strain}} = E_{\mathrm{CCSDT(Q)/CBS}} + \Delta E_{\mathrm{core}} + \Delta E_{\mathrm{rel}} + \Delta E_{\mathrm{DBOC}}$$

$$\Delta E_{\mathrm{strain}} = 1.286 - 0.082 + 0.032 - 0.001 = +\mathbf{1.23}$$

Table S19. Focal point analysis for the NH_4^+•••AsH_3 complex with further additive corrections at the CCSD(T) level of theory with units in kcal mol^{-1}. Bracketed values indicate the extrapolated energies or additive corrections. Additive corrections are indicated by the δ which represents the incremental change from the previous energy.

	RHF	+δMP2	+δCCSD	+δ(T)	+δT	+δ(Q)	NET
aVDZ	–10.368	–2.097	+0.874	–0.366	–0.004	–0.026	[–11.987]
aVTZ	–10.311	–2.794	+0.745	–0.446	+0.011	[–0.026]	[–12.820]
aVQZ	–10.282	–3.058	+0.747	–0.468	[+0.011]	[–0.026]	[–13.076]
aV5Z	–10.263	–3.156	+0.773	–0.475	[+0.011]	[–0.026]	[–13.135]
CBS	[–10.254]	[–3.258]	[+0.801]	[–0.482]	[+0.011]	[–0.026]	[–13.207]

$$\Delta E_{\text{int}}^{\text{CP}} = E_{\text{CCSDT(Q)/CBS}} + \Delta E_{\text{core}}$$

$$\Delta E_{\text{int}}^{\text{CP}} = -13.207 + 0.094 = \mathbf{-13.11}$$

	RHF	+δMP2	+δCCSD	+δ(T)	+δT	+δ(Q)	NET
aVDZ	+0.476	–0.496	+0.142	–0.045	+0.001	+0.002	[+0.081]
aVTZ	+0.556	+0.103	+0.183	+0.048	+0.010	[+0.002]	[+0.902]
aVQZ	+0.544	+0.309	+0.167	+0.069	[+0.010]	[+0.002]	[+1.103]
aV5Z	+0.543	+0.401	+0.138	+0.077	[+0.010]	[+0.002]	[+1.172]
CBS	[+0.543]	[+0.497]	[+0.107]	[+0.085]	[+0.010]	[+0.002]	[+1.245]

$$\Delta E_{\text{strain}} = E_{\text{CCSDT(Q)/CBS}} + \Delta E_{\text{core}}$$

$$\Delta E_{\text{strain}} = 1.245 - 0.421 = +\mathbf{0.82}$$

Table S20. Focal point analysis for the H_3O^+•••H_2O complex with further additive corrections at the CCSD(T) level of theory with units in kcal mol^{-1}. Bracketed values indicate the extrapolated energies or additive corrections. Additive corrections are indicated by the δ which represents the incremental change from the previous energy.

	RHF	+δMP2	+δCCSD	+δ(T)	+δT	+δ(Q)	NET
aVDZ	–49.678	+0.665	+0.834	–0.155	+0.034	+0.018	[–48.282]
aVTZ	–50.772	–0.504	+0.546	–0.213	+0.018	[+0.018]	[–50.906]
aVQZ	–50.868	–1.044	+0.487	–0.239	[+0.018]	[+0.018]	[–51.627]
aV5Z	–50.897	–1.253	+0.501	–0.247	[+0.018]	[+0.018]	[–51.860]
CBS	[–50.909]	[–1.472]	[+0.516]	[–0.256]	[+0.018]	[+0.018]	[–52.085]

$$\Delta E_{\text{int}}^{\text{CP}} = E_{\text{CCSDT(Q)/CBS}} + \Delta E_{\text{core}} + \Delta E_{\text{rel}} + \Delta E_{\text{DBOC}}$$

$$\Delta E_{\text{int}}^{\text{CP}} = -52.085 - 0.142 + 0.018 - 0.009 = \mathbf{-52.22}$$

	RHF	+δMP2	+δCCSD	+δ(T)	+δT	+δ(Q)	NET
aVDZ	+20.707	–5.718	+0.413	–0.549	–0.010	–0.038	[+14.806]
aVTZ	+21.414	–4.690	+0.651	–0.512	+0.010	–0.050	[+16.822]
aVQZ	+21.687	–4.198	+0.760	–0.501	[+0.010]	[–0.050]	[+17.707]
aV5Z	+21.762	–3.882	+0.735	–0.488	[+0.010]	[–0.050]	[+18.087]
CBS	[+21.790]	[–3.550]	[+0.709]	[–0.475]	[+0.010]	[–0.050]	[+18.434]

$$\Delta E_{\text{strain}} = E_{\text{CCSDT(Q)/CBS}} + \Delta E_{\text{core}} + \Delta E_{\text{rel}} + \Delta E_{\text{DBOC}}$$

$$\Delta E_{\text{strain}} = 18.434 + 0.029 + 0.025 - 0.020 = +\mathbf{18.47}$$

Table S21. Focal point analysis for the H_3O^+•••H_2S complex with further additive corrections at the CCSD(T) level of theory with units in kcal mol^{-1}. Bracketed values indicate the extrapolated energies or additive corrections. Additive corrections are indicated by the δ which represents the incremental change from the previous energy.

	RHF	+δMP2	+δCCSD	+δ(T)	+δT	+δ(Q)	NET
aVDZ	–25.681	–3.781	+1.541	–0.709	–0.002	–0.044	[–28.675]
aVTZ	–25.736	–4.829	+1.563	–0.831	+0.025	[–0.044]	[–29.851]
aVQZ	–25.773	–5.272	+1.552	–0.866	[+0.025]	[–0.044]	[–30.378]
aV5Z	–25.788	–5.430	+1.574	–0.878	[+0.025]	[–0.044]	[–30.540]
CBS	[–25.795]	[–5.595]	[+1.598]	[–0.890]	[+0.025]	[–0.044]	[–30.702]

$$\Delta E_{int}^{CP} = E_{CCSDT(Q)/CBS} + \Delta E_{core} + \Delta E_{rel} + \Delta E_{DBOC}$$

$$\Delta E_{int}^{CP} = -30.702 - 0.037 - 0.046 + 0.013 = \mathbf{-30.77}$$

	RHF	+δMP2	+δCCSD	+δ(T)	+δT	+δ(Q)	NET
aVDZ	+8.097	–3.760	+0.143	–0.351	–0.013	–0.031	[+4.086]
aVTZ	+8.618	–3.087	+0.400	–0.327	+0.007	[–0.031]	[+5.581]
aVQZ	+8.817	–2.592	+0.474	–0.299	[+0.007]	[–0.031]	[+6.377]
aV5Z	+8.867	–2.385	+0.440	–0.288	[+0.007]	[–0.031]	[+6.610]
CBS	[+8.883]	[–2.168]	[+0.404]	[–0.276]	[+0.007]	[–0.031]	[+6.819]

$$\Delta E_{strain} = E_{CCSDT(Q)/CBS} + \Delta E_{core} + \Delta E_{rel} + \Delta E_{DBOC}$$

$$\Delta E_{strain} = 6.819 - 0.122 + 0.014 - 0.010 = +\mathbf{6.70}$$

Table S22. Focal point analysis for the H_3O^+•••H_2Se complex with further additive corrections at the CCSD(T) level of theory with units in kcal mol^{-1}. Bracketed values indicate the extrapolated energies or additive corrections. Additive corrections are indicated by the δ which represents the incremental change from the previous energy.

	RHF	+δMP2	+δCCSD	+δ(T)	+δT	+δ(Q)	NET
aVDZ	–24.137	–4.352	+1.389	–0.738	–0.003	–0.050	[–27.889]
aVTZ	–23.984	–5.418	+1.457	–0.878	+0.023	[–0.050]	[–28.849]
aVQZ	–24.016	–5.812	+1.470	–0.914	[+0.023]	[–0.050]	[–29.299]
aV5Z	–24.016	–5.958	+1.494	–0.927	[+0.023]	[–0.050]	[–29.433]
CBS	[–24.013]	[–6.111]	[+1.519]	[–0.940]	[+0.023]	[–0.050]	[–29.572]

$$\Delta E_{\text{int}}^{\text{CP}} = E_{\text{CCSDT(Q)/CBS}} + \Delta E_{\text{core}}$$

$$\Delta E_{\text{int}}^{\text{CP}} = -29.572 - 0.035 = \mathbf{-29.61}$$

	RHF	+δMP2	+δCCSD	+δ(T)	+δT	+δ(Q)	NET
aVDZ	+7.327	–3.705	+0.104	–0.328	–0.012	–0.030	[+3.358]
aVTZ	+7.728	–2.935	+0.385	–0.306	+0.008	[–0.030]	[+4.850]
aVQZ	+7.889	–2.504	+0.463	–0.281	[+0.008]	[–0.030]	[+5.545]
aV5Z	+7.926	–2.292	+0.424	–0.270	[+0.008]	[–0.030]	[+5.765]
CBS	[+7.936]	[–2.070]	[+0.382]	[–0.258]	[+0.008]	[–0.030]	[+5.969]

$$\Delta E_{\text{strain}} = E_{\text{CCSDT(Q)/CBS}} + \Delta E_{\text{core}}$$

$$\Delta E_{\text{strain}} = 5.969 - 0.423 = +\mathbf{5.55}$$

Table S23. Focal point analysis for the H_2F^+•••HF complex with further additive corrections at the CCSD(T) level of theory with units in kcal mol^{-1}. Bracketed values indicate the extrapolated energies or additive corrections. Additive corrections are indicated by the δ which represents the incremental change from the previous energy.

	RHF	+δMP2	+δCCSD	+δ(T)	+δT	+δ(Q)	NET
aVDZ	–42.846	+1.782	+0.134	–0.107	+0.052	+0.007	[–40.978]
aVTZ	–44.696	+1.005	–0.165	–0.073	+0.015	–0.000	[–43.915]
aVQZ	–44.887	+0.609	–0.250	–0.083	[+0.015]	[–0.000]	[–44.597]
aV5Z	–44.933	+0.445	–0.254	–0.086	[+0.015]	[–0.000]	[–44.812]
CBS	[–44.947]	[+0.273]	[–0.257]	[–0.090]	[+0.015]	[–0.000]	[–45.005]

$$\Delta E_{\text{int}}^{\text{CP}} = E_{\text{CCSDT(Q)/CBS}} + \Delta E_{\text{core}} + \Delta E_{\text{rel}} + \Delta E_{\text{DBOC}}$$

$$\Delta E_{\text{int}}^{\text{CP}} = -45.005 - 0.097 + 0.044 - 0.003 = \mathbf{-45.06}$$

	RHF	+δMP2	+δCCSD	+δ(T)	+δT	+δ(Q)	NET
aVDZ	+14.037	–5.634	+0.602	–0.533	–0.019	–0.028	[+8.426]
aVTZ	+15.092	–4.967	+0.838	–0.530	+0.005	–0.041	[+10.398]
aVQZ	+15.445	–4.462	+0.981	–0.525	[+0.005]	[–0.041]	[+11.403]
aV5Z	+15.626	–4.175	+0.992	–0.521	[+0.005]	[–0.041]	[+11.886]
CBS	[+15.735]	[–3.875]	[+1.004]	[–0.517]	[+0.005]	[–0.041]	[+12.311]

$$\Delta E_{\text{strain}} = E_{\text{CCSDT(Q)/CBS}} + \Delta E_{\text{core}} + \Delta E_{\text{rel}} + \Delta E_{\text{DBOC}}$$

$$\Delta E_{\text{strain}} = 12.311 + 0.023 + 0.001 - 0.014 = +\mathbf{12.32}$$

Table S24. Focal point analysis for the H_2F^+•••HCl complex with further additive corrections at the CCSD(T) level of theory with units in kcal mol^{-1}. Bracketed values indicate the extrapolated energies or additive corrections. Additive corrections are indicated by the δ which represents the incremental change from the previous energy.

	RHF	+δMP2	+δCCSD	+δ(T)	+δT	+δ(Q)	NET
aVDZ	–76.248	+1.896	–0.894	+0.052	+0.003	+0.020	[–75.171]
aVTZ	–77.571	+0.956	–0.858	+0.174	–0.020	+0.011	[–77.309]
aVQZ	–77.691	+0.600	–0.900	+0.165	[–0.020]	[+0.011]	[–77.834]
aV5Z	–77.749	+0.481	–0.933	+0.167	[–0.020]	[+0.011]	[–78.043]
CBS	[–77.779]	[+0.356]	[–0.968]	[+0.169]	[–0.020]	[+0.011]	[–78.231]

$$\Delta E_{\text{int}}^{\text{CP}} = E_{\text{CCSDT(Q)/CBS}} + \Delta E_{\text{core}} + \Delta E_{\text{rel}} + \Delta E_{\text{DBOC}}$$

$$\Delta E_{\text{int}}^{\text{CP}} = -78.231 - 0.028 - 0.056 + 0.053 = \mathbf{-78.26}$$

	RHF	+δMP2	+δCCSD	+δ(T)	+δT	+δ(Q)	NET
aVDZ	+46.120	–8.057	+1.112	–0.965	–0.043	–0.040	[+38.126]
aVTZ	+48.614	–7.282	+1.738	–1.045	+0.015	–0.056	[+41.984]
aVQZ	+48.826	–6.582	+1.986	–1.015	[+0.015]	[–0.056]	[+43.175]
aV5Z	+48.957	–6.335	+1.994	–1.006	[+0.015]	[–0.056]	[+43.570]
CBS	[+49.031]	[–6.076]	[+2.002]	[–0.995]	[+0.015]	[–0.056]	[+43.921]

$$\Delta E_{\text{strain}} = E_{\text{CCSDT(Q)/CBS}} + \Delta E_{\text{core}} + \Delta E_{\text{rel}} + \Delta E_{\text{DBOC}}$$

$$\Delta E_{\text{strain}} = 43.921 - 0.167 + 0.012 - 0.027 = +\mathbf{43.74}$$

Table S25. Focal point analysis for the H_2F^+•••HBr complex with further additive corrections at the CCSD(T) level of theory with units in kcal mol^{-1}. Bracketed values indicate the extrapolated energies or additive corrections. Additive corrections are indicated by the δ which represents the incremental change from the previous energy.

	RHF	+δMP2	+δCCSD	+δ(T)	+δT	+δ(Q)	NET
aVDZ	–86.800	+1.022	–1.340	+0.090	+0.003	+0.012	[–87.012]
aVTZ	–87.869	+0.182	–1.231	+0.229	–0.020	+0.007	[–88.702]
aVQZ	–88.023	–0.056	–1.249	+0.223	[–0.020]	[+0.007]	[–89.118]
aV5Z	–88.056	–0.127	–1.299	+0.225	[–0.020]	[+0.007]	[–89.269]
CBS	[–88.064]	[–0.201]	[–1.352]	[+0.228]	[–0.020]	[+0.007]	[–89.402]

$$\Delta E_{\text{int}}^{\text{CP}} = E_{\text{CCSDT(Q)/CBS}} + \Delta E_{\text{core}}$$

$$\Delta E_{\text{int}}^{\text{CP}} = -89.402 + 0.129 = \mathbf{-89.27}$$

	RHF	+δMP2	+δCCSD	+δ(T)	+δT	+δ(Q)	NET
aVDZ	+55.333	–8.135	+1.241	–1.020	–0.043	–0.037	[+47.339]
aVTZ	+57.982	–7.402	+1.902	–1.124	+0.020	–0.056	[+51.321]
aVQZ	+58.161	–6.694	+2.194	–1.092	[+0.020]	[–0.056]	[+52.534]
aV5Z	+58.272	–6.419	+2.192	–1.081	[+0.020]	[–0.056]	[+52.929]
CBS	[+58.334]	[–6.130]	[+2.189]	[–1.069]	[+0.020]	[–0.056]	[+53.290]

$$\Delta E_{\text{strain}} = E_{\text{CCSDT(Q)/CBS}} + \Delta E_{\text{core}}$$

$$\Delta E_{\text{strain}} = 53.290 - 0.555 = +\mathbf{52.73}$$

Table S26. Focal point analysis for the NH_3•••NH_2^- complex with further additive corrections at the CCSD(T) level of theory with units in kcal mol^{-1}. Bracketed values indicate the extrapolated energies or additive corrections. Additive corrections are indicated by the δ which represents the incremental change from the previous energy.

	RHF	+δMP2	+δCCSD	+δ(T)	+δT	+δ(Q)	NET
aVDZ	–11.521	–1.793	+0.476	–0.330	–0.007	+0.009	[–13.166]
aVTZ	–11.635	–2.536	+0.361	–0.432	–0.002	[+0.009]	[–14.237]
aVQZ	–11.632	–2.784	+0.350	–0.451	[–0.002]	[+0.009]	[–14.511]
aV5Z	–11.615	–2.848	+0.353	–0.447	[–0.002]	[+0.009]	[–14.549]
CBS	[–11.602]	[–2.915]	[+0.357]	[–0.442]	[–0.002]	[+0.009]	[–14.596]

$$\Delta E_{int}^{CP} = E_{CCSDT(Q)/CBS} + \Delta E_{core} + \Delta E_{rel} + \Delta E_{DBOC}$$

$$\Delta E_{int}^{CP} = -14.596 - 0.066 + 0.005 - 0.016 = \mathbf{-14.67}$$

	RHF	+δMP2	+δCCSD	+δ(T)	+δT	+δ(Q)	NET
aVDZ	+1.005	–2.067	+0.170	–0.337	+0.001	–0.033	[–1.260]
aVTZ	+1.503	–1.469	+0.208	–0.234	+0.011	[–0.033]	[–0.013]
aVQZ	+1.593	–1.107	+0.194	–0.187	[+0.011]	[–0.033]	[+0.470]
aV5Z	+1.636	–0.899	+0.134	–0.160	[+0.011]	[–0.033]	[+0.690]
CBS	[+1.659]	[–0.680]	[+0.071]	[–0.130]	[+0.011]	[–0.033]	[+0.898]

$$\Delta E_{strain} = E_{CCSDT(Q)/CBS} + \Delta E_{core} + \Delta E_{rel} + \Delta E_{DBOC}$$

$$\Delta E_{strain} = 0.898 + 0.024 - 0.001 - 0.001 = +\mathbf{0.92}$$

Table S27. Focal point analysis for the NH_3•••PH_2^-complex with further additive corrections at the CCSD(T) level of theory with units in kcal mol^{-1}. Bracketed values indicate the extrapolated energies or additive corrections. Additive corrections are indicated by the δ which represents the incremental change from the previous energy.

	RHF	+δMP2	+δCCSD	+δ(T)	+δT	+δ(Q)	NET
aVDZ	–4.639	–2.378	+0.578	–0.395	–0.005	–0.040	[–6.880]
aVTZ	–4.638	–2.970	+0.609	–0.504	+0.011	–0.051	[–7.542]
aVQZ	–4.636	–3.157	+0.642	–0.531	[+0.011]	[–0.051]	[–7.723]
aV5Z	–4.635	–3.228	+0.668	–0.539	[+0.011]	[–0.051]	[–7.773]
CBS	[–4.634]	[–3.302]	[+0.697]	[–0.548]	[+0.011]	[–0.051]	[–7.827]

$$\Delta E_{\text{int}}^{\text{CP}} = E_{\text{CCSDT(Q)/CBS}} + \Delta E_{\text{core}} + \Delta E_{\text{rel}} + \Delta E_{\text{DBOC}}$$

$$\Delta E_{\text{int}}^{\text{CP}} = -7.827 - 0.038 + 0.003 - 0.007 = \mathbf{-7.87}$$

	RHF	+δMP2	+δCCSD	+δ(T)	+δT	+δ(Q)	NET
aVDZ	+0.396	–1.248	+0.087	–0.184	–0.002	–0.011	[–0.963]
aVTZ	+0.669	–0.906	+0.159	–0.114	+0.008	–0.010	[–0.195]
aVQZ	+0.720	–0.644	+0.134	–0.087	[+0.008]	[–0.010]	[+0.121]
aV5Z	+0.734	–0.539	+0.095	–0.078	[+0.008]	[–0.010]	[+0.210]
CBS	[+0.739]	[–0.428]	[+0.054]	[–0.070]	[+0.008]	[–0.010]	[+0.293]

$$\Delta E_{\text{strain}} = E_{\text{CCSDT(Q)/CBS}} + \Delta E_{\text{core}} + \Delta E_{\text{rel}} + \Delta E_{\text{DBOC}}$$

$$\Delta E_{\text{strain}} = 0.293 - 0.065 - 0.008 + 0.002 = +\mathbf{0.22}$$

Table S28. Focal point analysis for the NH_3•••AsH_2^-complex with further additive corrections at the CCSD(T) level of theory with units in kcal mol^{-1}. Bracketed values indicate the extrapolated energies or additive corrections. Additive corrections are indicated by the *δ* which represents the incremental change from the previous energy.

	RHF	+*δ*MP2	+*δ*CCSD	+*δ*(T)	+*δ*T	+*δ*(Q)	NET
aVDZ	–4.264	–2.244	+0.492	–0.373	–0.008	–0.036	[–6.434]
aVTZ	–4.260	–2.787	+0.530	–0.473	+0.006	[–0.036]	[–7.022]
aVQZ	–4.252	–2.958	+0.559	–0.499	[+0.006]	[–0.036]	[–7.181]
aV5Z	–4.254	–3.022	+0.583	–0.506	[+0.006]	[–0.036]	[–7.230]
CBS	[–4.255]	[–3.089]	[+0.608]	[–0.514]	[+0.006]	[–0.036]	[–7.281]

$$\Delta E_{\text{int}}^{\text{CP}} = E_{\text{CCSDT(Q)/CBS}} + \Delta E_{\text{core}}$$

$$\Delta E_{\text{int}}^{\text{CP}} = -7.281 - 0.093 = \mathbf{-7.37}$$

	RHF	+*δ*MP2	+*δ*CCSD	+*δ*(T)	+*δ*T	+*δ*(Q)	NET
aVDZ	+0.456	–1.163	+0.058	–0.157	–0.002	–0.009	[–0.817]
aVTZ	+0.703	–0.827	+0.130	–0.103	+0.007	[–0.009]	[–0.099]
aVQZ	+0.749	–0.626	+0.113	–0.085	[+0.007]	[–0.009]	[+0.148]
aV5Z	+0.759	–0.529	+0.077	–0.077	[+0.007]	[–0.009]	[+0.228]
CBS	[+0.762]	[–0.426]	[+0.040]	[–0.070]	[+0.007]	[–0.009]	[+0.303]

$$\Delta E_{\text{strain}} = E_{\text{CCSDT(Q)/CBS}} + \Delta E_{\text{core}}$$

$$\Delta E_{\text{strain}} = 0.303 - 0.238 = +\mathbf{0.07}$$

Table S29. Focal point analysis for the $H_2O{\cdots}OH^-$ complex with further additive corrections at the CCSD(T) level of theory with units in kcal mol^{-1}. Bracketed values indicate the extrapolated energies or additive corrections. Additive corrections are indicated by the δ which represents the incremental change from the previous energy.

	RHF	+δMP2	+δCCSD	+δ(T)	+δT	+δ(Q)	+δQ	NET
aVDZ	–27.818	+0.688	+0.085	–0.028	–4.234	+0.069	–0.032	[–31.270]
aVTZ	–28.304	–0.664	–0.182	–0.133	–4.248	+0.072	[–0.032]	[–33.491]
aVQZ	–28.331	–1.192	–0.249	–0.161	[–4.248]	[+0.072]	[–0.032]	[–34.140]
aV5Z	–28.340	–1.381	–0.237	–0.159	[–4.248]	[+0.072]	[–0.032]	[–34.324]
CBS	[–28.344]	[–1.578]	[–0.224]	[–0.158]	[–4.248]	[+0.072]	[–0.032]	[–34.511]

$$\Delta E_{\text{int}}^{\text{CP}} = E_{\text{CCSDT(Q)/CBS}} + \Delta E_{\text{core}} + \Delta E_{\text{rel}} + \Delta E_{\text{DBOC}}$$

$$\Delta E_{\text{int}}^{\text{CP}} = -34.511 - 0.118 + 0.029 - 0.019 = \mathbf{-34.62}$$

	RHF	+δMP2	+δCCSD	+δ(T)	+δT	+δ(Q)	NET
aVDZ	+5.507	–4.244	+0.289	–0.591	+3.529	–0.077	[+4.414]
aVTZ	+5.859	–3.357	+0.403	–0.434	+3.703	–0.086	[+6.088]
aVQZ	+6.043	–2.831	+0.470	–0.367	[+3.703]	[–0.086]	[+6.931]
aV5Z	+6.090	–2.466	+0.415	–0.328	[+3.703]	[–0.086]	[+7.327]
CBS	[+6.107]	[–2.083]	[+0.357]	[–0.287]	[+3.703]	[–0.086]	[+7.710]

$$\Delta E_{\text{strain}} = E_{\text{CCSDT(Q)/CBS}} + \Delta E_{\text{core}} + \Delta E_{\text{rel}} + \Delta E_{\text{DBOC}}$$

$$\Delta E_{\text{strain}} = 7.710 + 0.029 - 0.003 - 0.004 = +\mathbf{7.73}$$

Table S30. Focal point analysis for the $H_2O\cdots SH^-$ complex with further additive corrections at the CCSD(T) level of theory with units in kcal mol^{-1}. Bracketed values indicate the extrapolated energies or additive corrections. Additive corrections are indicated by the δ which represents the incremental change from the previous energy.

	RHF	+δMP2	+δCCSD	+δ(T)	+δT	+δ(Q)	NET
aVDZ	–12.090	–2.176	+0.737	–0.362	+0.004	–0.030	[–13.917]
aVTZ	–12.129	–3.103	+0.733	–0.527	+0.014	–0.042	[–15.054]
aVQZ	–12.117	–3.419	+0.741	–0.569	[+0.014]	[–0.042]	[–15.392]
aV5Z	–12.119	–3.539	+0.771	–0.582	[+0.014]	[–0.042]	[–15.497]
CBS	[–12.121]	[–3.665]	[+0.803]	[–0.596]	[+0.014]	[–0.042]	[–15.607]

$$\Delta E_{\mathrm{int}}^{\mathrm{CP}} = E_{\mathrm{CCSDT(Q)/CBS}} + \Delta E_{\mathrm{core}} + \Delta E_{\mathrm{rel}} + \Delta E_{\mathrm{DBOC}}$$

$$\Delta E_{\mathrm{int}}^{\mathrm{CP}} = -15.607 - 0.063 + 0.008 - 0.013 = \mathbf{-15.68}$$

	RHF	+δMP2	+δCCSD	+δ(T)	+δT	+δ(Q)	NET
aVDZ	+1.336	–2.080	+0.088	–0.295	–0.002	–0.020	[–0.973]
aVTZ	+1.532	–1.741	+0.237	–0.186	+0.012	–0.022	[–0.167]
aVQZ	+1.599	–1.339	+0.312	–0.149	[+0.012]	[–0.022]	[+0.414]
aV5Z	+1.618	–1.159	+0.270	–0.134	[+0.012]	[–0.022]	[+0.586]
CBS	[+1.624]	[–0.969]	[+0.227]	[–0.119]	[+0.012]	[–0.022]	[+0.753]

$$\Delta E_{\mathrm{strain}} = E_{\mathrm{CCSDT(Q)/CBS}} + \Delta E_{\mathrm{core}} + \Delta E_{\mathrm{rel}} + \Delta E_{\mathrm{DBOC}}$$

$$\Delta E_{\mathrm{strain}} = 0.753 - 0.077 + 0.005 + 0.000 = +\mathbf{0.68}$$

Table S31. Focal point analysis for the $H_2O{\bullet}{\bullet}{\bullet}SeH^-$ complex with further additive corrections at the CCSD(T) level of theory with units in kcal mol^{-1}. Bracketed values indicate the extrapolated energies or additive corrections. Additive corrections are indicated by the δ which represents the incremental change from the previous energy.

	RHF	+δMP2	+δCCSD	+δ(T)	+δT	+δ(Q)	NET
aVDZ	–10.502	–2.141	+0.657	–0.348	+0.004	–0.028	[–12.359]
aVTZ	–10.482	–2.982	+0.662	–0.501	+0.012	–0.038	[–13.329]
aVQZ	–10.471	–3.268	+0.674	–0.538	[+0.012]	[–0.038]	[–13.630]
aV5Z	–10.470	–3.373	+0.702	–0.550	[+0.012]	[–0.038]	[–13.718]
CBS	[–10.471]	[–3.483]	[+0.731]	[–0.562]	[+0.012]	[–0.038]	[–13.812]

$$\Delta E_{\text{int}}^{\text{CP}} = E_{\text{CCSDT(Q)/CBS}} + \Delta E_{\text{core}}$$

$$\Delta E_{\text{int}}^{\text{CP}} = -13.812 - 0.124 = \mathbf{-13.94}$$

	RHF	+δMP2	+δCCSD	+δ(T)	+δT	+δ(Q)	NET
aVDZ	+1.112	–2.018	+0.049	–0.286	–0.003	–0.016	[–1.163]
aVTZ	+1.268	–1.573	+0.218	–0.161	+0.012	–0.019	[–0.253]
aVQZ	+1.320	–1.197	+0.295	–0.129	[+0.012]	[–0.019]	[+0.282]
aV5Z	+1.335	–1.005	+0.244	–0.113	[+0.012]	[–0.019]	[+0.454]
CBS	[+1.340]	[–0.804]	[+0.190]	[–0.097]	[+0.012]	[–0.019]	[+0.623]

$$\Delta E_{\text{strain}} = E_{\text{CCSDT(Q)/CBS}} + \Delta E_{\text{core}}$$

$$\Delta E_{\text{strain}} = 0.623 - 0.279 = +\mathbf{0.34}$$

Table S32. Focal point analysis for the HF•••F^- complex with further additive corrections at the CCSD(T) level of theory with units in kcal mol^{-1}. Bracketed values indicate the extrapolated energies or additive corrections. Additive corrections are indicated by the δ which represents the incremental change from the previous energy.

	RHF	+δMP2	+δCCSD	+δ(T)	+δT	+δ(Q)	NET
aVDZ	–67.033	+6.851	–0.550	+0.566	+0.136	+0.091	[–59.939]
aVTZ	–68.670	+5.445	–1.042	+0.635	+0.038	+0.090	[–63.504]
aVQZ	–68.764	+4.754	–1.213	+0.638	[+0.038]	[+0.090]	[–64.456]
aV5Z	–68.799	+4.477	–1.221	+0.642	[+0.038]	[+0.090]	[–64.773]
CBS	[–68.818]	[+4.187]	[–1.229]	[+0.645]	[+0.038]	[+0.090]	[–65.087]

$$\Delta E_{\text{int}}^{\text{CP}} = E_{\text{CCSDT(Q)/CBS}} + \Delta E_{\text{core}} + \Delta E_{\text{rel}} + \Delta E_{\text{DBOC}}$$

$$\Delta E_{\text{int}}^{\text{CP}} = -65.087 - 0.147 + 0.081 - 0.013 = \mathbf{-65.17}$$

	RHF	+δMP2	+δCCSD	+δ(T)	+δT	+δ(Q)	NET
aVDZ	+26.491	–9.551	+0.683	–1.031	–0.060	–0.093	[+16.439]
aVTZ	+27.421	–8.750	+1.077	–1.060	–0.002	–0.115	[+18.571]
aVQZ	+27.791	–8.035	+1.268	–1.016	[–0.002]	[–0.115]	[+19.891]
aV5Z	+27.899	–7.559	+1.267	–0.996	[–0.002]	[–0.115]	[+20.494]
CBS	[+27.944]	[–7.061]	[+1.265]	[–0.975]	[–0.002]	[–0.115]	[+21.056]

$$\Delta E_{\text{strain}} = E_{\text{CCSDT(Q)/CBS}} + \Delta E_{\text{core}} + \Delta E_{\text{rel}} + \Delta E_{\text{DBOC}}$$

$$\Delta E_{\text{strain}} = 21.056 + 0.068 + 0.0.018 - 0.011 = \mathbf{+21.13}$$

Table S33. Focal point analysis for the HF•••Cl⁻ complex with further additive corrections at the CCSD(T) level of theory with units in kcal mol^{-1}. Bracketed values indicate the extrapolated energies or additive corrections. Additive corrections are indicated by the δ which represents the incremental change from the previous energy.

	RHF	+δMP2	+δCCSD	+δ(T)	+δT	+δ(Q)	NET
aVDZ	–21.856	–1.374	+0.719	–0.272	+0.012	–0.010	[–22.780]
aVTZ	–22.180	–2.438	+0.683	–0.432	+0.008	–0.020	[–24.380]
aVQZ	–22.161	–2.859	+0.655	–0.477	[+0.008]	[–0.020]	[–24.856]
aV5Z	–22.159	–3.021	+0.679	–0.494	[+0.008]	[–0.020]	[–25.007]
CBS	[–22.159]	[–3.191]	[+0.705]	[–0.511]	[+0.008]	[–0.020]	[–25.169]

$$\Delta E_{\text{int}}^{\text{CP}} = E_{\text{CCSDT(Q)/CBS}} + \Delta E_{\text{core}} + \Delta E_{\text{rel}} + \Delta E_{\text{DBOC}}$$

$$\Delta E_{\text{int}}^{\text{CP}} = -25.169 - 0.084 + 0.010 - 0.011 = \mathbf{-25.25}$$

	RHF	+δMP2	+δCCSD	+δ(T)	+δT	+δ(Q)	NET
aVDZ	+2.908	–2.822	+0.141	–0.339	–0.014	–0.023	[–0.148]
aVTZ	+3.110	–2.667	+0.299	–0.286	+0.007	–0.027	[+0.436]
aVQZ	+3.249	–2.224	+0.456	–0.248	[+0.007]	[–0.027]	[+1.213]
aV5Z	+3.284	–2.070	+0.459	–0.236	[+0.007]	[–0.027]	[+1.418]
CBS	[+3.296]	[–1.908]	[+0.464]	[–0.223]	[+0.007]	[–0.027]	[+1.609]

$$\Delta E_{\text{strain}} = E_{\text{CCSDT(Q)/CBS}} + \Delta E_{\text{core}} + \Delta E_{\text{rel}} + \Delta E_{\text{DBOC}}$$

$$\Delta E_{\text{strain}} = 1.609 - 0.108 - 0.006 - 0.003 = +\mathbf{1.49}$$

Table S34. Focal point analysis for the HF•••Br⁻ complex with further additive corrections at the CCSD(T) level of theory with units in kcal mol^{-1}. Bracketed values indicate the extrapolated energies or additive corrections. Additive corrections are indicated by the δ which represents the incremental change from the previous energy.

	RHF	+δMP2	+δCCSD	+δ(T)	+δT	+δ(Q)	NET
aVDZ	–17.924	–1.517	+0.682	–0.274	+0.014	–0.012	[–19.032]
aVTZ	–18.186	–2.550	+0.654	–0.432	+0.009	–0.020	[–20.525]
aVQZ	–18.168	–2.918	+0.638	–0.474	[+0.009]	[–0.020]	[–20.933]
aV5Z	–18.159	–3.060	+0.661	–0.489	[+0.009]	[–0.020]	[–21.058]
CBS	[–18.155]	[–3.209]	[+0.686]	[–0.505]	[+0.009]	[–0.020]	[–21.194]

$$\Delta E_{\mathrm{int}}^{\mathrm{CP}} = E_{\mathrm{CCSDT(Q)/CBS}} + \Delta E_{\mathrm{core}}$$

$$\Delta E_{\mathrm{int}}^{\mathrm{CP}} = -21.194 - 0.153 = \mathbf{-21.35}$$

	RHF	+δMP2	+δCCSD	+δ(T)	+δT	+δ(Q)	NET
aVDZ	+2.128	–2.530	+0.067	–0.320	–0.013	–0.017	[–0.683]
aVTZ	+2.277	–2.378	+0.220	–0.244	+0.008	–0.022	[–0.140]
aVQZ	+2.395	–1.887	+0.413	–0.207	[+0.008]	[–0.022]	[+0.699]
aV5Z	+2.420	–1.658	+0.382	–0.187	[+0.008]	[–0.022]	[+0.942]
CBS	[+2.427]	[–1.417]	[+0.349]	[–0.167]	[+0.008]	[–0.022]	[+1.177]

$$\Delta E_{\mathrm{strain}} = E_{\mathrm{CCSDT(Q)/CBS}} + \Delta E_{\mathrm{core}}$$

$$\Delta E_{\mathrm{strain}} = 1.177 - 0.302 = +\mathbf{0.87}$$

Table S35. Focal point analysis for the H_2CO•••**Am–O** complex with further additive corrections at the CCSD(T) level of theory with units in kcal mol^{-1}. Bracketed values indicate the extrapolated energies or additive corrections. Additive corrections are indicated by the δ which represents the incremental change from the previous energy.

	RHF	+δMP2	+δCCSD	+δ(T)	NET
jul-VDZ	–3.310	–0.935	+0.282	–0.236	[–4.199]
jul-VTZ	–3.281	–1.373	+0.252	–0.293	[–4.694]
jul-VQZ	–3.311	–1.536	+0.254	–0.313	[–4.905]
jul-V5Z	–3.316	–1.601	+0.266	–0.320	[–4.970]
CBS	[–3.316]	[–1.669]	[+0.277]	[–0.327]	[–5.034]

$$\Delta E_{\text{int}}^{\text{CP}} = E_{\text{CCSD(T)/CBS}} + \Delta E_{\text{core}} + \Delta E_{\text{rel}} + \Delta E_{\text{DBOC}}$$

$$\Delta E_{\text{int}}^{\text{CP}} = -5.034 - 0.017 + 0.002 - 0.013 = \mathbf{-5.06}$$

	RHF	+δMP2	+δCCSD	+δ(T)	NET
jul-VDZ	+1.149	+0.902	–0.032	+0.124	[+2.143]
jul-VTZ	+0.658	+0.329	–0.023	–0.017	[+0.948]
jul-VQZ	+0.582	–0.090	–0.020	–0.057	[+0.415]
jul-V5Z	+0.564	–0.239	+0.008	–0.071	[+0.262]
CBS	[+0.559]	[–0.396]	[+0.038]	[–0.086]	[+0.115]

$$\Delta E_{\text{strain}} = E_{\text{CCSD(T)/CBS}} + \Delta E_{\text{core}} + \Delta E_{\text{rel}} + \Delta E_{\text{DBOC}}$$

$$\Delta E_{\text{strain}} = 0.115 - 0.007 + 0.001 - 0.001 = +\mathbf{0.11}$$

Table S36. Focal point analysis for the H_2CO•••**Am–S** complex with further additive corrections at the CCSD(T) level of theory with units in kcal mol^{-1}. Bracketed values indicate the extrapolated energies or additive corrections. Additive corrections are indicated by the δ which represents the incremental change from the previous energy.

	RHF	+δMP2	+δCCSD	+δ(T)	NET
jul-VDZ	–3.812	–1.103	+0.391	–0.260	[–4.783]
jul-VTZ	–3.756	–1.562	+0.352	–0.330	[–5.297]
jul-VQZ	–3.786	–1.738	+0.349	–0.354	[–5.529]
jul-V5Z	–3.790	–1.809	+0.362	–0.362	[–5.600]
CBS	[–3.790]	[–1.884]	[+0.375]	[–0.370]	[–5.669]

$$\Delta E_{\text{int}}^{\text{CP}} = E_{\text{CCSD(T)/CBS}} + \Delta E_{\text{core}} + \Delta E_{\text{rel}} + \Delta E_{\text{DBOC}}$$

$$\Delta E_{\text{int}}^{\text{CP}} = -5.669 - 0.016 + 0.004 - 0.014 = \mathbf{-5.69}$$

	RHF	+δMP2	+δCCSD	+δ(T)	NET
jul-VDZ	+0.954	+1.215	–0.061	+0.157	[+2.265]
jul-VTZ	+0.508	+0.591	–0.084	+0.015	[+1.030]
jul-VQZ	+0.428	+0.128	–0.085	–0.031	[+0.441]
jul-V5Z	+0.415	–0.039	–0.049	–0.047	[+0.280]
CBS	[+0.414]	[–0.214]	[–0.012]	[–0.064]	[+0.123]

$$\Delta E_{\text{strain}} = E_{\text{CCSD(T)/CBS}} + \Delta E_{\text{core}} + \Delta E_{\text{rel}} + \Delta E_{\text{DBOC}}$$

$$\Delta E_{\text{strain}} = 0.123 - 0.027 + 0.005 - 0.001 = +\mathbf{0.10}$$

Table S37. Focal point analysis for the H_2CO•••**Ur–O** complex in C_1 with further additive corrections at the CCSD(T) level of theory with units in kcal mol^{-1}. Bracketed values indicate the extrapolated energies or additive corrections. Additive corrections are indicated by the δ which represents the incremental change from the previous energy.

	RHF	+δMP2	+δCCSD	+δ(T)	NET
jul-VDZ	–4.276	–0.955	+0.290	–0.217	[–5.158]
jul-VTZ	–4.234	–1.293	+0.247	–0.269	[–5.549]
jul-VQZ	–4.247	–1.443	+0.249	–0.285	[–5.726]
jul-V5Z	–4.258	–1.499	+0.261	–0.290	[–5.787]
CBS	[–4.265]	[–1.557]	[+0.272]	[–0.296]	[–5.845]

$$\Delta E_{\text{int}}^{\text{CP}} = E_{\text{CCSD(T)/CBS}} + \Delta E_{\text{core}} + \Delta E_{\text{rel}} + \Delta E_{\text{DBOC}}$$

$$\Delta E_{\text{int}}^{\text{CP}} = -5.845 - 0.016 + 0.008 - 0.014 = \mathbf{-5.87}$$

	RHF	+δMP2	+δCCSD	+δ(T)	NET
jul-VDZ	+1.248	+1.716	+0.031	+0.267	[+3.262]
jul-VTZ	+0.527	+1.027	–0.001	+0.077	[+1.630]
jul-VQZ	+0.407	+0.348	–0.009	+0.021	[+0.766]
jul-V5Z	+0.382	+0.138	+0.028	+0.003	[+0.551]
CBS	[+0.376]	[–0.082]	[+0.067]	[–0.016]	[+0.345]

$$\Delta E_{\text{strain}} = E_{\text{CCSD(T)/CBS}} + \Delta E_{\text{core}} + \Delta E_{\text{rel}} + \Delta E_{\text{DBOC}}$$

$$\Delta E_{\text{strain}} = 0.345 - 0.052 + 0.017 - 0.003 = +\mathbf{0.31}$$

Table S38. Focal point analysis for the H_2CO•••**Ur–O** complex in C_{2v} with further additive corrections at the CCSD(T) level of theory with units in kcal mol^{-1}. Bracketed values indicate the extrapolated energies or additive corrections. Additive corrections are indicated by the δ which represents the incremental change from the previous energy.

	RHF	+δMP2	+δCCSD	+δ(T)	NET
jul-VDZ	–4.888	–0.862	+0.275	–0.214	[–5.688]
jul-VTZ	–4.840	–1.215	+0.223	–0.265	[–6.097]
jul-VQZ	–4.856	–1.373	+0.223	–0.282	[–6.287]
jul-V5Z	–4.868	–1.432	+0.235	–0.287	[–6.352]
CBS	[–4.874]	[–1.494]	[+0.247]	[–0.293]	[–6.414]

$$\Delta E_{int}^{CP} = E_{CCSD(T)/CBS} + \Delta E_{core} + \Delta E_{rel} + \Delta E_{DBOC}$$

$$\Delta E_{int}^{CP} = -6.414 - 0.017 + 0.009 - 0.015 = \mathbf{-6.44}$$

	RHF	+δMP2	+δCCSD	+δ(T)	NET
jul-VDZ	+1.274	+1.524	–0.071	+0.219	[+2.945]
jul-VTZ	+0.555	+0.706	–0.058	+0.029	[+1.231]
jul-VQZ	+0.467	+0.086	–0.052	–0.029	[+0.472]
jul-V5Z	+0.445	–0.108	–0.016	–0.047	[+0.274]
CBS	[+0.437]	[–0.312]	[+0.022]	[–0.065]	[+0.082]

$$\Delta E_{strain} = E_{CCSD(T)/CBS} + \Delta E_{core} + \Delta E_{rel} + \Delta E_{DBOC}$$

$$\Delta E_{strain} = 0.082 - 0.024 + 0.048 - 0.001 = +\mathbf{0.10}$$

Table S39. Focal point analysis for the H_2CO•••**Ur–S** complex in C_1 with further additive corrections at the CCSD(T) level of theory with units in kcal mol^{-1}. Bracketed values indicate the extrapolated energies or additive corrections. Additive corrections are indicated by the δ which represents the incremental change from the previous energy.

	RHF	+δMP2	+δCCSD	+δ(T)	NET
jul-VDZ	–5.656	–0.838	+0.321	–0.211	[–6.384]
jul-VTZ	–5.591	–1.193	+0.251	–0.272	[–6.806]
jul-VQZ	–5.606	–1.366	+0.242	–0.291	[–7.021]
jul-V5Z	–5.617	–1.431	+0.254	–0.297	[–7.091]
CBS	[–5.623]	[–1.499]	[+0.268]	[–0.303]	[–7.158]

$$\Delta E_{\text{int}}^{\text{CP}} = E_{\text{CCSD(T)/CBS}} + \Delta E_{\text{core}} + \Delta E_{\text{rel}} + \Delta E_{\text{DBOC}}$$

$$\Delta E_{\text{int}}^{\text{CP}} = -7.158 - 0.016 + 0.015 - 0.017 = \mathbf{-7.18}$$

	RHF	+δMP2	+δCCSD	+δ(T)	NET
jul-VDZ	+0.678	+2.614	–0.119	+0.431	[+3.605]
jul-VTZ	–0.120	+1.853	–0.222	+0.235	[+1.747]
jul-VQZ	–0.249	+1.099	–0.246	+0.169	[+0.773]
jul-V5Z	–0.270	+0.857	–0.198	+0.148	[+0.536]
CBS	[–0.274]	[+0.602]	[–0.147]	[+0.125]	[+0.307]

$$\Delta E_{\text{strain}} = E_{\text{CCSD(T)/CBS}} + \Delta E_{\text{core}} + \Delta E_{\text{rel}} + \Delta E_{\text{DBOC}}$$

$$\Delta E_{\text{strain}} = 0.345 - 0.080 + 0.031 - 0.007 = +\mathbf{0.25}$$

Table S40. Focal point analysis for the H_2CO•••**Ur–S** complex in C_{2v} with further additive corrections at the CCSD(T) level of theory with units in kcal mol^{-1}. Bracketed values indicate the extrapolated energies or additive corrections. Additive corrections are indicated by the δ which represents the incremental change from the previous energy.

	RHF	+δMP2	+δCCSD	+δ(T)	NET
jul-VDZ	–5.872	–0.813	+0.317	–0.210	[–6.578]
jul-VTZ	–5.805	–1.172	+0.245	–0.271	[–7.004]
jul-VQZ	–5.822	–1.347	+0.235	–0.289	[–7.224]
jul-V5Z	–5.833	–1.413	+0.248	–0.296	[–7.295]
CBS	[–5.839]	[–1.483]	[+0.261]	[–0.302]	[–7.363]

$$\Delta E_{\text{int}}^{\text{CP}} = E_{\text{CCSD(T)/CBS}} + \Delta E_{\text{core}} + \Delta E_{\text{rel}} + \Delta E_{\text{DBOC}}$$

$$\Delta E_{\text{int}}^{\text{CP}} = -7.363 - 0.017 + 0.015 - 0.018 = \mathbf{-7.38}$$

	RHF	+δMP2	+δCCSD	+δ(T)	NET
jul-VDZ	+1.096	+1.876	–0.122	+0.259	[+3.110]
jul-VTZ	+0.398	+1.005	–0.152	+0.065	[+1.315]
jul-VQZ	+0.309	+0.345	–0.152	+0.001	[+0.502]
jul-V5Z	+0.289	+0.126	–0.106	–0.020	[+0.288]
CBS	[+0.283]	[–0.104]	[–0.058]	[–0.042]	[+0.079]

$$\Delta E_{\text{strain}} = E_{\text{CCSD(T)/CBS}} + \Delta E_{\text{core}} + \Delta E_{\text{rel}} + \Delta E_{\text{DBOC}}$$

$$\Delta E_{\text{strain}} = 0.079 - 0.031 + 0.009 - 0.001 = +\mathbf{0.06}$$

Table S41. Focal point analysis for the H_2CO•••**Delt–O** complex with further additive corrections at the CCSD(T) level of theory with units in kcal mol^{-1}. Bracketed values indicate the extrapolated energies or additive corrections. Additive corrections are indicated by the δ which represents the incremental change from the previous energy.

	RHF	+δMP2	+δCCSD	+δ(T)	NET
jul-VDZ	–4.686	–1.367	+0.368	–0.289	–5.974
jul-VTZ	–4.568	–1.756	+0.348	–0.289	–6.333
jul-VQZ	–4.580	–1.910	+0.355	–0.376	–6.511
jul-V5Z	–4.587	–1.968	[+0.361]	[–0.383]	[–6.577]
CBS	[–4.584]	[–2.029]	[+0.367]	[–0.391]	[–6.637]

$$\Delta E_{\text{int}}^{\text{CP}} = E_{\text{CCSD(T)/CBS}} + \Delta E_{\text{core}} + \Delta E_{\text{rel}} + \Delta E_{\text{DBOC}}$$

$$\Delta E_{\text{int}}^{\text{CP}} = -6.637 - 0.005 + 0.003 - 0.013 = \mathbf{-6.65}$$

	RHF	+δMP2	+δCCSD	+δ(T)	NET
jul-VDZ	+0.251	–0.837	+0.141	–0.128	–0.573
jul-VTZ	+0.334	–0.646	+0.139	–0.083	–0.255
jul-VQZ	+0.444	–0.491	+0.156	–0.067	+0.042
jul-V5Z	+0.476	–0.396	[+0.123]	[–0.061]	[+0.141]
CBS	[+0.469]	[–0.298]	[+0.088]	[–0.055]	[+0.204]

$$\Delta E_{\text{strain}} = E_{\text{CCSD(T)/CBS}} + \Delta E_{\text{core}} + \Delta E_{\text{rel}} + \Delta E_{\text{DBOC}}$$

$$\Delta E_{\text{strain}} = 0.204 - 0.081 - 0.004 - 0.000 = \mathbf{0.12}$$

Table S42. Focal point analysis for the H_2CO•••**Delt–S** complex with further additive corrections at the CCSD(T) level of theory with units in kcal mol^{-1}. Bracketed values indicate the extrapolated energies or additive corrections. Additive corrections are indicated by the δ which represents the incremental change from the previous energy.

	RHF	+δMP2	+δCCSD	+δ(T)	NET
jul-VDZ	–5.248	–1.330	+0.375	–0.268	–6.471
jul-VTZ	–5.107	–1.695	+0.347	–0.341	–6.795
jul-VQZ	–5.110	–1.847	+0.348	–0.362	–6.971
jul-V5Z	–5.116	–1.906	[+0.352]	[–0.369]	[–7.039]
CBS	[–5.114]	[–1.967]	[+0.356]	[–0.376]	[–7.101]

$$\Delta E_{\text{int}}^{\text{CP}} = E_{\text{CCSD(T)/CBS}} + \Delta E_{\text{core}} + \Delta E_{\text{rel}} + \Delta E_{\text{DBOC}}$$

$$\Delta E_{\text{int}}^{\text{CP}} = -7.101 - 0.001 + 0.009 - 0.012 = \mathbf{-7.11}$$

	RHF	+δMP2	+δCCSD	+δ(T)	NET
jul-VDZ	+0.101	–0.696	+0.050	–0.102	–0.647
jul-VTZ	+0.228	–0.437	+0.041	–0.046	–0.214
jul-VQZ	+0.344	–0.275	+0.056	–0.029	+0.096
jul-V5Z	+0.377	–0.173	[+0.017]	[–0.023]	[+0.198]
CBS	[+0.370]	[–0.066]	[–0.023]	[–0.017]	[+0.263]

$$\Delta E_{\text{strain}} = E_{\text{CCSD(T)/CBS}} + \Delta E_{\text{core}} + \Delta E_{\text{rel}} + \Delta E_{\text{DBOC}}$$

$$\Delta E_{\text{strain}} = 0.263 - 0.074 - 0.005 - 0.000 = \mathbf{0.18}$$

Table S43. Focal point analysis for the H_2CO•••**Squar–O** complex with further additive corrections at the CCSD(T) level of theory with units in kcal mol^{-1}. Bracketed values indicate the extrapolated energies or additive corrections. Additive corrections are indicated by the δ which represents the incremental change from the previous energy.

	RHF	+δMP2	+δCCSD	+δ(T)	NET
jul-VDZ	–6.722	–1.254	+0.521	–0.387	–7.842
jul-VTZ	–6.617	–1.714	+0.470	–0.446	–8.306
jul-VQZ	–6.647	–1.907	+0.471	–0.464	–8.546
jul-V5Z	–6.656	[–1.975]	[+0.471]	[–0.470]	[–8.630]
CBS	[–6.654]	[–2.047]	[+0.472]	[–0.477]	[–8.706]

$$\Delta E_{\text{int}}^{\text{CP}} = E_{\text{CCSD(T)/CBS}} + \Delta E_{\text{core}} + \Delta E_{\text{rel}} + \Delta E_{\text{DBOC}}$$

$$\Delta E_{\text{int}}^{\text{CP}} = -8.706 - 0.007 + 0.004 - 0.018 = \mathbf{-8.73}$$

	RHF	+δMP2	+δCCSD	+δ(T)	NET
jul-VDZ	+0.372	–1.222	+0.225	–0.188	–0.813
jul-VTZ	+0.586	–0.998	+0.242	–0.140	–0.310
jul-VQZ	+0.707	–0.770	+0.259	–0.120	+0.075
jul-V5Z	+0.746	[–0.689]	[+0.264]	[–0.113]	[+0.208]
CBS	[+0.737]	[–0.604]	[+0.270]	[–0.106]	[+0.297]

$$\Delta E_{\text{strain}} = E_{\text{CCSD(T)/CBS}} + \Delta E_{\text{core}} + \Delta E_{\text{rel}} + \Delta E_{\text{DBOC}}$$

$$\Delta E_{\text{strain}} = 0.297 - 0.080 - 0.001 - 0.001 = \mathbf{0.22}$$

Table S44. Focal point analysis for the H_2CO•••**Squar–S** complex with further additive corrections at the CCSD(T) level of theory with units in kcal mol^{-1}. Bracketed values indicate the extrapolated energies or additive corrections. Additive corrections are indicated by the δ which represents the incremental change from the previous energy.

	RHF	+δMP2	+δCCSD	+δ(T)	NET
jul-VDZ	–7.692	–1.090	+0.564	–0.382	–8.599
jul-VTZ	–7.554	–1.534	+0.481	–0.449	–9.056
jul-VQZ	–7.578	–1.739	+0.468	–0.469	–9.318
jul-V5Z	–7.586	[–1.812]	[+0.464]	[–0.475]	[–9.409]
CBS	[–7.584]	[–1.889]	[+0.459]	[–0.483]	[–9.496]

$$\Delta E_{\text{int}}^{\text{CP}} = E_{\text{CCSD(T)/CBS}} + \Delta E_{\text{core}} + \Delta E_{\text{rel}} + \Delta E_{\text{DBOC}}$$

$$\Delta E_{\text{int}}^{\text{CP}} = -9.496 - 0.000 + 0.014 - 0.019 = \mathbf{-9.50}$$

	RHF	+δMP2	+δCCSD	+δ(T)	NET
jul-VDZ	+0.156	–0.914	+0.121	–0.161	–0.797
jul-VTZ	+0.347	–0.659	+0.124	–0.100	–0.287
jul-VQZ	+0.466	–0.416	+0.137	–0.076	+0.111
jul-V5Z	+0.504	[–0.329]	[+0.141]	[–0.068]	[+0.248]
CBS	[+0.495]	[–0.238]	[+0.146]	[–0.059]	[+0.344]

$$\Delta E_{\text{strain}} = E_{\text{CCSD(T)/CBS}} + \Delta E_{\text{core}} + \Delta E_{\text{rel}} + \Delta E_{\text{DBOC}}$$

$$\Delta E_{\text{strain}} = 0.344 - 0.077 - 0.002 - 0.001 = \mathbf{0.26}$$

Table S45. Final extrapolated hydrogen bond energies (ΔE), strain energies (ΔE_{strain}), and interaction energies (ΔE_{int}, in kcal mol^{-1}) targeting the CCSDT(Q)/CBS limit for the neutral $H_mX{\bullet\bullet\bullet}H_mY$, cationic $H_{m+1}X^+{\bullet\bullet\bullet}H_mY$, and anionic $H_mX{\bullet\bullet\bullet}H_{m-1}Y^-$ complexes and CCSD(T)/CBS limit for the large complexes, including a core-correlation correction, a relativistic correction, and the diagonal Born-Oppenheimer correction where appropriate.[a,b]

Neutral $H_mX{\bullet\bullet\bullet}H_mY$ Complexes

**Complex**	ΔE	ΔE_{strain}[c]	ΔE_{int}^{CP}	**Complex**	ΔE	ΔE_{strain}[c]	ΔE_{int}^{CP}	**Complex**	ΔE	ΔE_{strain}[c]	ΔE_{int}^{CP}
$NH_3{\bullet\bullet\bullet}NH_3$	–3.10	0.04	–3.14	$H_2O{\bullet\bullet\bullet}H_2O$	–5.02	0.05	–5.07	$HF{\bullet\bullet\bullet}HF$	–4.61	0.05	–4.66
$NH_3{\bullet\bullet\bullet}PH_3$	–1.79	0.00	–1.78	$H_2O{\bullet\bullet\bullet}H_2S$	–3.02	–0.04	–2.98	$HF{\bullet\bullet\bullet}HCl$	–3.06	–0.05	–3.01
$NH_3{\bullet\bullet\bullet}AsH_3$	[d]	[d]	[d]	$H_2O{\bullet\bullet\bullet}H_2Se$	–2.94	–0.22	–2.71	$HF{\bullet\bullet\bullet}HBr$	–3.02	–0.20	–2.82

Cationic $H_{m+1}X^+{\bullet\bullet\bullet}H_mY$ Complexes

**Complex**	ΔE	ΔE_{strain}	ΔE_{int}^{CP}	**Complex**	ΔE	ΔE_{strain}	ΔE_{int}^{CP}	**Complex**	ΔE	ΔE_{strain}	ΔE_{int}^{CP}
$NH_4^+{\bullet\bullet\bullet}NH_3$	–26.25	3.41	–29.66	$H_3O^+{\bullet\bullet\bullet}H_2O$	–33.75	18.47	–52.22	$H_2F^+{\bullet\bullet\bullet}HF$	–32.74	12.32	–45.06
$NH_4^+{\bullet\bullet\bullet}PH_3$	–14.11	1.23	–15.34	$H_3O^+{\bullet\bullet\bullet}H_2S$	–24.07	6.70	–30.77	$H_2F^+{\bullet\bullet\bullet}HCl$	–34.52	43.74	–78.26
$NH_4^+{\bullet\bullet\bullet}AsH_3$	–12.29	0.82	–13.11	$H_3O^+{\bullet\bullet\bullet}H_2Se$	–24.06	5.55	–29.61	$H_2F^+{\bullet\bullet\bullet}HBr$	–36.54	52.73	–89.27

Anionic $H_mX{\bullet\bullet\bullet}H_{m-1}Y^{-1}$ Complexes

**Complex**	ΔE	ΔE_{strain}	ΔE_{int}^{CP}	**Complex**	ΔE	ΔE_{strain}	ΔE_{int}^{CP}	**Complex**	ΔE	ΔE_{strain}	ΔE_{int}^{CP}
$NH_3{\bullet\bullet\bullet}NH_2^-$	–13.75	0.92	–14.67	$H_2O{\bullet\bullet\bullet}OH^-$	–26.89	7.73	–34.62	$HF{\bullet\bullet\bullet}F^-$	–44.04	21.13	–65.17
$NH_3{\bullet\bullet\bullet}PH_2^-$	–7.65	0.22	–7.87	$H_2O{\bullet\bullet\bullet}SH^-$	–14.99	0.68	–15.68	$HF{\bullet\bullet\bullet}Cl^-$	–23.76	1.49	–25.25
$NH_3{\bullet\bullet\bullet}AsH_2^-$	–7.31	0.07	–7.37	$H_2O{\bullet\bullet\bullet}SeH^-$	–13.59	0.34	–13.94	$HF{\bullet\bullet\bullet}Br^-$	–20.47	0.87	–21.35

Large Complexes

**Complex**	ΔE	ΔE_{strain}	ΔE_{int}^{CP}	**Complex**	ΔE	ΔE_{strain}	ΔE_{int}^{CP}
$H_2CO{\bullet\bullet\bullet}$**Am–O**	–4.95	0.11	–5.06	$H_2CO{\bullet\bullet\bullet}$**Am–S**	–5.59	0.10	–5.69
$H_2CO{\bullet\bullet\bullet}$**Ur–O**	–5.56	0.31	–5.87	$H_2CO{\bullet\bullet\bullet}$**Ur–S**	–6.93	0.25	–7.18
$H_2CO{\bullet\bullet\bullet}$**Ur–O**[e]	–6.33	0.10	–6.44	$H_2CO{\bullet\bullet\bullet}$**Ur–S**[e]	–7.33	0.06	–7.38
$H_2CO{\bullet\bullet\bullet}$**Delt–O**[e]	–6.53	0.12	–6.65	$H_2CO{\bullet\bullet\bullet}$**Delt–S**[e]	–6.93	0.18	–7.11
$H_2CO{\bullet\bullet\bullet}$**Squar–O**[e]	–8.51	0.22	–8.73	$H_2CO{\bullet\bullet\bullet}$**Squar–S**[e]	–9.24	0.26	–9.50

[a] Geometries computes at CCSD(T)/aVTZ for the neutral $H_mX{\bullet\bullet\bullet}H_mY$, cationic $H_{m+1}X^+{\bullet\bullet\bullet}H_mY$, and anionic $H_mX{\bullet\bullet\bullet}H_{m-1}Y^-$ complexes, CCSD(T)/jul-VTZ for $H_2CO{\bullet\bullet\bullet}$**Am–X** and $H_2CO{\bullet\bullet\bullet}$**Ur–X** (X = O, S), and CCSD(T)/jul-VDZ for $H_2CO{\bullet\bullet\bullet}$**Delt–X** and $H_2CO{\bullet\bullet\bullet}$**Squar–X** (X = O, S). [b] See Supporting Information Tables S17–51 for complete focal-point analysis. [c] In the case of small structural deformation, the strain energy can be negative when the energy penalty to deform the fragments upon hydrogen bonding is smaller than the additive corrections. [d] Geometry could not be located. [e] Computed using C_{2v} symmetry to mimic supramolecular motifs.

Table S46. Mean error (ME), mean absolute error (MAE), and largest absolute deviation (LAD) for the hydrogen bond energies of the neutral $H_mX{\cdots}H_mY$ complexes without counterpoise corrections, computed using various density functional approximations at ZORA-DFT/TZ2P//CCSD(T)/aVTZ compared to FPA methods targeting CCSDT(Q)/CBS//CCSD(T)/aVTZ.

Class	Functional	ME	MAE	LAD	Class	Functional	ME	MAE	LAD
**LDA**	VWN	–2.4	2.4	3.0	**Meta-Hybrid**	M06	0.4	0.4	0.4
						M06-2X	–0.1	0.1	0.3
**GGA**	BP86	0.7	0.7	1.5		M06-HF	0.2	0.2	0.5
	BLYP	0.7	0.7	1.6		TPSSH	0.4	0.4	1.0
	BEE	0.9	0.9	1.6					
	PW91	–0.5	0.5	0.8	**Double-Hybrid**	B2KPLYP	–0.3	0.4	1.1
	PBE	–0.2	0.4	0.6		B2TPLYP	–0.1	0.3	0.8
	PBEsol	–0.7	0.8	1.1		B2PLYP	0.0	0.3	0.8
	RPBE	0.8	0.8	1.4		LS1-TPSS	–0.4	0.5	1.2
	revPBE	1.1	1.1	1.7		mPW2KPLYP	–0.5	0.6	1.3
	mPBE	0.0	0.2	0.8		mPW2PLYP	–0.4	0.4	1.0
	mPW	0.5	0.5	1.2		PBE0-DH	–0.1	0.2	0.5
	HTBS	1.1	1.1	1.9					
	OLYP	1.9	1.9	2.6		rev-DSD-BLYP-D4	–0.5	0.5	1.2
	OPBE	2.5	2.5	3.1		rev-DSD-PBE-D4	–0.4	0.4	0.9
	XLYP	0.4	0.4	1.3		rev-DSD-PBEP86-D4	–0.3	0.3	0.8
	BP86-D4	0.0	0.2	0.3	**Range-Separated**	CAM-B3LYP	0.0	0.4	0.8
	BLYP-D4	–0.2	0.3	0.5		CAMY-B3LYP	–0.2	0.4	0.8
	PBE-D4	–0.7	0.7	0.9		ωB97	–0.6	0.6	0.8
	OLYP-D4	0.2	0.2	0.8		ωB97X	–0.5	0.5	0.7
	OPBE-D4	0.8	0.8	1.7					
	BLYP-D3(BJ)	–0.3	0.4	0.8		ωB97X-D4	–0.6	0.6	0.8
**Meta-GGA**	M06L	0.2	0.2	0.4					
	MVS	–0.1	0.3	0.5					
	TPSS	0.3	0.3	1.0					
	revTPSS	0.3	0.3	0.8					
**Hybrid**	B3LYP	0.5	0.5	1.3					
	B3LYP*	0.3	0.3	1.2					
	B1LYP	0.5	0.5	1.3					
	B1PW91	1.1	1.1	1.7					
	BHandH	–1.9	1.9	2.8					
	BHandHLYP	0.2	0.3	0.9					
	KMLYP	–1.0	1.0	1.7					
	O3LYP	–3.2	3.2	4.2					
	OPBE0	2.0	2.0	2.5					
	PBE0	0.0	0.2	0.6					
	mPW1PW	0.5	0.5	1.1					
	mPW1K	0.4	0.4	1.0					
	S12H	–0.5	0.5	0.6					
	X3LYP	0.1	0.3	0.9					
	B3LYP-D4	–0.2	0.3	0.5					
	PBE0-D4	–0.4	0.4	0.7					

Table S47. Mean error (ME), mean absolute error (MAE), and largest absolute deviation (LAD) for the hydrogen bond energies of the neutral H_mX•••H_mY complexes with counterpoise corrections, computed using various density functional approximations at ZORA-DFT/TZ2P//CCSD(T)/aVTZ compared to FPA methods targeting CCSDT(Q)/CBS//CCSD(T)/aVTZ.

Class	Functional	ME	MAE	LAD	Class	Functional	ME	MAE	LAD
**LDA**	VWN	–2.2	2.2	2.8	**Meta-Hybrid**	M06	0.6	0.6	0.7
						M06-2X	0.1	0.1	0.3
**GGA**	BP86	0.9	0.9	1.6		M06-HF	0.5	0.5	0.7
	BLYP	0.9	0.9	1.7		TPSSH	0.6	0.6	1.1
	BEE	1.2	1.2	1.8					
	PW91	–0.2	0.3	0.6	**Double-Hybrid**	B2KPLYP	0.4	0.4	0.7
	PBE	0.0	0.2	0.7		B2TPLYP	0.5	0.5	0.9
	PBEsol	–0.5	0.6	0.9		B2PLYP	0.6	0.6	1.0
	RPBE	1.1	1.1	1.6		LS1-TPSS	0.5	0.5	0.6
	revPBE	1.3	1.3	1.9		mPW2KPLYP	0.2	0.2	0.5
	mPBE	0.3	0.3	0.9		mPW2PLYP	0.2	0.2	0.6
	mPW	0.7	0.7	1.3		PBE0-DH	0.3	0.3	0.7
	HTBS	1.4	1.4	2.0					
	OLYP	2.3	2.3	2.9		rev-DSD-BLYP-D4	0.3	0.3	0.5
	OPBE	2.8	2.8	3.4		rev-DSD-PBE-D4	0.4	0.4	0.5
	XLYP	0.6	0.6	1.4		rev-DSD-PBEP86-D4	0.5	0.5	0.6
	BP86-D4	0.2	0.2	0.6	**Range-Separated**	CAM-B3LYP	0.2	0.3	0.8
	BLYP-D4	0.0	0.1	0.3		CAMY-B3LYP	0.0	0.3	0.7
	PBE-D4	–0.4	0.4	0.7		ωB97	–0.3	0.3	0.5
	OLYP-D4	0.6	0.6	1.1		ωB97X	–0.3	0.3	0.4
	OPBE-D4	1.1	1.1	2.0					
	BLYP-D3(BJ)	0.0	0.2	0.4		ωB97X-D4	–0.4	0.4	0.5
**Meta-GGA**	M06L	0.5	0.5	0.8					
	MVS	0.2	0.3	0.6					
	TPSS	0.6	0.6	1.1					
	revTPSS	0.5	0.5	0.9					
**Hybrid**	B3LYP	0.7	0.7	1.4					
	B3LYP*	0.5	0.5	1.2					
	B1LYP	0.8	0.8	1.4					
	B1PW91	1.3	1.3	1.8					
	BHandH	–1.6	1.6	2.4					
	BHandHLYP	0.5	0.5	0.9					
	KMLYP	–0.7	0.7	1.3					
	O3LYP	–3.0	3.0	3.9					
	OPBE0	2.2	2.2	2.6					
	PBE0	0.2	0.2	0.7					
	mPW1PW	0.7	0.7	1.2					
	mPW1K	0.7	0.7	1.1					
	S12H	–0.3	0.3	0.4					
	X3LYP	0.4	0.4	1.0					
	B3LYP-D4	0.0	0.1	0.2					
	PBE0-D4	–0.2	0.2	0.4					

Table S48. Mean error (ME), mean absolute error (MAE), and largest absolute deviation (LAD) for the hydrogen bond energies of the cationic $H_{m+1}X^{+}$•••H_mY complexes without counterpoise corrections, computed using various density functional approximations at ZORA-DFT/TZ2P//CCSD(T)/aVTZ compared to FPA methods targeting CCSDT(Q)/CBS//CCSD(T)/aVTZ.

Class	Functional	ME	MAE	LAD	Class	Functional	ME	MAE	LAD
**LDA**	VWN	–8.2	8.2	10.6	**Meta-Hybrid**	M06	–1.1	1.1	3.8
						M06-2X	–1.1	1.1	2.7
**GGA**	BP86	–3.0	3.0	5.1		M06-HF	–0.7	2.3	5.1
	BLYP	–2.3	2.5	5.4		TPSSH	–2.4	2.4	4.3
	BEE	–2.5	2.5	4.9					
	PW91	–4.4	4.4	6.6	**Double-Hybrid**	B2KPLYP	–1.9	1.9	3.4
	PBE	–4.0	4.0	6.3		B2TPLYP	–1.8	1.8	3.4
	PBEsol	–5.6	5.6	7.5		B2PLYP	–1.9	1.9	3.6
	RPBE	–2.0	2.1	4.9		LS1-TPSS	–2.0	2.0	3.4
	revPBE	–1.9	2.0	4.7		mPW2KPLYP	–2.1	2.1	3.6
	mPBE	–3.5	3.5	6.0		mPW2PLYP	–2.1	2.1	3.8
	mPW	–3.2	3.2	5.7		PBE0-DH	–2.2	2.2	3.3
	HTBS	–2.8	2.8	4.8					
	OLYP	–0.6	1.6	3.5		rev-DSD-BLYP-D4	–2.1	2.1	3.8
	OPBE	–0.6	1.5	2.6		rev-DSD-PBE-D4	–2.1	2.1	3.7
	XLYP	–2.5	2.6	5.9		rev-DSD-PBEP86-D4	–1.7	1.7	3.4
	BP86-D4	–3.8	3.8	5.8	**Range-Separated**	CAM-B3LYP	–2.2	2.2	3.7
	BLYP-D4	–3.5	3.5	6.6		CAMY-B3LYP	–2.5	2.5	4.1
	PBE-D4	–4.6	4.6	6.8		ωB97	–2.2	2.2	5.1
	OLYP-D4	–3.0	3.0	6.1		ωB97X	–2.0	2.0	4.3
	OPBE-D4	–3.0	3.0	5.4					
	BLYP-D3(BJ)	–3.8	3.8	6.8		ωB97X-D4	–2.1	2.1	4.3
**Meta-GGA**	M06L	–2.0	2.0	6.2					
	MVS	–2.4	2.4	3.5					
	TPSS	–2.9	2.9	5.1					
	revTPSS	–2.2	2.2	5.0					
**Hybrid**	B3LYP	–1.8	1.9	3.9					
	B3LYP*	–2.4	2.4	4.5					
	B1LYP	–1.4	1.5	3.5					
	B1PW91	–1.4	1.4	2.9					
	BHandH	–4.9	4.9	7.4					
	BHandHLYP	–0.8	0.9	2.2					
	KMLYP	–3.1	3.1	5.0					
	O3LYP	–9.5	9.5	12.9					
	OPBE0	–0.1	0.8	1.3					
	PBE0	–2.7	2.7	3.9					
	mPW1PW	–2.1	2.1	3.5					
	mPW1K	–1.4	1.4	2.5					
	S12H	–2.8	2.8	5.2					
	X3LYP	–2.2	2.2	4.1					
	B3LYP-D4	–2.7	2.7	4.8					
	PBE0-D4	–3.2	3.2	4.4					

Table S49. Mean error (ME), mean absolute error (MAE), and largest absolute deviation (LAD) for the hydrogen bond energies of the cationic $H_{m+1}X^{+}\cdots H_mY$ complexes with counterpoise corrections, computed using various density functional approximations at ZORA-DFT/TZ2P//CCSD(T)/aVTZ compared to FPA methods targeting CCSDT(Q)/CBS//CCSD(T)/aVTZ.

Class	Functional	ME	MAE	LAD	Class	Functional	ME	MAE	LAD
**LDA**	VWN	–7.7	7.7	10.1	**Meta-Hybrid**	M06	–0.5	0.7	2.7
						M06-2X	–0.6	0.8	2.1
**GGA**	BP86	–2.5	2.5	4.2		M06-HF	0.1	2.1	6.4
	BLYP	–1.8	2.1	4.7		TPSSH	–1.9	1.9	3.5
	BEE	–2.0	2.1	4.0					
	PW91	–3.9	3.9	5.8	**Double-Hybrid**	B2KPLYP	–0.1	0.4	1.3
	PBE	–3.5	3.5	5.4		B2TPLYP	–0.4	0.6	1.8
	PBEsol	–5.1	5.1	6.9		B2PLYP	–0.6	0.8	2.1
	RPBE	–1.5	1.6	4.1		LS1-TPSS	0.2	0.4	0.9
	revPBE	–1.4	1.6	3.9		mPW2KPLYP	–0.4	0.5	1.6
	mPBE	–3.0	3.0	5.1		mPW2PLYP	–0.9	0.9	2.4
	mPW	–2.7	2.7	4.9		PBE0-DH	–1.3	1.3	2.3
	HTBS	–2.3	2.3	4.1					
	OLYP	–0.1	1.6	2.7		rev-DSD-BLYP-D4	–0.1	0.6	1.4
	OPBE	0.0	1.5	1.8		rev-DSD-PBE-D4	–0.1	0.6	1.3
	XLYP	–2.1	2.2	5.1		rev-DSD-PBEP86-D4	0.3	0.6	1.2
	BP86-D4	–3.3	3.3	5.1	**Range-Separated**	CAM-B3LYP	–1.7	1.7	3.3
	BLYP-D4	–3.0	3.0	5.8		CAMY-B3LYP	–2.0	2.0	3.6
	PBE-D4	–4.1	4.1	6.0		ωB97	–1.7	1.7	4.2
	OLYP-D4	–2.5	2.5	5.2		ωB97X	–1.5	1.5	3.4
	OPBE-D4	–2.4	2.4	4.7					
	BLYP-D3(BJ)	–3.3	3.3	6.0		ωB97X-D4	–1.6	1.6	3.4
**Meta-GGA**	M06L	–1.2	1.2	4.9					
	MVS	–1.7	1.7	2.9					
	TPSS	–2.3	2.3	4.3					
	revTPSS	–1.7	1.7	4.1					
**Hybrid**	B3LYP	–1.3	1.4	3.1					
	B3LYP*	–1.9	1.9	3.7					
	B1LYP	–0.9	1.2	2.7					
	B1PW91	–0.9	1.0	2.3					
	BHandH	–4.4	4.4	6.8					
	BHandHLYP	–0.3	0.8	1.7					
	KMLYP	–2.5	2.5	4.3					
	O3LYP	–9.0	9.0	12.3					
	OPBE0	0.5	0.8	1.2					
	PBE0	–2.2	2.2	3.5					
	mPW1PW	–1.5	1.5	3.0					
	mPW1K	–0.9	0.9	2.1					
	S12H	–2.3	2.3	4.3					
	X3LYP	–1.7	1.7	3.4					
	B3LYP-D4	–2.1	2.1	3.9					
	PBE0-D4	–2.7	2.7	3.9					

Table S50. Mean error (ME), mean absolute error (MAE), and largest absolute deviation (LAD) for the hydrogen bond energies of the anionic $H_mX•••H_{m-1}Y^-$ complexes without counterpoise corrections, computed using various density functional approximations at ZORA-DFT/TZ2P//CCSD(T)/aVTZ compared to FPA methods targeting CCSDT(Q)/CBS//CCSD(T)/aVTZ.

Class	Functional	ME	MAE	LAD	Class	Functional	ME	MAE	LAD
**LDA**	VWN	–8.8	8.8	17.4	**Meta-Hybrid**	M06	–1.8	1.8	5.0
						M06-2X	–2.8	2.8	8.2
**GGA**	BP86	–3.1	3.1	8.7		M06-HF	–3.6	3.6	11.5
	BLYP	–2.7	2.7	8.0		TPSSH	–2.7	2.7	7.6
	BEE	–2.6	2.6	7.5					
	PW91	–4.7	4.7	10.3	**Double-Hybrid**	B2KPLYP	–2.9	2.9	7.8
	PBE	–4.3	4.3	9.8		B2TPLYP	–2.7	2.7	7.7
	PBEsol	–5.8	5.8	12.7		B2PLYP	–2.7	2.7	7.7
	RPBE	–2.3	2.3	6.5		LS1-TPSS	–3.1	3.1	8.0
	revPBE	–2.1	2.1	6.5		mPW2KPLYP	–3.1	3.1	8.2
	mPBE	–3.8	3.8	8.9		mPW2PLYP	–3.0	3.0	8.1
	mPW	–3.3	3.3	8.5		PBE0-DH	–2.9	2.9	7.7
	HTBS	–2.8	2.8	8.4					
	OLYP	–0.7	1.9	5.0		rev-DSD-BLYP-D4	–3.0	3.0	7.8
	OPBE	–0.3	1.9	4.3		rev-DSD-PBE-D4	–3.0	3.0	7.5
	XLYP	–3.0	3.0	8.1		rev-DSD-PBEP86-D4	–2.6	2.6	7.0
	BP86-D4	–3.9	3.9	9.1	**Range-Separated**	CAM-B3LYP	–2.9	2.9	8.9
	BLYP-D4	–3.8	3.8	8.6		CAMY-B3LYP	–3.1	3.1	9.0
	PBE-D4	–4.9	4.9	10.0		ωB97	–2.9	2.9	7.8
	OLYP-D4	–3.1	3.1	7.6		ωB97X	–2.9	2.9	7.5
	OPBE-D4	–2.7	2.8	7.3					
	BLYP-D3(BJ)	–3.7	3.7	8.3		ωB97X-D4	–3.0	3.0	7.6
**Meta-GGA**	M06L	–1.3	1.8	4.8					
	MVS	–3.0	3.0	8.6					
	TPSS	–3.0	3.0	8.2					
	revTPSS	–2.7	2.7	7.7					
**Hybrid**	B3LYP	–2.3	2.4	7.4					
	B3LYP*	–2.8	2.8	8.3					
	B1LYP	–2.0	2.2	7.0					
	B1PW91	–1.6	2.1	6.3					
	BHandH	–5.9	5.9	13.2					
	BHandHLYP	–1.6	2.0	6.4					
	KMLYP	–3.9	3.9	10.0					
	O3LYP	–10.0	10.0	19.6					
	OPBE0	–0.2	1.8	4.1					
	PBE0	–3.1	3.1	8.2					
	mPW1PW	–2.4	2.4	7.2					
	mPW1K	–1.9	2.1	6.5					
	S12H	–2.9	2.9	6.8					
	X3LYP	–2.7	2.7	8.0					
	B3LYP-D4	–3.2	3.2	7.9					
	PBE0-D4	–3.6	3.6	8.4					

Table S51. Mean error (ME), mean absolute error (MAE), and largest absolute deviation (LAD) for the hydrogen bond energies of the anionic $H_mX{\cdots}H_{m-1}Y^-$ complexes with counterpoise corrections, computed using various density functional approximations at ZORA-DFT/TZ2P//CCSD(T)/aVTZ compared to FPA methods targeting CCSDT(Q)/CBS//CCSD(T)/aVTZ.

Class	Functional	ME	MAE	LAD	Class	Functional	ME	MAE	LAD
**LDA**	VWN	–6.9	6.9	13.5	**Meta-Hybrid**	M06	–0.4	0.8	2.3
						M06-2X	–1.7	1.7	6.0
**GGA**	BP86	–1.2	1.5	4.8		M06-HF	–2.5	2.5	9.3
	BLYP	–0.4	1.0	3.3		TPSSH	–1.1	1.3	4.5
	BEE	–0.6	1.0	3.3					
	PW91	–2.7	2.7	6.1	**Double-Hybrid**	B2KPLYP	–0.5	1.1	3.0
	PBE	–2.3	2.3	5.5		B2TPLYP	–0.5	1.1	3.2
	PBEsol	–4.0	4.0	8.7		B2PLYP	–0.4	1.1	3.2
	RPBE	0.0	0.7	1.8		LS1-TPSS	–0.5	1.0	2.6
	revPBE	0.0	0.9	2.1		mPW2KPLYP	–0.7	1.1	3.2
	mPBE	–1.7	1.7	4.5		mPW2PLYP	–0.8	1.1	3.7
	mPW	–1.4	1.4	4.5		PBE0-DH	–1.3	1.4	4.7
	HTBS	–0.9	1.3	4.4					
	OLYP	1.8	1.8	2.8		rev-DSD-BLYP-D4	–0.4	1.0	2.4
	OPBE	1.8	1.8	2.8		rev-DSD-PBE-D4	–0.4	0.9	2.1
	XLYP	–0.7	1.0	3.4		rev-DSD-PBEP86-D4	0.0	0.8	1.6
	BP86-D4	–2.1	2.1	5.2	**Range-Separated**	CAM-B3LYP	–1.3	1.7	5.7
	BLYP-D4	–1.6	1.6	4.0		CAMY-B3LYP	–1.5	1.7	5.7
	PBE-D4	–2.8	2.8	5.7		ωB97	–1.5	1.6	4.7
	OLYP-D4	–0.6	0.9	2.0		ωB97X	–1.4	1.5	4.6
	OPBE-D4	–0.7	1.0	2.8					
	BLYP-D3(BJ)	–1.5	1.5	3.7		ωB97X-D4	–1.5	1.5	4.6
**Meta-GGA**	M06L	0.0	1.1	2.2					
	MVS	–1.7	1.9	6.0					
	TPSS	–1.3	1.4	4.5					
	revTPSS	–1.0	1.2	4.0					
**Hybrid**	B3LYP	–0.6	1.2	4.1					
	B3LYP*	–1.0	1.4	4.7					
	B1LYP	–0.3	1.2	3.8					
	B1PW91	–0.3	1.3	3.8					
	BHandH	–4.6	4.6	11.0					
	BHandHLYP	–0.3	1.4	4.2					
	KMLYP	–2.8	2.8	8.2					
	O3LYP	–8.4	8.4	16.3					
	OPBE0	1.3	1.7	2.5					
	PBE0	–1.6	1.7	5.3					
	mPW1PW	–1.0	1.3	4.5					
	mPW1K	–0.8	1.3	4.5					
	S12H	–1.3	1.3	3.6					
	X3LYP	–1.0	1.3	4.6					
	B3LYP-D4	–1.4	1.4	4.5					
	PBE0-D4	–2.2	2.2	5.5					

Table S52. Mean error (ME), mean absolute error (MAE), and largest absolute deviation (LAD) for the hydrogen bond energies of the large complexes without counterpoise correction, computed using various density functional approximations at ZORA-DFT/TZ2P//jul-VTZ for H_2CO•••**Am–X** and H_2CO•••**Ur–X** (X = O, S), and ZORA-DFT/TZ2P//CCSD(T)/jul-VDZ for H_2CO•••**Delt–X** and H_2CO•••**Squar–X** (X = O, S) compared to FPA methods targeting CCSD(T)/CBS//CCSD(T)/aVTZ and CCSD(T)/CBS//CCSD(T)/aVDZ, respectively.

Class	Functional	ME	MAE	LAD	Class	Functional	ME	MAE	LAD
**LDA**	VWN	–2.1	2.1	2.7	**Meta-Hybrid**	M06	1.2	1.2	1.6
						M06-2X	0.4	0.4	0.7
**GGA**	BP86	2.5	2.5	3.2		M06-HF	0.4	0.4	1.0
	BLYP	2.4	2.4	3.0		TPSSH	1.8	1.8	2.4
	BEE	3.0	3.0	3.6					
	PW91	0.9	0.9	1.5	**Double-Hybrid**	B2KPLYP	–0.4	0.4	0.7
	PBE	1.2	1.2	1.8		B2TPLYP	0.1	0.1	0.4
	PBEsol	0.5	0.5	1.2		B2PLYP	0.4	0.4	0.7
	RPBE	2.8	2.8	3.3		LS1-TPSS	–0.6	0.6	1.0
	revPBE	3.2	3.2	3.8		mPW2KPLYP	–0.7	0.7	1.0
	mPBE	1.6	1.6	2.1		mPW2PLYP	–0.2	0.2	0.4
	mPW	2.3	2.3	3.0		PBE0-DH	0.4	0.4	0.8
	HTBS	3.5	3.5	4.2					
	OLYP	4.3	4.3	4.8		rev-DSD-BLYP-D4	–0.9	0.9	1.3
	OPBE	5.4	5.4	6.0		rev-DSD-PBE-D4	–0.6	0.6	0.9
	XLYP	1.9	1.9	2.5		rev-DSD-PBEP86-D4	–0.4	0.4	0.6
	BP86-D4	0.9	0.9	1.3	**Range-Separated**	CAM-B3LYP	0.6	0.6	1.0
	BLYP-D4	0.3	0.3	0.6		CAMY-B3LYP	0.4	0.4	0.8
	PBE-D4	0.1	0.3	0.5		ωB97	–0.4	0.4	0.9
	OLYP-D4	0.9	0.9	1.4		ωB97X	–0.2	0.3	0.6
	OPBE-D4	1.7	1.7	2.3					
	BLYP-D3(BJ)	0.4	0.4	0.8		ωB97X-D4	–0.5	0.5	1.0
**Meta-GGA**	M06L	1.0	1.0	1.3					
	MVS	0.6	0.6	0.8					
	TPSS	2.0	2.0	2.6					
	revTPSS	1.8	1.8	2.3					
**Hybrid**	B3LYP	1.6	1.6	2.1					
	B3LYP*	1.4	1.4	2.0					
	B1LYP	1.5	1.5	2.0					
	B1PW91	2.6	2.6	3.3					
	BHandH	–2.5	2.5	3.2					
	BHandHLYP	0.6	0.6	1.0					
	KMLYP	–1.2	1.2	2.0					
	O3LYP	–3.7	3.7	4.5					
	OPBE0	4.1	4.1	4.6					
	PBE0	1.0	1.0	1.5					
	mPW1PW	1.8	1.8	2.3					
	mPW1K	1.3	1.3	1.8					
	S12H	–0.4	0.4	0.5					
	X3LYP	1.0	1.0	1.5					
	B3LYP-D4	0.0	0.1	0.2					
	PBE0-D4	0.0	0.1	0.3					

Table S53. Mean error (ME), mean absolute error (MAE), and largest absolute deviation (LAD) for the hydrogen bond energies of the large complexes with counterpoise correction, computed using various density functional approximations at ZORA-DFT/TZ2P//jul-VTZ for H_2CO•••**Am–X** and H_2CO•••**Ur–X** (X = O, S), and ZORA-DFT/TZ2P//CCSD(T)/jul-VDZ for H_2CO•••**Delt–X** and H_2CO•••**Squar–X** (X = O, S) compared to FPA methods targeting CCSD(T)/CBS//CCSD(T)/aVTZ and CCSD(T)/CBS//CCSD(T)/aVDZ, respectively.

Class	Functional	ME	MAE	LAD	Class	Functional	ME	MAE	LAD
**LDA**	VWN	–1.8	1.8	2.4	**Meta-Hybrid**	M06	1.5	1.5	1.9
						M06-2X	0.6	0.6	1.0
**GGA**	BP86	2.7	2.7	3.5		M06-HF	0.7	0.7	1.3
	BLYP	2.6	2.6	3.2		TPSSH	2.1	2.1	2.6
	BEE	3.2	3.2	3.9					
	PW91	1.2	1.2	1.7	**Double-Hybrid**	B2KPLYP	0.8	0.8	1.0
	PBE	1.5	1.5	2.0		B2TPLYP	1.1	1.1	1.3
	PBEsol	0.7	0.7	1.5		B2PLYP	1.3	1.3	1.6
	RPBE	3.1	3.1	3.6		LS1-TPSS	0.9	0.9	1.1
	revPBE	3.5	3.5	4.1		mPW2KPLYP	0.5	0.5	0.7
	mPBE	1.8	1.8	2.4		mPW2PLYP	0.6	0.6	0.9
	mPW	2.5	2.5	3.2		PBE0-DH	1.0	1.0	1.4
	HTBS	3.8	3.8	4.5					
	OLYP	4.7	4.7	5.2		rev-DSD-BLYP-D4	0.4	0.4	0.6
	OPBE	5.7	5.7	6.3		rev-DSD-PBE-D4	0.7	0.7	1.0
	XLYP	2.2	2.2	2.7		rev-DSD-PBEP86-D4	1.0	1.0	1.2
	BP86-D4	1.1	1.1	1.6	**Range-Separated**	CAM-B3LYP	0.8	0.8	1.2
	BLYP-D4	0.6	0.6	0.8		CAMY-B3LYP	0.7	0.7	1.1
	PBE-D4	0.4	0.4	0.7		ωB97	–0.1	0.2	0.6
	OLYP-D4	1.2	1.2	1.7		ωB97X	0.1	0.2	0.4
	OPBE-D4	2.1	2.1	2.6					
	BLYP-D3(BJ)	0.7	0.7	1.0		ωB97X-D4	–0.2	0.2	0.6
**Meta-GGA**	M06L	1.5	1.5	1.8					
	MVS	1.0	1.0	1.2					
	TPSS	2.2	2.2	2.8					
	revTPSS	2.0	2.0	2.5					
**Hybrid**	B3LYP	1.9	1.9	2.4					
	B3LYP*	1.7	1.7	2.3					
	B1LYP	1.8	1.8	2.3					
	B1PW91	2.9	2.9	3.6					
	BHandH	–2.2	2.2	2.9					
	BHandHLYP	0.9	0.9	1.4					
	KMLYP	–0.9	0.9	1.6					
	O3LYP	–3.5	3.5	4.2					
	OPBE0	4.4	4.4	4.9					
	PBE0	1.3	1.3	1.8					
	mPW1PW	2.0	2.0	2.6					
	mPW1K	1.6	1.6	2.2					
	S12H	–0.1	0.1	0.2					
	X3LYP	1.3	1.3	1.8					
	B3LYP-D4	0.3	0.3	0.5					
	PBE0-D4	0.3	0.3	0.6					

Table S54. Mean error (ME), mean absolute error (MAE), and largest absolute deviation (LAD) for the hydrogen bond energies without and with counterpoise correction computed at ZORA-DFT/(AUG-)TZ2P of a) the cationic $H_{m+1}X^{+}$•••H_mY complexes; and b) the anionic H_mX•••$H_{m-1}Y^{-}$complexes relative to CCSDT(Q)/CBS//CCSD(T)/aVTZ on CCSD(T)/aVTZ equilibrium geometries.

		ΔE			ΔE_{CPC}		
**Class**	**Functional**	**ME**	**MAE**	**LAD**	**ME**	**MAE**	**LAD**
		Cationic H_mX•••$H_{m+1}X^{+}$ Complexes					
**GGA**	OLYP	–0.2	1.5	2.8	0.1	1.4	2.2
	BLYP-D4	–3.1	3.1	5.7	–2.8	2.8	5.1
	BLYP-D3(BJ)	–3.4	3.4	5.9	–3.2	3.2	5.4
**Hybrid**	B3LYP	–1.4	1.5	3.0	–1.1	1.3	2.4
	B3LYP-D4	–2.3	2.3	3.8	–2.0	2.0	3.3
**Meta–Hybrid**	M06-2X	–0.7	1.0	3.0	–0.5	1.0	2.6
		Anionic $H_{m-1}X^{-}$•••H_mX Complexes					
**GGA**	OLYP	2.3	2.3	3.6	2.9	2.9	4.0
	BLYP-D4	–0.8	0.8	1.7	–0.3	0.4	0.7
	BLYP-D3(BJ)	–0.7	0.7	1.7	–0.2	0.5	0.7
**Hybrid**	B3LYP	0.3	0.6	0.8	0.6	0.7	1.1
	B3LYP-D4	–0.6	0.6	1.4	–0.3	0.3	1.0
**Meta–Hybrid**	M06-2X	–0.9	0.9	3.6	–0.7	0.8	3.4

Table S55. Mean error (ME), mean absolute error (MAE), and largest absolute deviation (LAD) for hydrogen-bond energies (in kcal mol^{-1}) without and with counterpoise corrections (CPC), and hydrogen bond distances (in Å), and the Cartesian root-mean-square deviation (RMSD) analysis of the complete hydrogen-bonded complex for selected DFT functionals. Computed at ZORA-DFT/TZ2P.

		ΔE			ΔE_{CPC}			$r_{X\cdots H}$			**Geometry**
**Class**	**Functional**	**ME**	**MAE**	**LAD**	**ME**	**MAE**	**LAD**	**ME**	**MAE**	**LAD**	**RMSD**
					Neutral $H_mX\cdots H_mY$ Complexes						
**GGA**	OLYP	1.5	1.5	2.0	1.8	1.8	2.4	0.277	0.277	0.532	0.2236
	BLYP-D4	–0.3	0.3	0.6	0.0	0.2	0.3	–0.026	0.026	0.068	0.0165
	BLYP-D3(BJ)	–0.3	0.4	0.8	–0.1	0.2	0.4	–0.026	0.026	0.071	0.0175
**Hybrid**	B3LYP	0.4	0.4	1.2	0.7	0.7	1.2	0.019	0.030	0.197	0.0500
	B3LYP-D4	–0.3	0.3	0.5	0.0	0.1	0.2	–0.020	0.020	0.060	0.0138
**Meta–Hybrid**	M06-2X	–0.1	0.1	0.3	0.1	0.1	0.3	–0.015	0.015	0.056	0.0142
					Cationic $H_mX\cdots H_{m+1}Y^+$ Complexes						
**GGA**	OLYP	–0.9	1.8	3.9	–0.4	1.8	3.1	–0.044	0.072	0.178	0.0460
	BLYP-D4	–4.0	4.0	7.3	–3.5	3.5	6.5	–0.057	0.094	0.221	0.0526
	BLYP-D3(BJ)	–4.4	4.4	7.5	–3.9	3.9	6.7	–0.058	0.095	0.216	0.0528
**Hybrid**	B3LYP	–1.9	2.0	4.1	–1.4	1.5	3.2	–0.025	0.045	0.132	0.0338
	B3LYP-D4	–2.8	2.8	4.9	–2.3	2.3	4.0	–0.036	0.057	0.144	0.0374
**Meta–Hybrid**	M06-2X	–1.2	1.2	2.6	–0.7	0.8	1.9	–0.011	0.045	0.115	0.0414
					Anionic $H_{m-1}X^-\cdots H_mY$ Complexes						
**GGA**	OLYP	–1.1	2.1	5.1	1.7	1.7	2.9	0.003	0.057	0.162	0.1336
	BLYP-D4	–4.2	4.2	8.9	–1.7	1.7	4.2	–0.051	0.057	0.156	0.0971
	BLYP-D3(BJ)	–4.2	4.2	8.6	–1.6	1.6	4.0	–0.041	0.047	0.140	0.0999
**Hybrid**	B3LYP	–2.4	2.5	7.5	–0.5	1.3	4.1	–0.029	0.032	0.160	0.0766
	B3LYP-D4	–3.3	3.3	7.9	–1.4	1.4	4.5	–0.040	0.043	0.165	0.0744
**Meta–Hybrid**	M06-2X	–3.0	3.0	8.2	–1.7	1.7	6.0	–0.034	0.036	0.149	0.0650
					Large Complexes						
**GGA**	OLYP	3.2	3.2	3.8	3.4	3.4	4.1	0.371	0.371	0.431	0.0531
	BLYP-D4	0.3	0.3	0.6	0.6	0.6	0.8	–0.003	0.003	0.011	0.0001
	BLYP-D3(BJ)	0.4	0.4	0.7	0.7	0.7	1.0	–0.004	0.004	0.012	0.0002
**Hybrid**	B3LYP	1.5	1.5	2.0	1.8	1.8	2.3	0.038	0.038	0.081	0.0158
	B3LYP-D4	0.0	0.1	0.2	0.3	0.3	0.5	–0.002	0.003	0.010	0.0005
**Meta–Hybrid**	M06-2X	0.4	0.4	0.8	0.7	0.7	1.1	0.000	0.005	0.020	0.0014
